# Supplementary material for: Sociodemographic risk factors of under-five stunting in Bangladesh: Assessing the role of interactions using a machine learning method
Source: PLoS One. 2021 Aug 31;16(8):e0256729. doi: 10.1371/journal.pone.0256729 (PMC8407547; doi:10.1371/journal.pone.0256729)
Supplement: Attachment — Submitted filename: Review of the manuscript PONE-D- 20-31337.pdf [file pone.0256729.s001.pdf]

Review of the manuscript: PONE-D- 20-31337

| | |
|---|---|
| Summary | Overall the manuscript is fine. The literature review has successfully addressed the objective of the research. The manuscript presented a nice application of machine learning method in public health research (data analysis) comparing with typical logistical model which is innovative idea. However, for a reader without having any prior knowledge of machine learning could be difficult to recognize the method. |
| Major issues | No major issues to be addressed. |
| Minor issues | a) Line 225-226: “Working mothers’ had a higher percentage of stunted children (39.9%)”-Are these working mothers from lower class? Have no education? Are they working in their own area and staying with their children or they migrate to other cities leaving children at home. Need a bit explanation otherwise it looks more generalized data.<br><br>b) Line 229: The paper mentioned two specific areas (Sylhet and Khulna) of Bangladesh where the prevalence of stunning were highest and lowest. Would you please explain which indicators were specifically worked for this variation between two areas?<br><br>c) Line 235: Figure 1, there is no figure attached.<br><br>d) In table 3, "Rules extracted from the classification tree" is a bit confusing. Is it a part logistical regression output?? If the objective of this table is to compare or find similarities then it can be placed in separate table.<br><br>e) Line 277: It would be better to understand when comparing two models, i.e. machine learning and logistic model in separate section. |
| Other comments | A simple definition with reference of Machine Learning method will be helpful for readers. |
