# Supplementary material for: Sociodemographic risk factors of under-five stunting in Bangladesh: Assessing the role of interactions using a machine learning method
Source: PLoS One. 2021 Aug 31;16(8):e0256729. doi: 10.1371/journal.pone.0256729 (PMC8407547; doi:10.1371/journal.pone.0256729)

PLOS ONE

Sociodemographic Risk Factors of Under-Five Stunting in Bangladesh: Assessing the Role of Interactions Using a Machine Learning Method

--Manuscript Draft--

**Manuscript Number:**	PONE-D-20-31337
**Article Type:**	Research Article
**Full Title:**	Sociodemographic Risk Factors of Under-Five Stunting in Bangladesh: Assessing the Role of Interactions Using a Machine Learning Method
**Short Title:**	Sociodemographic Risk Factors of Under-Five Stunting in Bangladesh: Assessing the Role of Interactions
**Corresponding Author:**	Mohaimen Mansur, Ph.D<br>University of Dhaka: Dhaka University<br>Dhaka, BANGLADESH
**Keywords:**	child stunting; malnutrition; machine learning; classification tree; public health; intervention; policy
**Abstract:**	Childhood stunting has been a persistent problem in Bangladesh with decades of interventions failing to reach the intended targets. Existing empirical studies investigating the problem in a socioeconomic context largely ignore the complex interplay of potential determinants that may be crucial for an intricate understanding of child malnutrition. This paper aims to demonstrate the importance of studying interactions among various sociodemographic risk factors of stunting with the help of an interpretable machine learning method. Data used for the analyses are extracted from the Bangladesh Demographic and Health Survey (BDHS) 2014 and pertain to a sample of 6,170 under-5 children. A classification tree was first constructed to identify important interaction-based rules that characterize children with different profiles of risk for stunting. Then binary logistic regression models were fitted to measure the importance of these interactions along with the individual risk factors. Results revealed that risk classifications defined by the interplay of multiple sociodemographic factors showed more extreme odds ratios (OR) of stunting than single factor ORs. The findings endorse the need for tailored-intervention programs for children based on their distinct risk profiles and sociodemographic characteristics. Such targeted efforts could prove to be cost-effective and more efficient in achieving the Health Nutrition and Population Sector Program (HNPSP) objective of limiting the prevalence of stunting in children within 25% by the year 2022 and aid in attaining the Sustainable Development Goal 2.2 of ending all forms of child malnutrition in Bangladesh.
**Order of Authors:**	Mohaimen Mansur, Ph.D
	Awan Afiaz
	Md. Saddam Hossain
**Additional Information:**	
**Question**	**Response**
**Financial Disclosure**<br><br>Enter a financial disclosure statement that describes the sources of funding for the work included in this submission. Review the submission guidelines for detailed requirements. View published research articles from *PLOS ONE* for specific examples.<br><br>This statement is required for submission	The author(s) received no specific funding for this work.

Powered by Editorial Manager® and ProduXion Manager® from Aries Systems Corporation

and **will appear in the published article** if the submission is accepted. Please make sure it is accurate.<br><br>**Unfunded studies**<br>Enter: *The author(s) received no specific funding for this work.*<br><br>**Funded studies**<br>Enter a statement with the following details:<br>• Initials of the authors who received each award<br>• Grant numbers awarded to each author<br>• The full name of each funder<br>• URL of each funder website<br>• Did the sponsors or funders play any role in the study design, data collection and analysis, decision to publish, or preparation of the manuscript?<br>• **NO** - Include this sentence at the end of your statement: *The funders had no role in study design, data collection and analysis, decision to publish, or preparation of the manuscript.*<br>• **YES** - Specify the role(s) played.<br><br>* typeset	
**Competing Interests**<br><br>Use the instructions below to enter a competing interest statement for this submission. On behalf of all authors, disclose any competing interests that could be perceived to bias this work—acknowledging all financial support and any other relevant financial or non-financial competing interests.<br><br>This statement **will appear in the published article** if the submission is accepted. Please make sure it is accurate. View published research articles from *PLOS ONE* for specific examples.	The authors have declared that no competing interests exist.

Powered by Editorial Manager® and ProduXion Manager® from Aries Systems Corporation

**NO authors have competing interests**<br><br>Enter: *The authors have declared that no competing interests exist.*<br><br>**Authors with competing interests**<br><br>Enter competing interest details beginning with this statement:<br><br>*I have read the journal's policy and the authors of this manuscript have the following competing interests: [insert competing interests here]*<br><br>* typeset	
**Ethics Statement**<br><br>Enter an ethics statement for this submission. This statement is required if the study involved:<br><br>• Human participants<br>• Human specimens or tissue<br>• Vertebrate animals or cephalopods<br>• Vertebrate embryos or tissues<br>• Field research<br><br>Write "N/A" if the submission does not require an ethics statement.<br><br>General guidance is provided below. Consult the submission guidelines for detailed instructions. **Make sure that all information entered here is included in the Methods section of the manuscript.**	This article does not contain any studies with human participants performed by any of the authors. The Bangladesh demographic and health Surveys were approved by ICF Macro Institutional Review Board and the National Research Ethics Committee of the Bangladesh Medical Research Council. A written consent about the survey was given by participants before the interview. All identification of the respondents was dis-identified before publishing data. The secondary data sets analysed during the current study are freely available upon request from the DHS website at http://dhsprogram.com/data/available-datasets.com. Searching 'Bangladesh DHS, 2014' in the DHS website will provide the survey data set.

Powered by Editorial Manager® and ProduXion Manager® from Aries Systems Corporation

**Format for specific study types**<br><br>**Human Subject Research (involving human participants and/or tissue)**<br>• Give the name of the institutional review board or ethics committee that approved the study<br>• Include the approval number and/or a statement indicating approval of this research<br>• Indicate the form of consent obtained (written/oral) or the reason that consent was not obtained (e.g. the data were analyzed anonymously)<br><br>**Animal Research (involving vertebrate animals, embryos or tissues)**<br>• Provide the name of the Institutional Animal Care and Use Committee (IACUC) or other relevant ethics board that reviewed the study protocol, and indicate whether they approved this research or granted a formal waiver of ethical approval<br>• Include an approval number if one was obtained<br>• If the study involved *non-human primates*, add *additional details* about animal welfare and steps taken to ameliorate suffering<br>• If anesthesia, euthanasia, or any kind of animal sacrifice is part of the study, include briefly which substances and/or methods were applied<br><br>**Field Research**<br><br>Include the following details if this study involves the collection of plant, animal, or other materials from a natural setting:<br>• Field permit number<br>• Name of the institution or relevant body that granted permission	
**Data Availability**<br><br>Authors are required to make all data underlying the findings described fully available, without restriction, and from the time of publication. PLOS allows rare exceptions to address legal and ethical concerns. See the PLOS Data Policy and FAQ for detailed information.	No - some restrictions will apply

A Data Availability Statement describing where the data can be found is required at submission. Your answers to this question constitute the Data Availability Statement and **will be published in the article**, if accepted.<br><br>**Important:** Stating 'data available on request from the author' is not sufficient. If your data are only available upon request, select 'No' for the first question and explain your exceptional situation in the text box.<br><br>Do the authors confirm that all data underlying the findings described in their manuscript are fully available without restriction?	
**Describe where the data may be found in full sentences. If you are copying our sample text, replace any instances of XXX with the appropriate details.**<br><br>• If the data are **held or will be held in a public repository**, include URLs, accession numbers or DOIs. If this information will only be available after acceptance, indicate this by ticking the box below. For example: *All XXX files are available from the XXX database (accession number(s) XXX, XXX.*).<br>• If the data are all contained **within the manuscript and/or Supporting Information files**, enter the following: *All relevant data are within the manuscript and its Supporting Information files.*<br>• If neither of these applies but you are able to provide **details of access elsewhere**, with or without limitations, please do so. For example:<br><br>*Data cannot be shared publicly because of [XXX]. Data are available from the XXX Institutional Data Access / Ethics Committee (contact via XXX) for researchers who meet the criteria for access to confidential data.*<br><br>*The data underlying the results presented in the study are available from (include the name of the third party*	The secondary data sets analysed during the current study are freely available upon request from the DHS website at http://dhsprogram.com/data/available-datasets.com. Searching 'Bangladesh DHS, 2014' in the DHS website will provide the survey data set.

and contact information or URL).<br>• This text is appropriate if the data are owned by a third party and authors do not have permission to share the data.<br><br>* typeset	
Additional data availability information:	

Powered by Editorial Manager® and ProduXion Manager® from Aries Systems Corporation

Manuscript

Click here to access/download;Manuscript;Manuscript_Stunting_vancouvre.

Sociodemographic Risk Factors of Under-Five Stunting in Bangladesh: Assessing the Role of Interactions Using a Machine Learning Method

Mohaimen Mansur[1*], Awan Afiaz[1], Md. Saddam Hossain[2]

1 Institute of Statistical Research and Training, University of Dhaka, Dhaka, Bangladesh

2 Population Council, Dhaka, Bangladesh

* Corresponding author

E-mail: mmansur@isrt.ac.bd

Abstract

Childhood stunting has been a persistent problem in Bangladesh with decades of interventions failing to reach the intended targets. Existing empirical studies investigating the problem in a socioeconomic context largely ignore the complex interplay of potential determinants that may be crucial for an intricate understanding of child malnutrition. This paper aims to demonstrate the importance of studying interactions among various sociodemographic risk factors of stunting with the help of an interpretable machine learning method. Data used for the analyses are extracted from the Bangladesh Demographic and Health Survey (BDHS) 2014 and pertain to a sample of 6,170 under-5 children. A classification tree was first constructed to identify important interaction-based rules that characterize children with different profiles of risk for stunting. Then binary logistic regression models were fitted to measure the importance of these interactions along with the individual risk factors. Results revealed that risk classifications defined by the interplay of multiple sociodemographic factors showed more extreme odds ratios (OR) of stunting than single factor ORs. The findings endorse the need for tailored-intervention programs for children based on their distinct risk profiles and sociodemographic characteristics. Such targeted efforts could prove to be cost-effective and more efficient in achieving the Health Nutrition and Population Sector Program (HNPSP) objective of limiting the prevalence of stunting in children within 25% by the year 2022 and aid in attaining the Sustainable Development Goal 2.2 of ending all forms of child malnutrition in Bangladesh.

Keywords: child stunting, malnutrition, machine learning, classification tree, public health, intervention, policy.

1 Introduction

Despite many socioeconomic challenges, Bangladesh achieved remarkable successes in many areas of public health during the last two decades which include a significant reduction in child and maternal mortality [1]. What remained as a lasting health problem for the country, however, is high childhood malnutrition. A key measure of this nutrition deficiency is low height-for-age, commonly known as stunting, which reflects the aggregate repercussions of undernutrition in children since birth or even prenatal stages, indicating long-term restriction on a child's growth potential [2]. The current situation of childhood stunting in Bangladesh is alarming. According to the latest round of Bangladesh Demographic and Health Survey conducted in 2017, almost one-third of the under-five children fail to reach a standard height for their ages [3]. This figure is one of the highest in the world outside Sub-Saharan Africa, and largely overshadows the 12 percentage-point decline in the prevalence of stunting that has been achieved by Bangladesh over the last decade [3].

Furthermore, the chronological drop in the prevalence of stunting from 51% in 2004 to 31% in 2017 translates to an average annual rate of reduction (AARR) of 3.4% which falls short of the global AARR of 3.9% required to attain World Health Organization's target to reduce by 40% the global prevalence of stunting by 2025 [4]. At the current reduction rate, Bangladesh is also unlikely to achieve its government's 4th Health Nutrition and Population Sector Program (HNPSP) objective of limiting the prevalence of stunting in children within 25% by the year 2022 [3]. This steady but slow rate of reduction in childhood stunting, coupled with a high prevalence, presents a persistent threat to the national health status of children in Bangladesh. It is now well-known that in addition to fatal consequences like child mortality low age-for-heigh[illegible] also has long-term health effects including chronic illness, disabilities, and cognitive impairment [5, 6]. Moreover, stunted children are likely to be physically, intellectually, and economically less productive in adulthood than well-nourished children [7, 8].

The long-standing problem of childhood stunting in Bangladesh has duly attracted substantial attention in academic research. A major interest of this vast literature lies in identifying the sociodemographic determinants of stunting, a key step for designing effective socioeconomic policy interventions targeted to reduce this public health problem. Risk factors that commonly

appeared in many empirical studies include children's age, gender, birth order, underweight mothers, low levels of parent's education, and poor socioeconomic status, among others. Some of the recent studies are [9, 10, 11, 12, 13, 14, 15, 16, 17, 18, 19, 20, 21]. Rarely addressed in these existing papers, however, is the possible complex interaction among different sociodemographic features of children. It is often the intricate interplay of multiple risk factors rather than individual factors that characterize potential risks of young-age undernourishment. For example, while nourishment is generally low in children whose families are poor or children whose mothers are not educated, it is probably even lower in children who are exposed to both these risk factors. From a policy perspective, this would mean that improving both education and economic conditions of the most vulnerable group may yield a faster and more efficient reduction in stunting. The main argument of this paper stems from the fact that studies that ignore these possible interactions are likely to provide an inadequate understanding of the sociodemographic disparities driving childhood stunting and therefore, are likely to offer limited policy values. This study, therefore, aims to justify the merits of incorporating such interactions among potential driving factors of childhood stunting in Bangladesh by using data from a nationally representative survey.

One of the possible reasons why interactions between variables did not feature much in the empirical public health research including nutrition studies is their predominant use of regression models, either in the form of linear or logistic. These statistical models are not best suited to capture and analyze interactions, particularly when the important ones are not known in advance. Typically, the number of possible interactions increases drastically with the number of variables incorporated in a regression model, leading to the curse of dimensionality [22]. This eventually results in the over-parametrization of models and subsequent difficulties in estimation.

In this paper, we go against the convention and exploit the classification tree, a method borrowed from the machine learning arena, to identify important interactions capable of apprehending the complex interplay between the sociodemographic variables discussed in the literature. Classification trees scale up well to large datasets and use combinations of explanatory variables to stratify data into various risk groups. Unlike parametric regression models, this nonparametric method offers a more flexible data-driven approach to fit complex data and also

offers an easy and intuitive interpretation of results [23].

While classification trees are being increasingly used in many areas of public health research, its applications in the field of childhood nutrition are still few [24, 25, 26]. To our knowledge, only one paper [27] used classfication trees to discover risk factors of childhood stunting in the context of Bangladesh. While the paper uses a rich set of biological, socioeconomic, and environmental information on children under two years of age, the sample data is limited only to low-income participants from a small district (smaller regions under divisions) of rural Bangladesh. Consequently, findings from the paper are not directly generalizable to the relevant child population in Bangladesh which very likely exhibits wider sociodemographic and spatial variations. In contrast, our study utilizes data from a population-based nationally-representative survey. A fundamental difference of this paper with these existing studies is that in addition to identifying interactions among sociodemographic determinants of stunting it further measures the importance and statistical significance of these interactions through applications of classical regression models.

The current analyses aim to make important methodological contributions by utilizing the classification tree to identify groups of children with various risk profiles for stunting. The importance of such risk stratification is further verified through the use of widely accepted statistical models. Importantly, this risk profiling is characterized by the interaction of multiple sociodemographic features of children allowing the possibility for targeted interventions based on their respective vulnerabilities. Considering the fact that Bangladesh is observing an undesirably slow reduction in childhood stunting, such need-specific tailored policies can speed up the progress towards achieving the Goal 2.2 of the Sustainable Development Goals (SDGs) that aims to end all forms of malnutrition including stunting in children under 5 years of age by the year 2030 [28].

2 Materials and Methods

2.1 Data overview

This study used data from the Bangladesh Demographic and Health Survey (BDHS) 2014. This is a nationally representative cross-sectional survey conducted in Bangladesh in collaboration with the Demographic and Health Survey (DHS) and is operated by Measure DHS+ under the authority of the National Institute of Population Research and Training (NIPORT). The survey sampling frame was based on the 2011 Bangladesh population census, and a two-stage stratified cluster sampling technique was employed in the survey. In the first stage, 600 enumeration areas (EAs), a geographic area consisting of 113 households on average, were independently selected with probability proportional size (PPS) method from all 20 sampling strata. Then using an equal probability systematic selection in the second stage, 30 households were selected from each cluster (EA). The survey resulted in 17,886 completed interviews with ever-married women between the ages of 15 to 49. Further details of the sampling procedure can be found in [29].

The survey also collected measurements on height and weight from eligible women of reproductive ages and children under the age of 5 years. Complete and credible anthropometric and age data on 7,318 children were collected in the process [29]. After excluding the temporary (de facto) residents, children of twin births and any cases with missing values in the variables, the final sample size for this study was 6,170.

2.2 Response variable

Anthropometric measurements of height and weight were used to assess the nutrition status of Bangladeshi children under 5 years of age. The BDHS 2014 data constructed several indices from these measurements according to the WHO Child Growth Standards, such as stunting, wasting, and underweight, that takes into account the variations in ethnicity, social and economic influences, and feeding practices worldwide [30]. These indices provide important information about a child's growth and body composition and act as key indicators in order to assess their nutritional status.

This study concerns only the height-for-age or stunting index which reflects the cumulative

effect of constant malnourishment as well as recurrent and chronic illnesses resulting in lack of growth among children compared to their respective ages. Two standard deviations below the median (-2 SD) of the WHO reference population in terms of height-for-age indicates that the child is short for his or her age, i.e. stunted [30]. This variable was pre-calculated according to the definition provided by WHO in the BDHS 2014 child dataset.

2.3 Independent variables

Based on the literature review and pre-analysis, the current study explored the variables that commonly appeared in most nutrition related studies on Bangladesh. At the child level, these included the age of children in months (0-59 months), sex (male, female), as well as the birth order (first born, second born, and third born or above) of the child and the decision-maker regarding child's healthcare needs (mother only, both parents, and husband and/or someone else). Independent variables related to the mother such as mother's age at the birth of her first child (in years), number of living children (one, two, and three or more children), body mass index (BMI) level (less than 18.5, between 18.5 and 25, and greater than 25), education level (no education, primary, secondary, higher secondary or above), current employment status (currently working, not working) and mother's age difference to the father (in years) along with father's education level (no education, primary, secondary, higher secondary or above) were analyzed.

The sociodemographic variables, related to the child's household characteristics, were also explored for association with stunting in children under 5 years of age, which included household's wealth index (poorest, poorer, middle, richer, richest), area of residence (urban, rural) and administrative division (Dhaka, Barishal, Chattogram, Khulna, Rajshahi, Rangpur, Sylhet). The wealth index quintiles were pre-calculated using principal component analysis on household assets [31] and were readily available in the dataset.

2.4 Methodology

The analyses of the paper were carried out in two steps using a machine learning approach and a classical regression model in succession. In the first step of the analysis, a classification tree was fit to the data to identify possible interactions among predictors of childhood stunting. In the second step, the interactions identified by the trees are incorporated in a logistic

regression framework in addition to individual explanatory variables, and their impact on the likelihood of stunting measured. This two-step method can be thought to be a simpler version of the *Rulefit* approach proposed in [32] where an ensemble of trees rather than a single tree is used to identify important interaction rules which are then included in a penalized regression model.

A classification tree divides the sample data into small subgroups based on simple rules involving the predictors. The rules originate from step-wise binary splits of the predictors where the most important predictor at each step (referred to as a node) is selected for splitting. The tree is produced with an aim to achieving minimum error in classification (e.g., whether a child is stunted or not) for subgroups produced at each node. A classification tree is nonparametric in nature and holds a number of advantages over parametric regression type models including minimal distributional assumptions and easier interpretation. The most important advantage, however, is the tree's inherent capability of capturing complex, such as non-linear, interactions of variables in high dimensional settings [23]. The standard classification tree techniques such as CART [33] are often criticized for biased selection of variables which have many possible splits and missing values. These problems have been rectified in a conditional inference tree framework proposed in [34] that has been adopted in the current analyses. The depth of the tree was fixed to four layers to keep a balance between capturing adequate complexity and avoiding too low (less than 50) sample size per node.

While trees offer flexible approaches to reveal interactions among explanatory variables from the sample data, it does not provide measurable sizes of effects of these interactions on the response (childhood stunting) or their statistical significance. To achieve these, interactions/rules were incorporated in a logistic regression setting as additional predictors. Rules yielding either a stunting prevalence of greater than 40% or less than 20% were considered suggestive of significantly high and low prevalence, respectively, and considered for entering into the regression model. These thresholds for rule selection were constructed based around the national prevalence of under-five stunting of 36% in Bangladesh [29].

Each selected rule is then converted to a binary variable taking a value of 1 when the defining criteria for that particular rule are met, and a value of 0, otherwise. Each of these binary

variables is, thus, defined by unique interactions of several sociodemographic features of children. Next, two logistic regression models were estimated and compared. In the first model, only the initially selected independent variables were fitted to the stunting outcome (stunted, not stunted) and no interaction rule was considered. But in the second model, binary variables from selected rules were included along with the individual variables. Survey weights are duly applied in both the classification and logistic regression for making the sample results representative of the population of interest. Findings from regression models are further adjusted for the complexity of the survey design to capture cluster- and strata-wise variations and appropriately compute the standard errors and p-values for regression estimates.

Finally, classification accuracies of the two logistic regression models are systematically evaluated for both in-sample goodness of fit and out-of-sample predictive performance. Comparisons are made by computing standard criteria such as accuracy, sensitivity, specificity and the area under the Receiver Operating Characteristic Curve (ROC). The first three criteria are calculated based on a probability threshold of 0.5. To obtain the area under the curve (AUC) for the out-of-sample ROCs, a 10-fold cross-validation approach was undertaken and the mean AUC values were reported in the findings.

To ensure the reproducibility in scientific research in cases of new discoveries, the present study followed the recommendation by [35] in identifying the significant interaction rules. That is, the proposed interaction terms will be considered significant if the corresponding p-values were less than 0.005 in the second model. All analyses were performed in R *(version 3.6.0)*. The binary logistic regression models were fitted using the *"survey"* package *(version 4.0)* and the classification tree was constructed using the *"partykit"* package *(version 1.2-7)*.

3 Results

3.1 Descriptive statistics

Table 1 presents the distribution of stunting in under-five children by their sociodemographic characteristics considered in the study. Although the age of the children was considered as a continuous numeric variable in later analysis, it was converted into a categorical variable for exploratory reasons in this table. The overall stunting prevalence was 36.5% in the study

data. The results reveal that children aged two years or older were more stunted (41.8%) than younger children (28.3%). Children of higher birth order were also stunted in higher proportions (42.7%) than the first or second-born children.

Table 1: Distribution of stunting by sociodemographic variables (categorical and continuous) among children aged 0-59 months

**Sociodemographic variables**	**Group**	**N (%) of study participants**	**N (%) of Stunted**
**Age of child (in months)**	0 – 23 months	2432 (39.4)	689 (28.3)
	24 – 59 months	3738 (60.6)	1563 (41.8)
**Sex of child**	Male	3167 (51.3)	1175 (37.1)
	Female	3003 (48.7)	1077 (35.9)
**Child's birth order**	First born	2260 (36.6)	730 (32.3)
	Second born	1885 (30.6)	657 (34.9)
	Third born or above	2025 (32.8)	865 (42.7)
**Decision maker regarding child's healthcare**	Mother only	948 (15.4)	319 (33.6)
	Both parents	3493 (56.6)	1252 (35.8)
	Husband or Somebody else	1729 (28.0)	681 (39.4)
**Age of mother at first birth (mean [SD])**		18.17 [3.23]	
**No of children alive to mother**	One child	2060 (33.4)	636 (30.9)
	Two children	2122 (34.4)	756 (35.6)
	Three or more	1988 (32.2)	860 (43.3)
**Mother's BMI**	Less than 18.5	1387 (22.5)	633 (45.6)
	18.5 – 25.0	3619 (58.7)	1330 (36.8)

Table 1 – *Continued*

Sociodemographic variables	Group	N (%) of study participants	N (%) of Stunted
	Greater than 25.0	1164 (18.9)	289 (24.8)
**Mother's education level**	No Education	981 (15.9)	491 (50.1)
	Primary	1727 (28.0)	770 (44.6)
	Secondary	2835 (45.9)	866 (30.5)
	Higher	627 (10.2)	125 (19.9)
**Father's education level**	No education	1564 (25.3)	773 (49.4)
	Primary	1881 (35.5)	767 (40.8)
	Secondary	1854 (30.0)	541 (29.2)
	Higher	871 (14.2)	171 (19.6)
**Mother's employment status**	Currently working	1593 (25.8)	635 (39.9)
	Not working	4577 (74.2)	1617 (35.3)
**Mother's age difference to father (median)**		8.0	
**Household wealth index**	Poorest	1359 (22.0)	697 (51.3)
	Poorer	1159 (18.8)	476 (41.1)
	Middle	1202 (19.5)	450 (37.4)
	Richer	1256 (20.4)	394 (31.4)
	Richest	1194 (19.3)	235 (19.7)
**Area of residence**	Rural	4229 (68.5)	1623 (38.4)
	Urban	1941 (31.5)	629 (32.4)
**Administrative division**	Dhaka	1081 (17.5)	366 (33.9)

Table 1 – *Continued*

**Sociodemographic variables**	**Group**	**N (%) of study participants**	**N (%) of Stunted**
	Barishal	725 (11.8)	278 (38.3)
	Chattogram	1156 (18.7)	431 (37.3)
	Khulna	687 (11.1)	190 (27.7)
	Rajshahi	756 (12.2)	227 (30.0)
	Rangpur	770 (12.5)	272 (35.3)
	Sylhet	995 (16.1)	488 (49.0)
**Total sample size**	N	6170	2252 (36.5)

The mean age of mothers at their first birth was 18.2 years with a standard deviation of 3.23 years. Fathers were mostly older than mothers with a median age difference of 8 years. Children belonging to mothers with three or more living children were more stunted (43.3%) than those households where they were the only child (30.9%) or had only one sibling (35.6%). The prevalence of stunting is also lower (33.6%) in cases where the mother was the sole decision-maker regarding the child's healthcare needs in comparison to cases where decisions are made by the husband or another member of the family (39.4%). The prevalence of stunting for children belonging to underweight mothers was more than 20% higher than that in children born to mothers who were overweight (24.8%). Reduced percentages of stunting were observed for higher levels of educational attainment of both mother and father. Working mothers had a higher percentage of stunted children (39.9%). The proportion of stunted children was also high at the lowest wealth quintile (51.3%) but decreased with higher levels of wealth. Percentage stunted in rural households (38.4%) was higher than the national average of 36% while that in urban households was below (32.4%). The prevalence of stunting was the highest in the Sylhet division (49%) and the lowest in the Khulna division (27.7%).

3.2 Classification tree

The classification tree in **Figure 1** shows the interplay of important sociodemographic factors behind stunting in children under five years of age. The wealth reveals itself to be the most important predictor being placed at the top of the tree. Children belonging to middle or

Figure 1: Classification tree for stunting in under-five children in Bangladesh

The value of n at each final node gives the node sample size and the black fill in the box below indicates proportion of stunted children under the node. The p-value at each node corresponds to a unified permutation test of hypothesis indicating independence between the response and predictors [34].

lower categories of wealth statuses are placed under the left branch. The age of a child is the next most important variable in this branch showing children younger than 14 months to be less vulnerable to stunting. Other noteworthy predictors on this side of the tree were father's education, administrative division, and the birth order of the child. In this case, children older than 14 months with lowly educated fathers living in Dhaka, Barishal, Chattogram, and Sylhet divisions (node 10) seemed to be at the highest risk of being stunted.

On the right-hand branch originated from the root, for wealthier households, mother's education, and then their body mass index (BMI) as well as the age of a child were notable predictors of stunting. Here, mothers having only lower levels of education with children older than 9 months and a lower BMI (node 21) demonstrate increased risk of child stunting. From the classification tree, we chose six interaction rules defined by six nodes (6, 10, 11, 13, 21, and 26, respectively) to be important for our analysis. The previously stated national prevalence of stunting of 36% has been utilized to determine the thresholds for these rule selections. A node was considered to be of significant importance if it showed the prevalence of stunting to be higher than 40% (high risk) or less than 20% (low risk). However, we excluded nodes 18 and 19 from further consideration on account of having considerably low sample sizes. Selected six nodes were then transformed into binary variables based on whether the observations satisfied all four classification criteria under each node, displayed in **Table 2**, and are named as "Rule-6, 10, 11, 13, 21, and 26" respectively.

3.3 Logistic regression models

Results from the logistic regressions are presented in **Table 3**. The first model which included only the sociodemographic variables in the study, exhibits that stunting was positively associated with the age of a child (OR 1.02 with 95% CI: 1.015, 1.024), corroborating the findings from the descriptive statistics. The likelihood of stunting reduced by nearly 20% when the mother was the sole decision-maker of the child's healthcare needs (OR 0.81 with 95% CI: 0.66, 0.99) or when both parents jointly made such decisions (OR 0.83 with 95% CI: 0.71,

Table 2: Interaction rules extracted from the Classification tree and associated stunting

Rule ID	Rule combinations	Node frequency	Stunting prevalence
**Rule 6**	Wealth index = Poorest, Poorer, Middle & Age of child <= 14 & Age of child <= 9 & Birth order = Second born, Third born or above	357	11.50%
**Rule 10**	Wealth index = Poorest, Poorer, Middle & Age of child > 14 & Father's education level = No education, Primary & Division= Dhaka, Barisal, Chittagong, Sylhet	1277	60.50%
**Rule 11**	Wealth index = Poorest, Poorer, Middle & Age of child > 14 & Father's education level = No education, Primary & Division= Khulna, Rajshahi, Rangpur	800	43.90%
**Rule 13**	Wealth index = Poorest, Poorer, Middle & Age of child > 14 & Father's education level = Secondary, Higher & Wealth index = Poorest, Poorer	372	44.40%
**Rule 21**	Wealth index = Richer, Richest & Mother's education level = No education, Primary & Age of child > 9 & Mother's BMI = Less than 18.5, Between 18.5 to 25	371	48.20%
**Rule 26**	Wealth index = Richer, Richest & Mother's education level = Secondary, Higher & Mother's BMI = Between 18.5 to 25 , Greater than 25 & Wealth index = Richest	942	16.70%

0.97) as opposed to the father or somebody else being in charge of such decision-making. The odds of stunting reduced by 39% (p-value < .001) for mother's with a BMI greater than 25, in comparison to mothers with a BMI score between 18.5 and 25. Father's education also played a part with both secondary (OR 0.73 with 95% CI: 0.58, 0.92) and higher (OR 0.54 with 95% CI: 0.39, 0.75) categories showing a significantly lower likelihood of stunting compared to fathers with no education.

Table 3: Odds ratios and associated inference from logistic regression models

**Sociodemographic factors**	**Model 1: No interaction OR (95% CI)**	**p-value**	**Model 2: With interaction rules OR (95% CI)**	**p-value**
**Age of child (in months)**	1.02 (1.015, 1.024)	<.001	1.00 (1.00, 1.01)	0.094
**Sex of child (ref: Male)**				
Female	0.97 (0.84, 1.12)	0.666	0.95 (0.83, 1.09)	0.489
**Child's birth order (ref: First born)**				
Second born	1.08 (0.81, 1.45)	0.597	1.06 (0.78, 1.44)	0.698
Third born or above	1.02 (0.64, 1.61)	0.941	1.11 (0.70, 1.75)	0.663
**Decision maker regarding child's healthcare (ref: Husband or somebody else)**				
Mother only	0.81 (0.66, 0.99)	0.04	0.82 (0.66, 1.01)	0.063
Both parents	0.83 (0.71, 0.97)	0.019	0.83 (0.71, 0.97)	0.021
**Age of mother at first birth**	0.98 (0.95, 1.01)	0.177	0.98 (0.95, 1.01)	0.236
**No of children alive to mother (ref: One child)**				
Two children	1.18 (0.87, 1.58)	0.288	1.34 (0.98, 1.82)	0.067
Three or more children	1.09 (0.68, 1.75)	0.722	1.14 (0.71, 1.81)	0.591
**Mother's BMI (ref: Between 18.5 to 25.0)**				
Less than 18.5	1.15 (0.91, 1.45)	0.237	1.11 (0.88, 1.44)	0.367
Greater than 25.0	0.61 (0.50, 0.75)	<.001	0.68 (0.56, 0.84)	<.001
**Mother's education level (ref: No education)**				
Primary	1.09 (0.81, 1.46)	0.565	1.05 (0.78, 1.41)	0.76
Secondary	0.89 (0.66, 1.19)	0.43	1.03 (0.77, 1.38)	0.847
Higher	0.99 (0.64. 1.54)	0.973	1.18 (0.77, 1.83)	0.449
**Father's education level (ref: No education)**				
Primary	0.88 (0.74, 1.04)	0.124	0.90 (0.75, 1.08)	0.256

Table 3 – *Continued*

Sociodemographic factors	Model 1: No interaction OR (95% CI)	p-value	Model 2: With interaction rules OR (95% CI)	p-value
Secondary	0.73 (0.58, 0.92)	0.007	0.92 (0.71, 1.20)	0.545
Higher	0.54 (0.39, 0.75)	<.001	0.72 (0.51, 1.03)	0.07
**Mother's employment status (ref: Not working)**				
Currently working	1.21 (1.01, 1.44)	0.034	1.21 (1.02, 1.44)	0.032
**Mother's age difference to father**	0.99 (0.98, 1.00)	0.084	0.99 (0.97, 1.00)	0.069
**Wealth Index (ref: Poorest)**				
Poorer	0.86 (0.71, 1.04)	0.117	0.83 (0.68, 1.01)	0.061
Middle	0.72 (0.59, 0.89)	0.002	0.77 (0.62, 0.95)	0.017
Richer	0.58 (0.45, 0.75)	<.001	0.65 (0.49, 0.87)	0.004
Richest	0.36 (0.26, 0.49)	<.001	0.56 (0.35, 0.90)	0.016
**Area of Residence (ref: Rural)**				
Urban	1.29 (1.04, 1.60)	0.02	1.28 (1.03, 1.59)	0.028
**Administrative division (ref: Dhaka)**				
Barishal	1.11 (0.85, 1.44)	0.438	1.11 (0.85, 1.46)	0.433
Chattogram	1.20 (0.97, 1.47)	0.092	1.20 (0.97, 1.48)	0.099
Khulna	0.67 (0.53, 0.86)	0.002	0.80 (0.61, 1.05)	0.114
Rajshahi	0.65 (0.52, 0.82)	<.001	0.82 (0.64, 1.05)	0.122
Rangpur	0.88 (0.68, 1.14)	0.338	1.07 (0.81, 1.42)	0.632
Sylhet	1.51 (1.23, 1.86)	<.001	1.57 (1.26, 1.96)	<.001
**Rules extracted from the classification tree**				
**Rule-6 (ref: No)**				
Yes			0.22 (0.15, 0.33)	<.001
**Rule-10 (ref: No)**				
Yes			2.52 (1.85, 3.44)	<.001
**Rule-11 (ref: No)**				
Yes			1.57 (1.09, 2.07)	0.001

Table 3 – *Continued*

**Sociodemographic factors**	**Model 1: No interaction**		**Model 2: With interaction rules**	
	**OR (95% CI)**	**p-value**	**OR (95% CI)**	**p-value**
**Rule-13 (ref: No)**				
Yes			1.64 (1.15, 2.33)	0.006
**Rule-21 (ref: No)**				
Yes			2.01 (1.41, 2.86)	<.001
**Rule-26 (ref: No)**				
Yes			0.65 (0.43, 0.99)	0.047

Interestingly, having a working mother increased the odds of stunting by 21% (p-value: 0.034) compared to mothers who were not employed at the time of the survey. Children from middle, rich and, the richest households were 28%, 42%, and 64% less at risk of stunting compared to children from poorest households (p-value <.005). Furthermore, children from urban areas were significantly more likely (OR 1.29 with 95% CI: 1.04, 1.60) to be stunted than children in rural areas. Children living in Sylhet division had 51% higher risk of being stunted (p-value <.001) compared to Dhaka, whereas the burden of risk was more than 30% lower for children living in Khulna (OR 0.67 with 95% CI: 0.53, 0.86) and Rajshahi (OR 0.65 with 95% CI: 0.52, 0.82).

The second model, where the interaction rules extracted from the classification tree were included along with the sociodemographic variables, showed somewhat different results. Age of children, the mother being the sole decision-maker of child's healthcare, father's education level and administrative divisions (barring Sylhet) were no longer associated with stunting individually at 5% level of significance. However, the rest of the significant associations remained as they were with minor changes in the odds of either increasing or decreasing the likelihood of stunting.

Following the suggestion by [35], the interaction rules 6, 10, 11, and 21 were significantly associated with stunting at 0.5% level of significance. The children corresponding to rule-6, indicating those who live in the poorest, poorer or middle-wealth households, who are younger

than 9 months and who rank second or higher in the birth order, were 78% less at risk of stunting than those who do not belong to this rule. According to rule-10, odds of stunting in children of lower wealth categories who are more than 14 months old, have primary or uneducated fathers and live in any one of Dhaka, Barisal, Chittagong, Sylhet divisions was 2.5 times the odds in the children who do not fall into these categories. In contrast, according to rule-11, children with same wealth, age and paternal education levels as in rule-10, exhibit an odds ratio of only 1.54 when they resided in any one of the other three divisions - Khulna, Rajshahi, or Rangpur. It is also worth noting that, children aged greater than 14 months who belonged to the poorest or poorer household but have secondary or higher educated fathers were also at 64% higher odds of stunting (rule-13 with p-value 0.006). Finally, based on rule-21, children aged greater than 9 months who belonged to the richer or richest households with mothers having little or no formal education and a BMI of 25 or less were twice as likely to be stunted as children who have different other combinations of risk factors.

3.4 Model validation

The predictive performances of both logistic regression models were evaluated to assess whether the addition of decision tree rules as interactions in the second model played any part in improving the results. For out of sample predictions a 10-fold cross-validation procedure was followed. **Table 4** shows that, when fitting in-sample data is concerned, including the decision tree rules in the model yielded a more than 18% increase in the sensitivity at the cost of a marginal drop in specificity (2.35%). Similarly, in the out-of-sample predictions, sensitivity improved by 22% with a slight drop in specificity. In both scenarios, larger areas under the ROC curve (AUC) further confirmed the benefit from adding the interaction-induced rules in the model.

4 Discussion

The present analysis assessed the role of interactions between important sociodemographic factors, commonly identified in the literature, in explaining childhood stunting. The findings from the two-step analysis provided several interesting perspectives. In the exploratory part of the analysis, the classification tree identified the specific groups of under-five children with various levels of risk for stunting. Essentially, each of these different risk profiles was charac-

Table 4: Comparison of model performance

**Performance type**	**Sensitivity**	**Specificity**	**Accuracy**	**AUC**
**In-sample (full sample fit)**				
Model 1: Without interaction	0.366	0.847	0.671	0.692
Model 2: With interaction	0.440	0.832	0.689	0.715
**Out-of-sample (10-fold cross validation)**				
Model 1: Without interaction	0.360	0.850	0.674	0.694
Model 2: With interaction	0.436	0.824	0.684	0.712

Sensitivity, specificity and accuracy are computed based on a probability threshold of 0.5. Results for out-of-sample performances are averages across 10-fold cross-validations. AUC is the area under the Receiver Operating Characteristic (ROC) curve.

terized by a unique combination of several predictively important sociodemographic features and provided noteworthy insights into the phenomenon of childhood stunting. These insights stemming from the complex interplay of multiple factors usually go unnoticed when looking at the pertinent factors individually.

For example, the distribution of stunted children by household wealth quintile reported in **Table 1** shows that the percentage of stunted children in households belonging to the richer and richest wealth statuses were 31.4% and 19.7%, respectively. However, node 21 of the classification tree in **Figure 1** (Rule 21 in Table 2) revealed that a sub-group of children from these wealth classes who were above 9 months of age and born to illiterate or lowly educated (primary education) mothers having low to moderate BMI (BMI < 25), have a much higher (48%) prevalence of stunting. Note that, the use of an interpretable machine learning method has divulged insights with more depth than what would normally be achieved.

Similarly, vulnerable groups with analogous risk profiles could be characterized by very different combinations of risk factors with the help of classification trees. Evidently, children under node 11 of the classification tree (Rule 11 in Table 2) had only 4% lower risk of stunting than those in the aforementioned node 21, but possess noticeably dissimilar features concerning household wealth and parental education.

Findings from the classification tree corroborate what we already know about childhood stunting in developing countries including Bangladesh while providing us with fresh perspec-

tives. Sociodemographic variables that appeared in the tree have been largely identified as significant predictors of child growth in the literature as well as in the factor-only logistic regression model of this study (Model 1 of Table 3). The prevalence of adverse conditions and food insecurity in underprivileged households is well-documented and so is the association between poverty and stunting [36, 20]. Mother's educational attainment is an important factor for a child's nourishment [12, 37] as this corresponds to women's knowledge regarding mothering a child and their subsequent care-taking. Paternal education has been also found to be relevant [38, 17] as it is linked to increased earning and health awareness, particularly in low- and middle-income households, and complements maternal education in many cases [39]. Furthermore, mother's own physical attributes and nourishment, indicated by mother's BMI, is a significant aspect affecting the child's health as well [40, 41]. Higher age and higher birth order are also frequently linked to childhood stunting [42, 43]. Individually considered, all of these are undoubtedly important factors. However, the interplay between these factors offered a deeper understanding of the sociodemographic effect on child malnutrition which is likely to be crucial in designing targeted intervention strategies.

In the confirmatory part of the analysis, selected groups of children with low and high risks of stunting, as identified by the tree, were incorporated into a logistic regression model (Model 2 in Table 3) together with the individual risk factors and were investigated for their possible associations with stunting. Here, all but one association were statistically significant at least at 1% level of significance. More importantly, the strength of associations of these interaction-induced risk groups with stunting, measured by odds ratio (OR), were predominantly higher (for positive associations) or lower (for negative associations) than those for individual risk factors.

As an example, the highest odds ratio for an individual variable was 1.57, observed for the Sylhet division, implying that living in this division increased the likelihood of stunting by 57% compared to the residents in the Dhaka division. In contrast, the odds ratios for positively associated rules are in the range of 1.57 to 2.52, the highest corresponding to rule 10 reported in Table 2 and implying that the odds of stunting for children living in certain divisions including Sylhet and possessing specific risk features was 2.5 times the odds for children having other characteristics. Similarly, among the individual predictors, children belonging to the richest

household wealth quintile were associated with the lowest odds ratio (OR) of 0.56, while the OR for a negatively associated rule was as low as 0.22 (rule 6 in Table 2).

The combination of results from the classification tree and the logistic regression may bear valuable implications on research and policies related to stunting in under-five children. Identification of vulnerable groups and specific interactions of multiple sociodemographic factors that define these groups can aid in expanding the understanding of different aspects of stunting. Policymakers working towards the reduction of stunting may utilize this intricate knowledge in designing risk-specific tailored interventions by targeting the characterizing features.

One policy recommendation based on the findings of this paper will pertain to targeting relatively poor and lowly educated families living in Sylhet, Chattogram, Barishal, or Dhaka divisions who have the highest risk of stunting and attempt to increase post-primary education and employment among them. These can be achieved by making higher education free or cheaper in these communities and by creating low-capital income-generating opportunities. The second set of intervention programs could be formulated by focusing on encouraging higher female education, with a particular emphasis on mother and child's health literacy, as well as ensuring proper nutrition among mothers in relatively richer households. Such small-scale targeted interventions can be quicker and more effective, but less expensive in achieving the desired reduction in stunting as opposed to a nation-wide implementation of a more general policy such as making higher female education free of cost in the entire country. Importance of such tailored interventions are emphasized in disciplines like innovation studies [44] and nursing care [45].

While the focus of the paper is the evaluation of interaction among sociodemographic characteristics of children as determinants of stunting, a by-product of the findings of the analysis is that children whose healthcare decisions are made either by the mother alone or jointly by both parents have significantly lower odds of stunting than children whose decisions are made solely by the father or someone else. This could also be a key learning in the understanding of the sociodemographic mechanism behind childhood stunting in Bangladesh and is in line with findings in the literature [46]. Intervention programs aimed at increasing the practice of granting mothers' autonomy in child's healthcare decision-making or encouraging mothers' in-

clusion in such decisions could go a long way in achieving the goal of alleviating this problem. Furthermore, interventions programs that already include educating mothers regarding child's health and nourishment could also be positively impacted by the inclusion of mothers in such decision-making practices.

The present study has a few limitations. Firstly, the secondary data was obtained from a cross-sectional survey as opposed to a longitudinal study and so no causal relationship between the factors and the outcome could be established. Secondly, the selection of explanatory variables is limited to those that generally appeared significant in major nutritional studies rather than all possible inclusions from an exhaustive search of the literature. Thirdly, the tree depth was limited to four layers for avoiding too low sample size per node and two nodes were excluded from analysis as a result even after exceeding the pre-specified thresholds.

5 Conclusion

High prevalence of childhood stunting and low rate of reduction per year imply that Bangladesh is falling behind in meeting national and international targets for reducing early-age undernutrition. This paper emphasizes the importance of studying the possible interplay of different sociodemographic determinants of child stunting, largely missing from existing literature, for a deeper insight into the problem. With that aim, analyses adopted in the paper exploit a classification tree to uncover complex interactions from data and incorporate them in a logistic regression model. Significantly high and low magnitudes of odds ratios justified the importance of interactions above the individual factors in explaining childhood stunting in Bangladesh. Superior fit to data and better predictive performance of the interaction-added model over the factors-only model further validated the significance of considering these interactions in the analysis and endorsed the need for an in-depth understanding of the inherent mechanisms between the key factors.

Despite some limitations, the strength of the study lies in the novel approach of combining an interpretable machine learning method with a classical statistical model to identify complex but significant interactions between the sociodemographic factors behind child stunting in Bangladesh and associated risk-groups. When public health interventions are concerned,

findings of this paper emphasize importance of targeting multiple risk factors that interact with each other in defining groups of children most vulnerable to stunting. This may speed-up slow reduction of childhood stunting in Bangladesh and prove worthwhile in making meaningful strides towards achieving the Government's 4th Health Nutrition and Population Sector Program (HPNSP) objective of reducing the prevalence of stunting to 25% by 2022 [3]. Importantly, the complementary methods demonstrated in this study can be applied to gain deeper socio-economic understanding of many other public health questions related to, for example, early childhood development, women's healthcare decision making, hygiene practices etc.

Acknowledgments

The authors would like to acknowledge MEASURE DHS (Demographic and Health Surveys), National Institute for Population Research and Training (NIPORT) and USAID/Bangladesh, who allowed researchers to access the survey data for free.

References

[1] World Health Organization, et al. Success factors for women's and children's health: Bangladesh. 2015;.

[2] World Health Organization. Nutrition Landscape Information System (NLIS) country profile indicators: interpretation guide. 2010;.

[3] NIPORT. Bangladesh Demographic and Health Survey 2017-18: Key Indicators. Dhaka, Bangladesh, and Rockville, Maryland, USA: NIPORT, and ICF. 2019;.

[4] De Onis M, Dewey KG, Borghi E, Onyango AW, Blössner M, Daelmans B, et al. The W orld H ealth O rganization's global target for reducing childhood stunting by 2025: rationale and proposed actions. Maternal & child nutrition. 2013;9:6–26.

[5] Dewey KG, Begum K. Long-term consequences of stunting in early life. Maternal & child nutrition. 2011;7:5–18.

[6] Prendergast AJ, Humphrey JH. The stunting syndrome in developing countries. Paediatrics and international child health. 2014;34(4):250–265.

[7] Black RE, Victora CG, Walker SP, Bhutta ZA, Christian P, De Onis M, et al. Maternal and child undernutrition and overweight in low-income and middle-income countries. The lancet. 2013;382(9890):427–451.

[8] Stewart CP, Iannotti L, Dewey KG, Michaelsen KF, Onyango AW. Contextualising complementary feeding in a broader framework for stunting prevention. Maternal & child nutrition. 2013;9:27–45.

[9] Alom J, Quddus MA, Islam MA. Nutritional status of under-five children in Bangladesh: a multilevel analysis. Journal of biosocial science. 2012;44(5):525–535.

[10] Islam MM, Alam M, Tariquzaman M, Kabir MA, Pervin R, Begum M, et al. Predictors of the number of under-five malnourished children in Bangladesh: application of the generalized poisson regression model. BMC public health. 2013;13(1):11.

[11] Fakir AM, Khan MWR. Determinants of malnutrition among urban slum children in Bangladesh. Health economics review. 2015;5(1):22.

[12] Hasan MT, Soares Magalhaes RJ, Williams GM, Mamun AA. The role of maternal education in the 15-year trajectory of malnutrition in children under 5 years of age in B angladesh. Maternal & child nutrition. 2016;12(4):929–939.

[13] Rabbani A, Khan A, Yusuf S, Adams A. Trends and determinants of inequities in childhood stunting in Bangladesh from 1996/7 to 2014. International journal for equity in health. 2016;15(1):186.

[14] Chowdhury MRK, Rahman MS, Khan MMH, Mondal MNI, Rahman MM, Billah B. Risk factors for child malnutrition in Bangladesh: a multilevel analysis of a nationwide population-based survey. The Journal of pediatrics. 2016;172:194–201.

[15] Das S, Gulshan J. Different forms of malnutrition among under five children in Bangladesh: a cross sectional study on prevalence and determinants. BMC Nutrition. 2017;3(1):1.

[16] Talukder A. Factors associated with malnutrition among under-five children: illustration using Bangladesh demographic and health survey, 2014 data. Children. 2017;4(10):88.

[17] Hossain MB, Khan MHR. Role of parental education in reduction of prevalence of childhood undernutrition in Bangladesh. Public health nutrition. 2018;21(10):1845–1854.

[18] Huda TM, Hayes A, El Arifeen S, Dibley MJ. Social determinants of inequalities in child undernutrition in Bangladesh: A decomposition analysis. Maternal & child nutrition. 2018;14(1):e12440.

[19] Saha UR, Chattapadhayay A, Richardus JH. Trends, prevalence and determinants of childhood chronic undernutrition in regional divisions of Bangladesh: Evidence from demographic health surveys, 2011 and 2014. PloS one. 2019;14(8).

[20] Sultana P, Rahman MM, Akter J. Correlates of stunting among under-five children in Bangladesh: a multilevel approach. BMC Nutrition. 2019;5(1):41.

[21] Khanam M, Shimul SN, Sarker AR. Individual-, household-, and community-level determinants of childhood undernutrition in Bangladesh. Health services research and managerial epidemiology. 2019;6:2333392819876555.

[22] Andrews DW, Whang YJ. Additive interactive regression models: circumvention of the curse of dimensionality. Econometric Theory. 1990;6(4):466–479.

[23] James G, Witten D, Hastie T, Tibshirani R. An introduction to statistical learning. vol. 112. Springer; 2013.

[24] Pawloski LR, Kitsantas P. Classification tree analysis of stunting in Malian adolescent girls. American Journal of Human Biology: The Official Journal of the Human Biology Association. 2008;20(3):285–291.

[25] Debnath A, Bhattacharjee N. Factors associated with malnutrition among tribal children in India: a non-parametric approach. Journal of tropical pediatrics. 2014;60(3):211–215.

[26] Thangamani D, Sudha P. Identification of malnutrition with use of supervised datamining techniques–decision trees and artificial neural networks. Int J Eng Comput Sci. 2014;3(September 9):8236–8241.

[27] Svefors P, Sysoev O, Ekstrom EC, Persson LA, Arifeen SE, Naved RT, et al. Relative importance of prenatal and postnatal determinants of stunting: data mining approaches to the MINIMat cohort, Bangladesh. BMJ open. 2019;9(8):e025154.

[28] United Nations. Transforming our world: The 2030 agenda for sustainable development. General Assembley 70 session. 2015;.

[29] NIPORT. Bangladesh Demographic and Health Survey 2014. Dhaka, Bangladesh, and Rockville, Maryland, USA: National Institute of Population Research and Training (NIPORT), Mitra & Associates and ICF International. 2016;.

[30] World Health Organization (WHO). Multicentre Growth Reference Study Group 2006. WHO Child Growth Standards: Length/height-for age, weight-for-age, weight for length, weight for height: Methods and development Geneva: World Health Organization. 2006;p. 312.

[31] Rutstein SO, Johnson K. The DHS wealth index. DHS comparative reports no. 6. Calverton, MD: ORC Macro. 2004;.

[32] Friedman JH, Popescu BE, et al. Predictive learning via rule ensembles. The Annals of Applied Statistics. 2008;2(3):916–954.

[33] Breiman L, Friedman J, Stone CJ, Olshen RA. Classification and regression trees. CRC press; 1984.

[34] Hothorn T, Hornik K, Zeileis A. Unbiased recursive partitioning: A conditional inference framework. Journal of Computational and Graphical statistics. 2006;15(3):651–674.

[35] Benjamin DJ, Berger JO, Johannesson M, Nosek BA, Wagenmakers EJ, Berk R, et al. Redefine statistical significance. Nature Human Behaviour. 2018;2(1):6.

[36] Hong R, Banta JE, Betancourt JA. Relationship between household wealth inequality and chronic childhood under-nutrition in Bangladesh. International journal for equity in health. 2006;5(1):15.

[37] Vollmer S, Bommer C, Krishna A, Harttgen K, Subramanian S. The association of parental education with childhood undernutrition in low-and middle-income countries: comparing the role of paternal and maternal education. International journal of epidemiology. 2017;46(1):312–323.

[38] Musa MK, Muhammad F, Lawal KM, Chowdhury A, Hossain A. Risk factors of severe acute malnutrition among under-five children: a hospital-based study in Bangladesh. J Med Sci Health. 2017;3:13–21.

[39] Naved RT. B angladesh, Families in. Encyclopedia of Family Studies. 2016;p. 1–5.

[40] Kim R, Mejia-Guevara I, Corsi DJ, Aguayo VM, Subramanian S. Relative importance of 13 correlates of child stunting in South Asia: Insights from nationally representative data from Afghanistan, Bangladesh, India, Nepal, and Pakistan. Social Science & Medicine. 2017;187:144–154.

[41] Pramod Singh G, Nair M, Grubesic RB, Connell FA. Factors associated with underweight and stunting among children in rural Terai of eastern Nepal. Asia Pacific Journal of Public Health. 2009;21(2):144–152.

[42] Rahman A, Chowdhury S. Determinants of chronic malnutrition among preschool children in Bangladesh. Journal of biosocial science. 2007;39(2):161–173.

[43] Rahman M. Association between order of birth and chronic malnutrition of children: a study of nationally representative Bangladeshi sample. Cadernos de saude publica. 2016;32:e00011215.

[44] Tödtling F, Trippl M. One size fits all?: Towards a differentiated regional innovation policy approach. Research policy. 2005;34(8):1203–1219.

[45] Ryan P, Lauver DR. The efficacy of tailored interventions. Journal of Nursing Scholarship. 2002;34(4):331–337.

[46] Schmidt EM. The effect of women's intrahousehold bargaining power on child health outcomes in Bangladesh. Undergraduate Economic Review. 2012;9(1):4.

Figure

Click here to access/download;Figure;Figure 1 - Classification Tree.eps

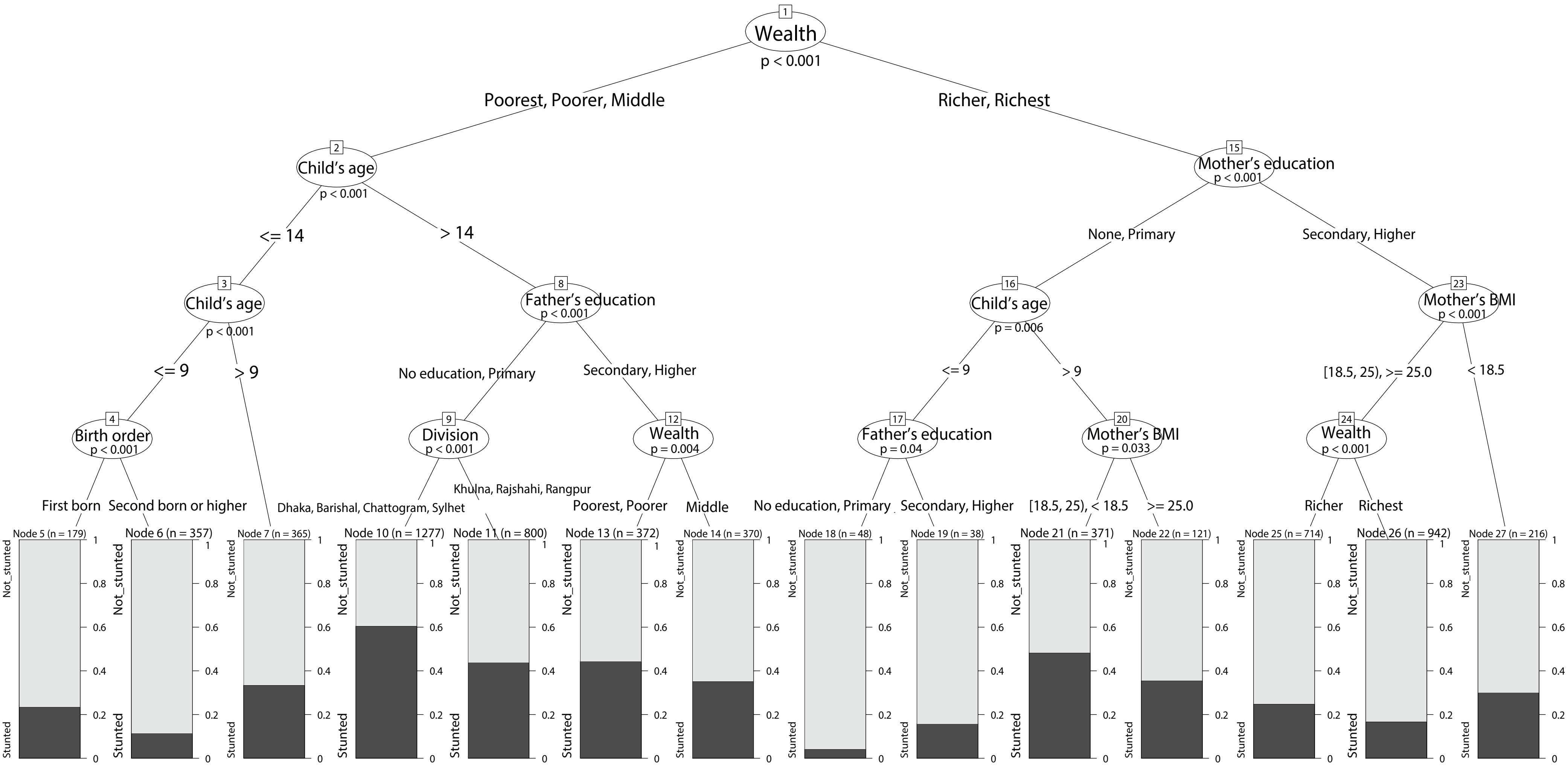

Supplement: Attachment — Submitted filename: PONE-D-20-31337_reviewed 9 Dec.pdf [file pone.0256729.s002.pdf]
