# Supplementary material for: Sociodemographic risk factors of under-five stunting in Bangladesh: Assessing the role of interactions using a machine learning method
Source: PLoS One. 2021 Aug 31;16(8):e0256729. doi: 10.1371/journal.pone.0256729 (PMC8407547; doi:10.1371/journal.pone.0256729)
Supplement: Attachment — Submitted filename: PONE-D-20-31337_R1 (1) due 15 Aug.pdf [file pone.0256729.s006.pdf]

PLOS ONE

Sociodemographic Risk Factors of Under-Five Stunting in Bangladesh: Assessing the Role of Interactions Using a Machine Learning Method

--Manuscript Draft--

**Manuscript Number:**	PONE-D-20-31337R1
**Article Type:**	Research Article
**Full Title:**	Sociodemographic Risk Factors of Under-Five Stunting in Bangladesh: Assessing the Role of Interactions Using a Machine Learning Method
**Short Title:**	Sociodemographic Risk Factors of Under-Five Stunting in Bangladesh: Assessing the Role of Interactions
**Corresponding Author:**	Mohaimen Mansur, Ph.D<br>University of Dhaka: Dhaka University<br>Dhaka, BANGLADESH
**Keywords:**	child stunting; malnutrition; machine learning; Classification tree; Public health; intervention; policy
**Abstract:**	Childhood stunting has been a persistent problem in Bangladesh with decades of interventions failing to reach the intended targets. Existing empirical studies investigating the problem in a socioeconomic context largely ignore the complex interplay of potential determinants that may be crucial for an intricate understanding of child malnutrition. This paper aims to demonstrate the importance of studying interactions among various sociodemographic risk factors of stunting with the help of an interpretable machine learning method. Data used for the analyses are extracted from the Bangladesh Demographic and Health Survey (BDHS) 2014 and pertain to a sample of 6,170 under-5 children. A classification tree was first constructed to identify important interaction-based rules that characterize children with different profiles of risk for stunting. Then binary logistic regression models were fitted to measure the importance of these interactions along with the individual risk factors. Results revealed that, as individual factors, living in Sylhet division (OR: 1.57; CI: 1.26 - 1.96), being an urban resident (OR: 1.28; CI: 1.03 - 1.96) and having working mothers (OR: 1.21; CI: 1.02 - 1.44) were associated with higher likelihoods of childhood stunting, whereas belonging to the richest households (OR: 0.56; CI: 0.35 - 0.90), higher BMI of mothers (OR: 0.68 CI: 0.56 - 0.84) and mothers' involvement in decision making about children's healthcare with father (OR: 0.83, CI: 0.71 - 0.97) were linked to lower likelihoods of stunting. Importantly however, risk classifications defined by the interplay of multiple sociodemographic factors showed more extreme odds ratios (OR) of stunting than single factor ORs. For example, children aged 14 months or above who belong to poor wealth class, have lowly educated fathers and reside in either Dhaka, Barisal, Chittagong or Sylhet division are the most vulnerable to stunting (OR: 2.52, CI: 1.85 - 3.44). The findings endorse the need for tailored-intervention programs for children based on their distinct risk profiles and sociodemographic characteristics.
**Order of Authors:**	Mohaimen Mansur, Ph.D
	Awan Afiaz
	Md. Saddam Hossain
**Response to Reviewers:**	We would like to thank the reviewers for their valuable comments, which have led to an improved version of the paper. The corrections that have been made in response to the comments are provided are as below. All of the suggested corrections are marked in red, while notable deletions are marked in blue strike-through.<br><br>Reviewer #1:<br>“Sociodemographic Risk Factors of Under-Five Stunting in Bangladesh: Assessing the Role of Interactions Using a Machine Learning Method” by Mansur M et al. is a well written manuscript with thorough analytical presentation on a very important aspect of childhood malnutrition, under 5-stunting. Childhood stunting is an important indicator of malnutrition which is prevalent in most developing countries, including Bangladesh. In spite of remarkable successes in several health indices, the rate of reduction of

childhood stunting in Bangladesh is much behind expectation. The authors have tried to find out the potential driving factors of childhood stunting in Bangladesh by studying interactions among various sociodemographic risk factors with the help of an interpretable machine learning method using data from a nationally representative survey¸ Bangladesh Demographic and Health Survey 2014. Their anticipation is that by tailored-intervention programs for children based on their distinct risk profiles and sociodemographic characteristics will help to achieve the national Health Nutrition and Population Sector Program objective of limiting the prevalence of stunting in children within 25% by the year 2022 and Goal 2.2 of the SDGs to end all forms of malnutrition including stunting in children under 5 years of age by the year 2030.
The manuscript is well written, yet I have few observations as mentioned below:

The 'Introduction' looks unnecessarily lengthy. The first three paragraphs (Lines 2-47) are fine as the introduction for the article. In the last 4 paragraphs (Lines 49-88) the authors have explained how they identified the important interactions capable of apprehending the complex interplay between the common sociodemographic variables by utilizing the classification tree to identify groups of children with various risk profiles for stunting and then further verified it through the use of widely accepted statistical models. They have also explained its superiority over the predominant use of regression models, either in the form of linear or logistic, used in previous literatures. The methods, authors used for the analyses, are elaborately described in 'Methodology' (Lines 146-206), So, such details can easily be avoided in 'Introduction'. It would be better if the authors briefly describe their methods here before the concluding sentence of previous paragraph (Lines 45-47) and comparison of their method with the methods used in previous studies briefly in 'Discussion'.
Authors' response:
We thank the reviewer for this detailed observation and apt suggestion. We have made the required changes in the 'Introduction' accordingly. Comparisons of results derived from the machine learning method in this study and those in literature are duly moved in the 'Discussion' section in the revised manuscript. All changes are marked in red.
The prediction in lines 15-18, "Furthermore, the chronological drop in the prevalence of stunting from 51% in 2004 to 31% in 2017 translates to an average annual rate of reduction (AARR) of 3.4% which falls short of the global AARR of 3.9% required to attain World Health Organization's target to reduce by 40% the global prevalence of stunting by 2025", might not be that straightforward. The reduction, like improvements in other health indices, probably was much faster in recent years.
Authors' response:
We thank the reviewer for this prudent observation. Indeed, while the overall AARR since 2004 is lower than required, the rate of reduction was indeed much faster in recent years. In order to clarify the problem better, we have now revised the quoted statement, and in the following statement posed the problem as 'demand for continued rapid reduction' rather than previously stated 'slow rate of reduction' [lines 23-26 in the revised manuscript].
It would be better to delete the sentence "Some of the recent studies are [9, 10, 11, 12, 13, 14, 15, 16, 17, 18, 19, 20, 21]" in Lines 33-34 and give the citations "9-21" after the previous sentence.
Authors' response:
Thank you. The required changes have been made following the reviewer's suggestion.

Childhood stunting (height-for-age) is an important, but not the only predictor of malnutrition; many stunting cases are either due to familial or constitutional. The anthropometric methods for measuring the nutritional status includes three widely used indicators to assess the growth of children: height-for-age, weight-for-height, and weight-for-age. Adding other parameters could strengthen the understanding of the objective of the study. It is obvious that in a single study it might not be possible to include all aspects, but at least can be mentioned as a limitation.
Authors' response:
We certainly agree with the reviewer's comment. We have now included this point as a limitation of the study in the 'Discussion' section. In particular, we have added the following sentence [lines 518-523 in the revised manuscript]:
"Firstly, this study investigated only height-for-age (stunting) in children and ignored weight-for-age (overweight) and weight-for-height (wasting) - the other two widely recognized anthropometric indicators of childhood malnutrition. While a complete

understanding of the drivers of childhood undernutrition will require involving all three aspects, the choice of looking at one is driven by the limited space for a single study."

I am not sure why the authors have not used data from Bangladesh Demographic and Health Survey 2017.
We started this study almost a year ago with an aim to apply our methods to the most recent data. Unfortunately, Bangladesh Demographic and Survey (BDHS) 2017 data were not available then and were released only on December 23, 2020 – almost three months after we have submitted our manuscript.
https://datacatalog.worldbank.org/dataset/bangladesh-demographic-and-health-survey-2017-2018
Furthermore, we would like to stress that the main objective of our study is to show the usefulness of the proposed machine learning assisted logistic regression method in capturing interactions among socio-demographic risk factors of childhood stunting, rather than provide a thorough critical analysis of current stunting situation in Bangladesh. We believe that the results of our study using BDHS 2014 data will not change much qualitatively if the recently released 2017 data were used. Moreover, the plethora of extant literature based on the 2014 BDHS dataset also plays to the advantage in the sense that the present analyses discussed in this paper is supported by sufficient literature and thereby indirectly validates the premise of the results obtained by our new methodological approach. However, we plan to investigate the possible temporal changes in a future study.

Reviewer #2:
Overall the manuscript is fine. The literature review has successfully addressed the objective of the research. The manuscript presented a nice application of machine learning method in public health research (data analysis) comparing with typical logistical model which is innovative idea. However, for a reader without having any prior knowledge of machine learning could be difficult to recognize the method.
No major issues for revision, but some minor issues to be addressed are as follows:

a) Line 225-226: "Working mothers' had a higher percentage of stunted children (39.9%)"-Are these working mothers from lower class? Have no education? Are they working in their own area and staying with their children or they migrate to other cities leaving children at home. Need a bit explanation otherwise it looks more generalized data.
Authors' response:
We thank the reviewer for the comment and enquiry. We added the following information about those working mothers following the quoted statement [lines 275-281].
"A deeper look into the data showed that the working-mother sample has almost 9 percentage point higher proportion of poor (poorest or poorer) individuals and 5 percentage point higher proportion of individuals with no formal education when compared to the non-working group. Furthermore, while the proportion of mothers with higher than secondary level education is only 1-percentage point higher in the working group, proportion of secondary-completed education is about 7-percentage point lower."
The detailed cross-table results are avoided in the revised article but provided below for the reviewer's consideration. However, we would like to clarify that other information on mothers' workplace such as possible migration status or area of work were not available in the dataset and thus not discussed in the paper.

Working StatusWealth IndexRow Total
PoorestPoorerMiddleRicherRichest
Not working9578068689609864577
(Row %), (Col %)20.9,   70.417.6,   69.519.0,   72.221.0,   76.421.5,   82.674.2
Working4023533342962081593
(Row %), (Col %)25.2,   29.622.2,   30.521.0,   27.818.6,   23.613.1,   17.425.8
P-value<0.001

Working StatusMother's EducationRow Total
No educationPrimarySecondaryHigher
Not working672126721834554577

Powered by Editorial Manager® and ProduXion Manager® from Aries Systems Corporation

(Row %), (Col %)14.7, 68.527.7, 73.447.7,
77.09.9, 72.674.2
Working3094606521721593
(Row %), (Col %)19.4, 31.528.9, 26.640.9,
23.010.8, 27.425.8
P-value<0.001

b) Line 229: The paper mentioned two specific areas (Sylhet and Khulna) of Bangladesh where the prevalence of stunning children were highest and lowest. Would you please explain which indicators were specifically worked for this variation between two areas?
Authors' response:
While we believe that it is difficult to identify the true indicators working behind the glaring variation between stunting percentages in the two divisions (Sylhet and Khulna) without a systematic investigation involving many potential risk factors, a quick look from our data showed that the Khulna division had the lowest percentage of illiterate (not formally educated) mothers (8.3%) and one of the lowest proportions of poorest mothers (18.9%). In contrast, the proportions of uneducated and poorest mothers are among the highest (27.9% and 30.3%, respectively) in Sylhet. These facts may explain part of the observed variation.

We added the detailed information in the text following the sentence [lines 286-291] and also provide the result below (not added in the manuscript) for the consideration of reviewer.

DivisionMother's EducationRow Total
No educationPrimarySecondaryHigher
Dhaka1833134541311081
(Row %), (Col %)16.9, 18.729.0, 18.142.0, 16.012.1, 20.917.5
Barisal7424432087725
(Row %), (Col %)10.2, 7.533.7, 14.144.1, 11.312.0, 13.911.8
Chattogram161284618931156
(Row %), (Col %)13.9, 16.424.6, 16.453.5, 21.88.0, 14.818.7
Khulna5716138188687
(Row %), (Col %)8.3, 5.823.4, 9.355.5, 13.412.8, 14.011.1
Rajshahi9919038285756
(Row %), (Col %)13.1, 10.125.1, 11.050.5, 13.511.2, 13.612.3
Rangpur12920034992770
(Row %), (Col %)16.8, 13.126.0, 11.645.3, 12.311.9, 14.712.5
Sylhet27833533151995
(Row %), (Col %)27.9, 28.333.7, 19.433.3, 11.75.1, 8.116.1
P-value<0.001

DivisionWealth IndexRow Total
PoorestPoorerMiddleRicherRichest
Dhaka1611531482613581081
(Row %), (Col %)14.9, 11.814.2. 13.213.7, 12.324.1, 20.833.1, 30.017.5
Barisal17020615711577725
(Row %), (Col %)23.4, 12.528.4, 17.821.7, 13.115.9, 9.210.6, 6.411.8
Chattogram1711722542922671156
(Row %), (Col %)14.8, 12.614.9, 14.822.0, 21.125.3, 23.223.1, 22.418.7
Khulna130120157155125687
(Row %), (Col %)18.9, 9.617.5, 10.422.9, 13.122.6, 12.318.2, 10.511.1
Rajshahi177151171146111756
(Row %), (Col %)23.4, 13.020.0, 13.022.6, 14.219.3, 11.614.7, 9.312.3
Rangpur24918214211879770
(Row %), (Col %)32.3, 18.323.6, 15.718.4, 11.815.3, 9.410.3, 6.612.5
Sylhet301175173169177995
(Row %), (Col %)30.3, 22.117.6, 15.117.4, 14.417.0, 13.517.4, 14.816.1
P-value<0.001

c) Line 235: Figure 1, there is no figure attached.

Authors' response:
Following PLOS ONE's submission guidelines the figure has been attached as a separate file which should be accessible to the reviewers.

d) In table 3, "Rules extracted from the classification tree" is a bit confusing. Is it a part logistical regression output?? If the objective of this table is to compare or find similarities then it can be placed in separate table.
Authors' response:
We apologize for the confusion that arose from the reported "Rules extracted from the classification tree" in table 3. To clarify, these are binary variables representing interactions detected in the classification tree and are part of the second logistic regression model. They did not enter the first 'No interaction' model, but entered the second logistic regression model as additional explanatory variables. We chose to present them in the same table in order to make a clear comparison between the two (with and without the rules/interactions) logistic regression models which we believe would be easier and beneficial to the readers.

e) Line 277: It would be better to understand when comparing two models, i.e. machine learning and logistic model in separate section
Authors' response:
We want to clarify that the model mentioned in line 277 is actually a logistic regression model which incorporated interactions from the machine learning method (classification tree) as additional explanatory variables. This interaction-augmented logistic regression model was then compared with a plain logistic regression model without interactions.

In this study the machine learning method is not presented as a contrasting method for logistic regression, but as a tool for effectively extracting complex interactions from data and then as a complementary method to develop more sophisticated interaction-induced logistic regression models which we posit would be an important addition to such analyses.
Other comments: Last but not the least, a simple definition with reference of Machine Learning method will be helpful for readers.
Authors' response:
This is an important suggestion and we have now included a simple definition of Machine Learning in the 'Methodology' section with reference [lines 185-188].

"Machine learning is largely viewed as data-driven methods that can automatically detect patterns from data and use those uncovered patterns to predict future or unseen data or to make decisions under uncertainty [26]. An important member of this group, a classification tree is a popular machine learning technique. It divides the sample data into small subgroups based on simple rules involving the predictors."
Reviewer #3:
Introduction:
Line :22: Height for age?
Authors' response:
We apologize for the unintentional typographical mistake which has now been corrected.

Discussion:
Please set table 4 probably mistakenly set in discussion section to the result segment
Authors' response:
We have now moved Table 4 to the Results section following the reviewer's advice.

Conclusion:
Please make it brief addressing objective
Line 419: Can be deleted as it is not a part of objective
Authors' response:
We thank the reviewer for this advice. We have now made the conclusion more concise to address specifically the objectives and deleted less relevant texts. All changes are marked in red in the revised manuscript.

Reviewer #4:
The article titled 'Sociodemographic Risk Factors of Under-Five Stunting in

Powered by Editorial Manager® and ProduXion Manager® from Aries Systems Corporation

Bangladesh: Assessing the Role of Interactions Using a Machine Learning Method' highlighted an important public health issues in Bangladesh. I have following review comments for the authors:
a. Share few key findings in Abstract, may few numbers for the readers who does not want to read the whole article, currently it is missing.
Authors' response:
We thank the reviewer for this valuable advice and updated the abstract by adding key findings. The relevant changes are marked in red in the revised manuscript with track changes as follows:

"Results revealed that, as individual factors, living in Sylhet division (OR: 1.57; CI: 1.26 - 1.96), being an urban resident (OR: 1.28; CI: 1.03 - 1.96) and having working mothers (OR: 1.21; CI: 1.02 - 1.44) were associated with higher likelihoods of childhood stunting, whereas belonging to the richest households (OR: 0.56; CI: 0.35 - 0.90), higher BMI of mothers (OR: 0.68 CI: 0.56 - 0.84) and mothers' involvement in decision making about children's healthcare with father (OR: 0.83, CI: 0.71 - 0.97) were linked to lower likelihoods of stunting. Importantly however, risk classifications defined by the interplay of multiple sociodemographic factors showed more extreme odds ratios (OR) of stunting than single factor ORs. For example, children aged 14 months or above who belong to poor wealth class, have lowly educated fathers and reside in either Dhaka, Barisal, Chittagong or Sylhet division are the most vulnerable to stunting (OR: 2.52, CI: 1.85 - 3.44)."

b. 2nd sentence in Introduction, please rephrase, it sounds like stunting remained as the only public health problem in Bangladesh is high childhood malnutrition
Authors' response:
We apologize for the unintentional misleading sentence and replaced it with the following:

"However, high childhood malnutrition has remained as one of the lasting national health problems." [lines 4-5]
The change is marked in red in the revised manuscript (with track changes).

c. Line 33-34 in Introduction section… Some of the recent studies are..it looks like an incomplete sentence, consider including these references in previous sentence.
Authors' response:
We thank the reviewer for pointing out this mistake which we have duly corrected in the revised version of the manuscript [lines 36-38].

d. 2.2 Response variable, sound little new. Is there any reason not to use Outcome variable following the norm?
Authors' response:
We have now changed the term 'Response variable' to commonly used 'Outcome variable'. The two terms are sometimes used interchangeably in statistics which is the source of the confusion.

e. Conclusion in discussion section: whole conclusion section needed to be modified, currently, it sounds like an abstract of an article, sentence starting from background, methods, justification of classification tree, strengths etc..…. I would add key findings and then continue with the what is there in later part of the second paragraph.
Authors' response:
We thank the reviewer for this valuable advice. The conclusion is now modified accordingly in the revised manuscript and have been marked in the red in the track changes version.

The nodes shown in Figure 1: Although apparent criticism of the standard classification tree related to biased selection of variables supposed to be overcome by conditional inference tree framework as mentioned in the methodology section; the presented may have some inherent limitation and pose internal validity of the method: for example, mother belonged to richer and richest arm, both mother and father had no education, but their children had lowest stunting!! Stringer justification needed.
Authors' response:
We appreciate the reviewer's observation in pointing out of the inherent limitation of classification tree. While we clearly stated in 'Classification tree' subsection of the

‘Results’ section that we discarded two tree nodes (the above-mentioned rule is one of them) on account of having low sample sizes (less than 50), we admit that we did not clarify the justification for these exclusions which we have included now. The main argument is that nodes with lower sample sizes (resulting from overgrown trees) often overfit the data under that node and are not generalizable to relevant population. This is indeed a common criticism of trees and we now also add this limitation in the methodology section. In particular we wrote [lines 207-210]:

“An inherent limitation of a classification tree in general, however, is that when it is grown indefinitely, node sample sizes become smaller and the tree overfits data meaning that it fits the noise more than the signal in the data [27]”.

Thus, while children coming from richer and richest household and having lowly educated parents may have the lowest stunting, there are only 48 such children in the sample of over 6000 children. It is not utterly surprising to think that the risk from low education of both parents overwhelmingly outweighed the possible gain from higher wealth for this very few children, but we should not consider this as generalizable to population of such children due to small sample size. All the sample sizes for the nodes we selected for regression are over 300.

Reviewer #5:
The manuscript titled “Sociodemographic Risk Factors of Under-Five Stunting in Bangladesh: Assessing the Role of Interactions Using a Machine Learning Method” is an excellent work at the current situation of Bangladesh. This analysis of Bangladesh Demographic and Health Survey (DHS) data describes a number of different factors associated with stunting using exception method of identification. However, there are some observations from my side stated below;
1. Abstract: It is surprising that none of the factors associated with stunting that were identified in the regression analysis and classification tree are mentioned in the abstract. It would be helpful to mention some of the most common risk factors in the abstract.
Authors’ response:
We thank the reviewer for bringing this important observation to our attention and we apologize for missing the key predictors of stunting in the abstract. We have now included them with associated odds ratios from the logistic regression analysis. The changes are marked in red in the revised manuscript.

2. Page 3, line 4: Sentence not clear, Is there any evidence (prevalence over time) that support the statement?
Authors’ response:
We have now added a reference as support for the mentioned statement in the revised manuscript [line 4].

3. Page 3 line 5: Stunting might be the “outcome” of nutrition deficiency rather than the “measure”.
Authors’ response:
We thank the reviewer for pointing out this mistake. We have now made the correction accordingly in the revised manuscript.

4. Line 93: Is there any specific reason behind using the 2014 DHS dataset? Since 2014 situation has been changed as GoB and NGOs took much strategies for child and maternal nutrition improvement targeting SDG goals.
Authors’ response:
We started this study almost a year ago with an aim to apply our methods to the most recent data. Unfortunately, Bangladesh Demographic and Survey (BDHS) 2017 data were not available then and were released only on December 23, 2020 – almost three months after we have submitted our manuscript.
https://datacatalog.worldbank.org/dataset/bangladesh-demographic-and-health-survey-2017-2018
While prevalence of under-five stunting reduced from 36% in 2014 to 31% in 2017 (possible following drives from GoB and NGOs as mentioned by the reviewer) according the latest BDHS report, as long as socio-demographic drivers of childhood stunting are concerned, we do not expect our results/finding to change qualitatively if the newly released data were used. The identified risk factors were historically

associated with under-five stunting in Bangladesh. However, we keep investigating any temporal changes between the last two survey periods as potential future research of interest.

Also, we would like to stress that the main objective of our study is to show the usefulness of the proposed machine learning method in capturing interactions among socio-demographic risk factors of childhood stunting, rather than to provide a thorough critical analysis of the current stunting situation in Bangladesh. Moreover, the plethora of extant literature based on the 2014 BDHS dataset also plays to the advantage in the sense that the present analyses discussed in this paper is supported by sufficient literature and thereby indirectly validates the premise of the results obtained by our new methodological approach.

5. Line 128: Initial and exclusive breast feeding, mode of delivery, child vaccination status, complementary feeding etc. are recognized as the indicators of children's growth in many studies. Can this be included as the independent variable upon data?
Authors' response:
We understand the reviewer's rightful concern for not including the above-mentioned nutrition-related variables. Our justifications for not considering the factors are as follows:

First, we found selecting the possible set of explanatory variables for childhood stunting in our study particularly challenging. Existing studies did not help much in guiding to a common or universally accepted set of indicators, and vary substantially in terms of the number and types of variables that range from demographic and socio-economic factors to spatial and health-nutrition related factors. The main aim of our paper is to better understand the role of socio-demographic risk factors of childhood stunting through their interactions identified through a machine learning method as opposed to identify and establish a comprehensive set of best predictors of stunting. Therefore, rather than considering all possible risk factors of childhood stunting (which are numerous), in this study we focused mainly on socio-demographic determinants of under-5 stunting and other control variables which commonly appeared significantly important in many relevant empirical studies, particularly those related to Bangladesh. For this reason, we were selective and did not consider the mentioned indicators, as well as many other likely determinants of childhood stunting such as number of visits by antenatal care provider and their qualifications, hygiene practices etc. We have listed this confined (as opposed to exhaustive) selection of explanatory variables as a primary limitation of our study in the 'Discussion' section.

Second, while reviewing the literature we found that many of our considered socio-demographic factors are closely related to the mentioned health and nutrition related indicators. For example, household wealth and/or mother's education has been consistently found to be associated with complementary feeding (e.g., Dhami et al., 2019, Nkoka et al., 2018), breastfeeding practices (e.g., Rahman et al., 2020), child vaccination (e.g., Sarker et al, 2019) and choice of mode of delivery (e.g., Yaya et al., 2019) and possibly capture some of their association with stunting indirectly.

Third, since vaccination, breastfeeding and complementary feeding requirements depend on age of children, investigating impact of these factors require age-stratified analysis which is beyond the scope of this study mainly looking at interactions of common socio-demographic factors on under-5 children as a whole.

Lastly, the interactions that would be considered in a joint sociodemographic-nutritional variables setting would be too large and require a more detailed approach. We believe such a study would require more in-depth data and analyses which would be out of the scope of this study. As mentioned previously, the main idea behind the study is to introduce a methodological contribution to the extant literature with the help of a machine learning method that is easily interpretable and allows the possibility to consider complex interactions between the variables largely ignored in typical public health studies.

We, however, understand the importance of looking at nutritional factors and plan to investigate the role of these factors and their interactions in a similar setting in a future study. We hope the reviewer would agree with our line of argument presented above.

Powered by Editorial Manager® and ProduXion Manager® from Aries Systems Corporation

6. Line 221: By categorizing children age in to 4 to 5 groups with around 12 months interval may provide insights to observe the stunting situation among children of different age groups in regression analysis. In many studies from the similar strata found that higher prevalence of stunting prevailed among children aged 36 to 47 months (Akram R, Mostafa Kamal SM, Gaire S, Darteh EKM)
Authors' response:
We thank the reviewer for this comment. We ran the regression (model with no-interaction) with children's age as a suggested categorical variable (please see the results below) which verify the reviewer's point: children aged 35-47 months are at the highest risk of stunting. While this is an interesting result showing possible non-linearity in age-stunting relationship we defend our treatment of age as continuous variables.

First, the classification tree works by optimally choosing at each step the most important variable and a binary cut-off point for that variable which maximizes discrimination between the binary response values. It is also capable of capturing non-linear association between the explanatory variable and the outcome by splitting on the same variable multiple times in subsequent steps. The main reason why we did not categorize children's age and treated it as a continuous variable in our analysis is that we wanted to allow the classification tree to automatically choose the optimal cut-off ages which will maximize discrimination between stunted and non-stunted children. Categorizing age into predefined groups would have hindered this flexibility. If there were any significantly important non-linearity in age-stunting relationship we expected it to appear in our tree, which it unfortunately did not happen. And, to keep the entire analysis consistent we treated age also as a continuous variable in the logistic regression models. Nonetheless, we understand that such subtle non-linearity may be missed if the size of the tree is kept relatively small.

Second, the age-categorized regression did not really alter our results. The coefficients (apart from age) of the explanatory variables in the two regressions (age: categorical vs continuous) change only slightly. But importantly, the broad finding remains the same. Children of higher ages are more or less at higher risks of stunting when compared to infants (please see the classification tree in Figure 1). While the age-specific variation in stunting prevalence is interesting, we believe that it will not prove to be of high significance in addressing the aim of the paper which is exploring interaction among explanatory variables.

Last, the use age as a continuous variable in months rather than a categorical variable with 12-month categories actually allows a more parsimonious model to be had with fewer number of parameters estimated and also permits the interpolation of results within the range of 0-59 months.

VariablesOdds Ratio95% CIp-value
Age of child (categorized in years)
[0,11]1.00
(11,23]3.362.50, 4.51<0.001
(23,35]3.872.81, 5.32<0.001
(35,47]4.673.59, 6.08<0.001
(47,59]3.222.36, 4.40<0.001
Sex of child
Male1.00
Female0.960.84, 1.110.614
Child's birth order
11.00
21.000.74, 1.360.981
More than 20.950.60, 1.510.831
Decision maker regarding child's healthcare
Husband or Else1.00
Mother0.810.65, 0.990.045
Parents0.830.71, 0.970.018
Age of mother at first birth (in years)0.980.95, 1.010.282
No of children alive to mother
11.00
21.270.94, 1.720.125

Powered by Editorial Manager® and ProduXion Manager® from Aries Systems Corporation

3 or more1.170.74, 1.860.506
Mother's BMI
[18.5, 25)1.00
< 18.51.130.89, 1.440.325
>= 25.00.610.50, 0.75<0.001
Mother's education level
None1.00
Primary1.050.78, 1.420.738
Secondary0.850.63, 1.140.276
Higher0.950.61, 1.480.821
Father's education level
No education1.00
Primary0.890.75, 1.050.168
Secondary0.710.56, 0.910.006
Higher0.530.37, 0.76<0.001
Mother's employment status
Not-working1.00
Currently working1.211.02, 1.440.032
Mother's age difference to the father0.990.98, 1.000.129
Wealth index
Poorest1.00
Poorer0.890.73, 1.070.214
Middle0.730.59, 0.900.004
Richer0.580.45, 0.75<0.001
Richest0.350.25, 0.49<0.001
Area of Residence
Rural1.00
Urban1.331.06, 1.650.012
Administrative division
Dhaka1.00
Barishal1.120.86, 1.450.414
Chattogram1.190.96, 1.480.109
Khulna0.670.52, 0.860.002
Rajshahi0.660.52, 0.82<0.001
Rangpur0.880.67, 1.160.365
Sylhet1.571.27, 1.94<0.001

7. Table 1: Mothers education level: Does it mean "No formal education"?
Authors’ response:
For mother’s education in Table 1 the category “No education” indeed meant “No formal education” and has been used as the reference category in the logistic regression. We have added the following line of clarification in the ‘Independent variables’ subsection of the ‘Materials and Methods’ section where mother’s education was introduced [line 164-165].

“It is important to mention that the 'no education' category for both parents' education implies 'no formal education.”

8. Discussion: Can this be explained that how maternal BMI and birth order and current work status of mothers are linked to childhood stunting?
Authors’ response:
We have now added possible explanations for the association found between childhood stunting and the above-mentioned factors with references in the ‘Discussion’ section.
The main arguments are as follows:
1) A mother’s BMI is representative of her own physical attributes, stature and nourishment. It is, therefore, not surprising that we found children having low-BMI mothers to be at higher risk of stunting.
2) Research found that higher order children are often subject to shorter duration of breastfeeding, lower prenatal and postnatal investments and lower care-time from parents. These probably explain the association between stunting and age and birth order - a result we found and also confirmed by existing research.
3) It may seem unusual that children of working mothers appeared to be more likely to

Powered by Editorial Manager® and ProduXion Manager® from Aries Systems Corporation

be stunted. However, as reported in the 'Results' section, higher percentages of both poor and illiterate individuals in the working-mother sample may partly explain this association.
However, the cross-sectional nature of the data do not allow us to venture further into the probable causes. The relevant references are not listed here, but are provided in the revised manuscript.

Reviewer #6:
Review report of the manuscript no. PONE-D-20-31337

Abstract section, the result is not well explained because it was not fulfill to the objectives. I think it was much differ to objectives and results.
Authors' response:
We thank the reviewer for this comment. We have now included the key findings in the abstract and excluded some policy suggestions in order to make it more informative and reflective of the objectives. The main changes that are marked in red in the revised manuscript are as follows:
"Results revealed that, as individual factors, living in Sylhet division (OR: 1.57; CI: 1.26 - 1.96), being an urban resident (OR: 1.28; CI: 1.03 - 1.96) and having working mothers (OR: 1.21; CI: 1.02 - 1.44) were associated with higher likelihoods of childhood stunting, whereas belonging to the richest households (OR: 0.56; CI: 0.35 - 0.90), higher BMI of mothers (OR: 0.68 CI: 0.56 - 0.84) and mothers' involvement in decision making about children's healthcare with father (OR: 0.83, CI: 0.71 - 0.97) were linked to lower likelihoods of stunting. Importantly however, risk classifications defined by the interplay of multiple sociodemographic factors showed more extreme odds ratios (OR) of stunting than single factor ORs. For example, children aged 14 months or above who belong to poor wealth class, have lowly educated fathers and reside in either Dhaka, Barisal, Chittagong or Sylhet division are the most vulnerable to stunting (OR: 2.52, CI: 1.85 - 3.44)."

Authors could not make clear in a good way what authors were said to understand the line 4-8
Authors' response:
We have now modified some sentences and added reference to improve the clarity of the points we made in the mentioned lines. Changes are marked in red in the revised manuscript.

28-31 lines were not obviously comprehend.
Authors' response:
We are sorry for any unclarity from our part. In the mentioned lines we tried to explain that since childhood stunting had been a prevalent health problem in Bangladesh it attracted much academic research. Major area of investigation of these studies were identification of socio-demographic risk factors of childhood stunting.

42-46 lines not satisfied for me and eventually objectives were not associated with the title.
Authors' response:
We have added the following subsequent texts to the mentioned lines in order to clarify our objectives in a better way.

"A major contribution of the paper is methodological which involves utilizing a classification tree, a method borrowed from the machine learning arena, to automatically detect the complex interactions among the common risk factors of childhood stunting. Importance of these interactions are then evaluated through the use of classical logistic regression models. Interplay of multiple factors show higher association with stunting and provide deeper insight to the persistent public health problem of the country."

Please also note that we have substantially curtailed the 'Introduction' by moving parts of it to the 'Discussion section following further suggestions. We hope it now communicates our objectives in a better and precise way.

49-55 lines explain more but it was not fruitful of the title. Even if the authors were not

Powered by Editorial Manager® and ProduXion Manager® from Aries Systems Corporation

able to present it properly.
Authors’ response:
We thank the reviewer for this comment and apologize for the lack of clarity from our part. Please note that the entire paragraph has now been removed from the ‘Introduction’ to make it more concise. Some of the arguments in the paragraph, particularly those about relative advantages of classification tree over classical regression models, are moved to the ‘Methodology’ section. The additions are marked in red in the revised manuscript.

66-77 lines authors wrote extend but it was repeated. Over all the introduction thrives could not well describe and inadequate.
Authors’ response:
We understand the reviewer’s concern. We want to iterate that following further suggestions for improving the lack of clarity of the ‘Introduction’ and making it concise, we have modified the section substantially. Thus, the mentioned texts are removed from the ‘Introduction’ and moved to the ‘Discussion’ section of the revised manuscript.

106-107 lines what were authors said not clear for me.
Authors’ response:
We thank teh reviewer for this comment. We have modified the sentence as follows to improve its clarity:

“In addition to a number of health and nutrition relation information, the BDHS survey} also collected measurements on height and weight from eligible women of reproductive ages and children under the age of 5 years.”

Responsive variable portion wrote too much and I thought easily and curtly discuss. Same case as independent variables elucidate too much and unnecessary.
Authors’ response:
We understand the reviewer’s observation. We have now deleted the entire first paragraph of the ‘Response variable’ (now changed to ‘Outcome variable’ in the revised manuscript) to make the subsection more concise. However, we believe some small details were necessary to clearly introduce the nature and measurement of the various explanatory variables with their different categories/values and therefore, we could not curtail the ‘Independent variable’ section. We hope the reviewer will understand our point.

147-206 methodology section wrote enlarge and few were repeated one or more times. Which had no resemblance to this manuscript?
Authors’ response:
While we understand the reviewer’s concern, we would like to emphasize that the methodology we used in this paper, particularly the machine learning method and its application in conjunction with classical statistical models, is a relatively new concept in public health research and we wanted to make sure that it is described with adequate clarity. We, therefore, felt that the classification tree with its relative advantages and disadvantages, generation of binary rules from its interaction results, model selection criteria including cross-validation, adjustment of results for survey weights, statistical packages used etc. be touched upon and introduced in proper sequence. We hope the reviewer will understand our inability to shorten the ‘Methodology’ section despite our best efforts due to the trade-off between length and clarity. We also want to point out that we avoided description of unnecessary details of familiar methods such as the logistic regression.

What were the eligibility criteria? On the whole data sources and materials portion is pitiable presentation.
Authors’ response:
We have reported the eligibility criteria in the ‘Data Overview’ subsection. Precisely, children under 5-years of age whose anthropometric measures of stunting (height-for-age) were available were considered for the study, but de facto residents (temporary residents), twin births and cases with missing observations on other information were excluded.
235-239 lines I didn't understand what were authors want to recognize.
Authors’ response:

Powered by Editorial Manager® and ProduXion Manager® from Aries Systems Corporation

In these lines we were describing left part of the classification tree originated from the root. The subsequent appearance of the variables from the root indicated the order of importance. Thus, wealth is the most important predictor of stunting as it is placed at the top. Age is the next significant predictor with children aged below 14 months have lower prevalence of stunting. In order to relate the texts to the classification tree (Figure 1) better, we now included the prevalence of stunted children (60%) for the node explained.
241-250 lines were not clearly arrangement.

Authors' response:
We have made the following modifications in the referred texts in order to improve clarity.

"The right-hand branch originated from the root of the classification tree include the wealthier households. On this side, mother's education, and then their body mass index (BMI) as well as the age of a child were notable predictors of stunting. Here, children who have mothers with no/low formal education, who are older than 9 months and have relatively lower BMI (node 21) demonstrate high risk of child stunting (around 48%)."[lines 303-307].

Changes are marked in red.

Lines 380 to 400. I didn't think these findings are relevant. The really relevant results are that the only educational level that seems to be protective is the highest level of education, as well as the only socioeconomic level is the richest.
Authors' response:
We would like to point out that the referred texts at lines 380-392 of the original manuscript are merely policy implications of our findings rather than findings themselves, and apologize for any lack of clarity on this matter from our part. Please note that we have now modified the possible policy values in an attempt to make it more relevant to our results following suggestions from other reviewers [lines 487-502]..

We, however, agree with the reviewer that the emphasis we gave to the established association between mother's decision making on child's healthcare and childhood stunting in lines 394-405 of the original manuscript may not need separate attention as such as it can be treated as a secondary result not particularly relevant to the interactions we highlighted. We have now curtailed the details of this finding and moved it to the part of the 'Discussion' section where individually significant variables were discussed [lines 449-454].

Discussion portion could not present appropriately. In many cases authors could enlighten haphazardly. Authors could have accessible it in a simpler way. Authors end up writing it shortly.
Authors' response:
Please note that we have now modified the 'Discussion' section by including parts from the introduction and adding clarifying explanations in order to make it as coherent and relevant as possible. All the changes are marked in red in the revised manuscript.

All in all, authors could not nearby it well. But their title of the manuscript is very good and the methods that authors like are definitely appropriate for this time. But overall presentation is not appropriate of the manuscript.
Authors' response:
We appreciate the reviewer's encouraging words. We have now put substantial efforts to make the presentations of the paper as coherent and as organized as possible.

Reviewer #7:
It is good to see that the researchers considered a number of independent variables at children's individual, parental and household level. These factors are useful for understanding the childhood stunting from socioeconomic point of views. However, there are some important issues such as low birth weight (LBW), infant and young child feeding (early initiation of breastfeeding, exclusive breastfeeding, breastfeeding continuation, and complementary feeding) which the nutrition professionals and policy makers are highly concerned about. Could the authors please consider including these

Powered by Editorial Manager® and ProduXion Manager® from Aries Systems Corporation

factors into analysis or provide justification why these factors were not considered?
Authors' response:
We understand the reviewer's rightful concern for not including the above-mentioned nutrition-related variables. Our justifications for not considering the factors are as follows:

First, we found selecting the possible set of explanatory variables for childhood stunting in our study particularly challenging. Existing studies did not help much in guiding to a common or universally accepted set of indicators, and vary substantially in terms of the number and types of variables that range from demographic and socio-economic factors to spatial and health-nutrition related factors. The main aim of our paper is to better understand the role of socio-demographic risk factors of childhood stunting through their interactions identified through a machine learning method as opposed to identify and establish a comprehensive set of best predictors of stunting. Therefore, rather than considering all possible risk factors of childhood stunting (which are numerous), in this study we focused mainly on socio-demographic determinants of under-5 stunting and other control variables which commonly appeared significantly important in many relevant empirical studies, particularly those related to Bangladesh. For this reason, we were selective and did not consider the mentioned indicators, as well as many other likely determinants of childhood stunting such as number of visits by antenatal care provider and their qualifications, hygiene practices etc. We have listed this confined (as opposed to exhaustive) selection of explanatory variables as a primary limitation of our study in the 'Discussion' section.

Second, while reviewing the literature we found that many of our considered socio-demographic factors are closely related to the mentioned health and nutrition related indicators. For example, household wealth and/or mother's education has been consistently found to be associated with birthweight (e.g., Khan et al., 2018, Manyeh et al., 2016), complementary feeding (e.g., Dhami et al., 2019, Nkoka et al., 2018) and breastfeeding practices (e.g., Rahman et al., 2020), and possibly capture some of their association with stunting indirectly.

Third, since breastfeeding and complementary feeding requirements depend on age of children, investigating impact of these factors require age-stratified analysis which is beyond the scope of this study mainly looking at interactions of common socio-demographic factors on under-5 children as a whole.

Lastly, the interactions that would be considered in a joint sociodemographic-nutritional variable setting would be too large and require a more detailed approach. We believe such a study would require more in-depth data and analyses which would be out of the scope of this study. As mentioned previously, the main idea behind the study is to introduce a methodological contribution to the extant literature with the help of a machine learning method that is easily interpretable and allows the possibility to consider complex interactions between the variables largely ignored in typical public health studies.

We, however, understand the importance of looking at nutritional factors and plan to investigate the role of these factors and their interactions in a similar setting in a future study. We hope the reviewer would agree with our line of argument presented above.

Abstract: Kindly mention what type of variables were considered in the statistical model. Health Nutrition and Population Sector Program (HNPSP) mainly focuses on health and nutrition related indicators rather than socio-demographic aspects. Kindly re-phrase how the findings could be useful in national planning and policy making to reach the SDG target.
Authors' response:
We understand the reviewer's point and admit that it is naïve to link the findings of the paper directly to meeting of HNPSP and SDG targets. We have now excluded this sentence from the abstract. Rather than making direct policy suggestions we emphasize targeting vulnerable children identified through distinct risk profiles and interactions of socio-demographic characteristics, learning more about their nutritional and other requirements and accordingly address multiple risk factors in designing policies to mitigate their stunting problems.

Powered by Editorial Manager® and ProduXion Manager® from Aries Systems Corporation

Discussion: “The complex interplay of multiple factors usually go unnoticed”—the interaction is well perceived by the nutrition experts, but the evidences are undocumented.
Authors’ response:
Here, we wanted to emphasize that the complex interplay of multiple factors is not directly observable when investigating pertinent factors individually. These interactions are not of course empirically well-investigated. However, but we apologize for the lack of clarity of the statement. We have now modified the sentence as following, appreciating acknowledgment of interactions by nutrition experts and researchers.

“These insights stemming from the complex interplay of multiple factors is not directly observable when looking at the pertinent factors individually. While the importance of these interactions is well perceived by the nutrition experts, supporting empirical evidences are largely undocumented.”

Conclusion: It seems the summary of every section. Kindly emphasize what the findings interpret which is well understood and sensitize relevant professionals from field level to policy level.
Authors’ response:
We thank the reviewer for the suggestion. We have now curtailed and modified the conclusion to highlight the results and their policy values. All changes are highlighted in red in the revised manuscript.

Reviewer #8:
Response- Title needs to be enreach and shorter, type of study needs to be mentioned in the title.
Authors’ response:
We thank the reviewer for the suggestion. We, however, find it difficult to keep a balance between keeping the title short and adding more information to it, e.g., the type of study. Since the major contribution of the paper is methodological, we wanted to highlight the use of machine learning method in the title. We hope the reviewer will understand our point.

The findings is not elaborately mentioned in the abstract, result of OR need to be more specifically mentioned in abstract, The statistical analysis is not enough in the abstract.
Authors’ response:
We fully agree with the reviewer and apologize for under-reporting key results in the abstract. We have now included more detailed findings with ORs as advised which are as follows:

“Results revealed that, as individual factors, living in Sylhet division (OR: 1.57; CI: 1.26 - 1.96), being an urban resident (OR: 1.28; CI: 1.03 - 1.96) and having working mothers (OR: 1.21; CI: 1.02 - 1.44) were associated with higher likelihoods of childhood stunting, whereas belonging to the richest households (OR: 0.56; CI: 0.35 - 0.90), higher BMI of mothers (OR: 0.68 CI: 0.56 - 0.84) and mothers' involvement in decision making about children's healthcare with father (OR: 0.83, CI: 0.71 - 0.97) were linked to lower likelihoods of stunting. Importantly however, risk classifications defined by the interplay of multiple sociodemographic factors showed more extreme odds ratios (OR) of stunting than single factor ORs. For example, children aged 14 months or above who belong to poor wealth class, have lowly educated fathers and reside in either Dhaka, Barisal, Chittagong or Sylhet division are the most vulnerable to stunting (OR: 2.52, CI: 1.85 - 3.44).”
”
References

Dhami, M.V., Ogbo, F.A., Osuagwu, U.L. and Agho, K.E., 2019. Prevalence and factors associated with complementary feeding practices among children aged 6–23 months in India: a regional analysis. BMC public health, 19(1), pp.1-16.

Khan, J.R., Islam, M.M., Awan, N. and Muurlink, O., 2018. Analysis of low birth weight and its co-variants in Bangladesh based on a sub-sample from nationally representative survey. BMC pediatrics, 18(1), pp.1-9.

Manyeh, A.K., Kukula, V., Odonkor, G., Ekey, R.A., Adjei, A., Narh-Bana, S., Akpakli,

	D.E. and Gyapong, M., 2016. Socioeconomic and demographic determinants of birth weight in southern rural Ghana: evidence from Dodowa Health and Demographic Surveillance System. BMC pregnancy and childbirth, 16(1), pp.1-9.<br><br>Nkoka, O., Mhone, T.G. and Ntenda, P.A., 2018. Factors associated with complementary feeding practices among children aged 6–23 mo in Malawi: an analysis of the Demographic and Health Survey 2015–2016. International health, 10(6), pp.466-479.<br><br>Rahman, M.A., Khan, M.N., Akter, S., Rahman, A., Alam, M.M., Khan, M.A. and Rahman, M.M., 2020. Determinants of exclusive breastfeeding practice in Bangladesh: Evidence from nationally representative survey data. Plos one, 15(7), p.e0236080.<br><br>Sarker, A.R., Akram, R., Ali, N. and Sultana, M., 2019. Coverage and factors associated with full immunisation among children aged 12–59 months in Bangladesh: insights from the nationwide cross-sectional demographic and health survey. BMJ open, 9(7), p.e028020.<br><br>Yaya, S., Bishwajit, G. and Gunawardena, N., 2019. Socioeconomic factors associated with choice of delivery place among mothers: a population-based cross-sectional study in Guinea-Bissau. BMJ global health, 4(2), p.e001341.
**Additional Information:**	
**Question**	**Response**
**Financial Disclosure**<br><br>Enter a financial disclosure statement that describes the sources of funding for the work included in this submission. Review the submission guidelines for detailed requirements. View published research articles from *PLOS ONE* for specific examples.<br><br>This statement is required for submission and **will appear in the published article** if the submission is accepted. Please make sure it is accurate.	The author(s) received no specific funding for this work.

<table>
<tr><td>Unfunded studies
Enter: The author(s) received no specific funding for this work.

Funded studies
Enter a statement with the following details:
• Initials of the authors who received each award
• Grant numbers awarded to each author
• The full name of each funder
• URL of each funder website
• Did the sponsors or funders play any role in the study design, data collection and analysis, decision to publish, or preparation of the manuscript?
• NO - Include this sentence at the end of your statement: The funders had no role in study design, data collection and analysis, decision to publish, or preparation of the manuscript.
• YES - Specify the role(s) played.

* typeset</td><td></td></tr>
<tr><td>Competing Interests

Use the instructions below to enter a competing interest statement for this submission. On behalf of all authors, disclose any competing interests that could be perceived to bias this work—acknowledging all financial support and any other relevant financial or non-financial competing interests.

This statement will appear in the published article if the submission is accepted. Please make sure it is accurate. View published research articles from PLOS ONE for specific examples.</td><td>The authors have declared that no competing interests exist.</td></tr>
</table>

Powered by Editorial Manager® and ProduXion Manager® from Aries Systems Corporation

**NO authors have competing interests**<br><br>Enter: *The authors have declared that no competing interests exist.*<br><br>**Authors with competing interests**<br><br>Enter competing interest details beginning with this statement:<br><br>*I have read the journal's policy and the authors of this manuscript have the following competing interests: [insert competing interests here]*<br><br>* typeset	
**Ethics Statement**<br><br>Enter an ethics statement for this submission. This statement is required if the study involved:<br><br>• Human participants<br>• Human specimens or tissue<br>• Vertebrate animals or cephalopods<br>• Vertebrate embryos or tissues<br>• Field research<br><br>Write "N/A" if the submission does not require an ethics statement.<br><br>General guidance is provided below. Consult the submission guidelines for detailed instructions. **Make sure that all information entered here is included in the Methods section of the manuscript.**	This article does not contain any studies with human participants performed by any of the authors. The Bangladesh demographic and health Surveys were approved by ICF Macro Institutional Review Board and the National Research Ethics Committee of the Bangladesh Medical Research Council. A written consent about the survey was given by participants before the interview. All identification of the respondents was dis-identified before publishing data. The secondary data sets analysed during the current study are freely available upon request from the DHS website at https://dhsprogram.com/data/available-datasets.cfm. Searching 'Bangladesh DHS, 2014' in the DHS website will provide the survey data set.

**Format for specific study types**<br><br>**Human Subject Research (involving human participants and/or tissue)**<br>• Give the name of the institutional review board or ethics committee that approved the study<br>• Include the approval number and/or a statement indicating approval of this research<br>• Indicate the form of consent obtained (written/oral) or the reason that consent was not obtained (e.g. the data were analyzed anonymously)<br><br>**Animal Research (involving vertebrate animals, embryos or tissues)**<br>• Provide the name of the Institutional Animal Care and Use Committee (IACUC) or other relevant ethics board that reviewed the study protocol, and indicate whether they approved this research or granted a formal waiver of ethical approval<br>• Include an approval number if one was obtained<br>• If the study involved *non-human primates*, add *additional details* about animal welfare and steps taken to ameliorate suffering<br>• If anesthesia, euthanasia, or any kind of animal sacrifice is part of the study, include briefly which substances and/or methods were applied<br><br>**Field Research**<br><br>Include the following details if this study involves the collection of plant, animal, or other materials from a natural setting:<br>• Field permit number<br>• Name of the institution or relevant body that granted permission	
**Data Availability**<br><br>Authors are required to make all data underlying the findings described fully available, without restriction, and from the time of publication. PLOS allows rare exceptions to address legal and ethical concerns. See the PLOS Data Policy and FAQ for detailed information.	No - some restrictions will apply

A Data Availability Statement describing where the data can be found is required at submission. Your answers to this question constitute the Data Availability Statement and **will be published in the article**, if accepted.<br><br>**Important:** Stating 'data available on request from the author' is not sufficient. If your data are only available upon request, select 'No' for the first question and explain your exceptional situation in the text box.<br><br>Do the authors confirm that all data underlying the findings described in their manuscript are fully available without restriction?	
**Describe where the data may be found in full sentences. If you are copying our sample text, replace any instances of XXX with the appropriate details.**<br><br>• If the data are **held or will be held in a public repository**, include URLs, accession numbers or DOIs. If this information will only be available after acceptance, indicate this by ticking the box below. For example: *All XXX files are available from the XXX database (accession number(s) XXX, XXX.*).<br>• If the data are all contained **within the manuscript and/or Supporting Information files**, enter the following: *All relevant data are within the manuscript and its Supporting Information files.*<br>• If neither of these applies but you are able to provide **details of access elsewhere**, with or without limitations, please do so. For example:<br><br>*Data cannot be shared publicly because of [XXX]. Data are available from the XXX Institutional Data Access / Ethics Committee (contact via XXX) for researchers who meet the criteria for access to confidential data.*<br><br>*The data underlying the results presented in the study are available from (include the name of the third party*	The secondary data sets analysed during the current study are freely available upon request from the DHS website at https://dhsprogram.com/data/available-datasets.cfm. Searching 'Bangladesh DHS, 2014' in the DHS website will provide the survey data set.

Powered by Editorial Manager® and ProduXion Manager® from Aries Systems Corporation

and contact information or URL).<br>• This text is appropriate if the data are owned by a third party and authors do not have permission to share the data.<br><br>* typeset	
Additional data availability information:	

Sociodemographic Risk Factors of Under-Five Stunting in Bangladesh: Assessing the Role of Interactions Using a Machine Learning Method

Mohaimen Mansur[1*], Awan Afiaz[1], Md. Saddam Hossain[2]

1 Institute of Statistical Research and Training, University of Dhaka, Dhaka, Bangladesh

2 Population Council, Dhaka, Bangladesh

* Corresponding author

E-mail: mmansur@isrt.ac.bd

Abstract

Childhood stunting has been a persistent problem in Bangladesh with decades of interventions failing to reach the intended targets. Existing empirical studies investigating the problem in a socioeconomic context largely ignore the complex interplay of potential determinants that may be crucial for an intricate understanding of child malnutrition. This paper aims to demonstrate the importance of studying interactions among various sociodemographic risk factors of stunting with the help of an interpretable machine learning method. Data used for the analyses are extracted from the Bangladesh Demographic and Health Survey (BDHS) 2014 and pertain to a sample of 6,170 under-5 children. A classification tree was first constructed to identify important interaction-based rules that characterize children with different profiles of risk for stunting. Then binary logistic regression models were fitted to measure the importance of these interactions along with the individual risk factors. Results revealed that risk classifications defined by the interplay of multiple sociodemographic factors showed more extreme odds ratios (OR) of stunting than single factor ORs. The findings endorse the need for tailored-intervention programs for children based on their distinct risk profiles and sociodemographic characteristics. Such targeted efforts could prove to be cost-effective and more efficient in achieving the Health Nutrition and Population Sector Program (HNPSP) objective of limiting the prevalence of stunting in children within 25% by the year 2022 and aid in attaining the Sustainable Development Goal 2.2 of ending all forms of child malnutrition in Bangladesh.

Keywords: child stunting, malnutrition, machine learning, classification tree, public health, intervention, policy.

1 Introduction

Despite many socioeconomic challenges, Bangladesh achieved remarkable successes in many areas of public health during the last two decades which include a significant reduction in child and maternal mortality [1]. What remained as a lasting health problem for the country, however, is high childhood malnutrition. A key measure of this nutrition deficiency is low height-for-age, commonly known as stunting, which reflects the aggregate repercussions of undernutrition in children since birth or even prenatal stages, indicating long-term restriction on a child's growth potential [2]. The current situation of childhood stunting in Bangladesh is alarming. According to the latest round of Bangladesh Demographic and Health Survey conducted in 2017, almost one-third of the under-five children fail to reach a standard height for their ages [3]. This figure is one of the highest in the world outside Sub-Saharan Africa, and largely overshadows the 12 percentage-point decline in the prevalence of stunting that has been achieved by Bangladesh over the last decade [3].

Furthermore, the chronological drop in the prevalence of stunting from 51% in 2004 to 31% in 2017 translates to an average annual rate of reduction (AARR) of 3.4% which falls short of the global AARR of 3.9% required to attain World Health Organization's target to reduce by 40% the global prevalence of stunting by 2025 [4]. At the current reduction rate, Bangladesh is also unlikely to achieve its government's 4th Health Nutrition and Population Sector Program (HNPSP) objective of limiting the prevalence of stunting in children within 25% by the year 2022 [3]. This steady but slow rate of reduction in childhood stunting, coupled with a high prevalence, presents a persistent threat to the national health status of children in Bangladesh. It is now well-known that in addition to fatal consequences like child mortality low age-for-height also has long-term health effects including chronic illness, disabilities, and cognitive impairment [5, 6]. Moreover, stunted children are likely to be physically, intellectually, and economically less productive in adulthood than well-nourished children [7, 8].

The long-standing problem of childhood stunting in Bangladesh has duly attracted substantial attention in academic research. A major interest of this vast literature lies in identifying the sociodemographic determinants of stunting, a key step for designing effective socioeconomic policy interventions targeted to reduce this public health problem. Risk factors that commonly

appeared in many empirical studies include children's age, gender, birth order, underweight mothers, low levels of parent's education, and poor socioeconomic status, among others. Some of the recent studies are [9, 10, 11, 12, 13, 14, 15, 16, 17, 18, 19, 20, 21]. Rarely addressed in these existing papers, however, is the possible complex interaction among different sociodemographic features of children. It is often the intricate interplay of multiple risk factors rather than individual factors that characterize potential risks of young-age undernourishment. For example, while nourishment is generally low in children whose families are poor or children whose mothers are not educated, it is probably even lower in children who are exposed to both these risk factors. From a policy perspective, this would mean that improving both education and economic conditions of the most vulnerable group may yield a faster and more efficient reduction in stunting. The main argument of this paper stems from the fact that studies that ignore these possible interactions are likely to provide an inadequate understanding of the sociodemographic disparities driving childhood stunting and therefore, are likely to offer limited policy values. This study, therefore, aims to justify the merits of incorporating such interactions among potential driving factors of childhood stunting in Bangladesh by using data from a nationally representative survey.

One of the possible reasons why interactions between variables did not feature much in the empirical public health research including nutrition studies is their predominant use of regression models, either in the form of linear or logistic. These statistical models are not best suited to capture and analyze interactions, particularly when the important ones are not known in advance. Typically, the number of possible interactions increases drastically with the number of variables incorporated in a regression model, leading to the curse of dimensionality [22]. This eventually results in the over-parametrization of models and subsequent difficulties in estimation.

In this paper, we go against the convention and exploit the classification tree, a method borrowed from the machine learning arena, to identify important interactions capable of apprehending the complex interplay between the sociodemographic variables discussed in the literature. Classification trees scale up well to large datasets and use combinations of explanatory variables to stratify data into various risk groups. Unlike parametric regression models, this nonparametric method offers a more flexible data-driven approach to fit complex data and also

offers an easy and intuitive interpretation of results [23].

While classification trees are being increasingly used in many areas of public health research, its applications in the field of childhood nutrition are still few [24, 25, 26]. To our knowledge, only one paper [27] used classfication trees to discover risk factors of childhood stunting in the context of Bangladesh. While the paper uses a rich set of biological, socioeconomic, and environmental information on children under two years of age, the sample data is limited only to low-income participants from a small district (smaller regions under divisions) of rural Bangladesh. Consequently, findings from the paper are not directly generalizable to the relevant child population in Bangladesh which very likely exhibits wider sociodemographic and spatial variations. In contrast, our study utilizes data from a population-based nationally-representative survey. A fundamental difference of this paper with these existing studies is that in addition to identifying interactions among sociodemographic determinants of stunting it further measures the importance and statistical significance of these interactions through applications of classical regression models.

The current analyses aim to make important methodological contributions by utilizing the classification tree to identify groups of children with various risk profiles for stunting. The importance of such risk stratification is further verified through the use of widely accepted statistical models. Importantly, this risk profiling is characterized by the interaction of multiple sociodemographic features of children allowing the possibility for targeted interventions based on their respective vulnerabilities. Considering the fact that Bangladesh is observing an undesirably slow reduction in childhood stunting, such need-specific tailored policies can speed up the progress towards achieving the Goal 2.2 of the Sustainable Development Goals (SDGs) that aims to end all forms of malnutrition including stunting in children under 5 years of age by the year 2030 [28].

2 Materials and Methods

2.1 Data overview

This study used data from the Bangladesh Demographic and Health Survey (BDHS) 2014. This is a nationally representative cross-sectional survey conducted in Bangladesh in collaboration with the Demographic and Health Survey (DHS) and is operated by Measure DHS+ under the authority of the National Institute of Population Research and Training (NIPORT). The survey sampling frame was based on the 2011 Bangladesh population census, and a two-stage stratified cluster sampling technique was employed in the survey. In the first stage, 600 enumeration areas (EAs), a geographic area consisting of 113 households on average, were independently selected with probability proportional size (PPS) method from all 20 sampling strata. Then using an equal probability systematic selection in the second stage, 30 households were selected from each cluster (EA). The survey resulted in 17,886 completed interviews with ever-married women between the ages of 15 to 49. Further details of the sampling procedure can be found in [29].

The survey also collected measurements on height and weight from eligible women of reproductive ages and children under the age of 5 years. Complete and credible anthropometric and age data on 7,318 children were collected in the process [29]. After excluding the temporary (de facto) residents, children of twin births and any cases with missing values in the variables, the final sample size for this study was 6,170.

2.2 Response variable

Anthropometric measurements of height and weight were used to assess the nutrition status of Bangladeshi children under 5 years of age. The BDHS 2014 data constructed several indices from these measurements according to the WHO Child Growth Standards, such as stunting, wasting, and underweight, that takes into account the variations in ethnicity, social and economic influences, and feeding practices worldwide [30]. These indices provide important information about a child's growth and body composition and act as key indicators in order to assess their nutritional status.

This study concerns only the height-for-age or stunting index which reflects the cumulative

effect of constant malnourishment as well as recurrent and chronic illnesses resulting in lack of growth among children compared to their respective ages. Two standard deviations below the median (-2 SD) of the WHO reference population in terms of height-for-age indicates that the child is short for his or her age, i.e. stunted [30]. This variable was pre-calculated according to the definition provided by WHO in the BDHS 2014 child dataset.

2.3 Independent variables

Based on the literature review and pre-analysis, the current study explored the variables that commonly appeared in most nutrition related studies on Bangladesh. At the child level, these included the age of children in months (0-59 months), sex (male, female), as well as the birth order (first born, second born, and third born or above) of the child and the decision-maker regarding child's healthcare needs (mother only, both parents, and husband and/or someone else). Independent variables related to the mother such as mother's age at the birth of her first child (in years), number of living children (one, two, and three or more children), body mass index (BMI) level (less than 18.5, between 18.5 and 25, and greater than 25), education level (no education, primary, secondary, higher secondary or above), current employment status (currently working, not working) and mother's age difference to the father (in years) along with father's education level (no education, primary, secondary, higher secondary or above) were analyzed.

The sociodemographic variables, related to the child's household characteristics, were also explored for association with stunting in children under 5 years of age, which included household's wealth index (poorest, poorer, middle, richer, richest), area of residence (urban, rural) and administrative division (Dhaka, Barishal, Chattogram, Khulna, Rajshahi, Rangpur, Sylhet). The wealth index quintiles were pre-calculated using principal component analysis on household assets [31] and were readily available in the dataset.

2.4 Methodology

The analyses of the paper were carried out in two steps using a machine learning approach and a classical regression model in succession. In the first step of the analysis, a classification tree was fit to the data to identify possible interactions among predictors of childhood stunting. In the second step, the interactions identified by the trees are incorporated in a logistic

regression framework in addition to individual explanatory variables, and their impact on the likelihood of stunting measured. This two-step method can be thought to be a simpler version of the *Rulefit* approach proposed in [32] where an ensemble of trees rather than a single tree is used to identify important interaction rules which are then included in a penalized regression model.

A classification tree divides the sample data into small subgroups based on simple rules involving the predictors. The rules originate from step-wise binary splits of the predictors where the most important predictor at each step (referred to as a node) is selected for splitting. The tree is produced with an aim to achieving minimum error in classification (e.g., whether a child is stunted or not) for subgroups produced at each node. A classification tree is nonparametric in nature and holds a number of advantages over parametric regression type models including minimal distributional assumptions and easier interpretation. The most important advantage, however, is the tree's inherent capability of capturing complex, such as non-linear, interactions of variables in high dimensional settings [23]. The standard classification tree techniques such as CART [33] are often criticized for biased selection of variables which have many possible splits and missing values. These problems have been rectified in a conditional inference tree framework proposed in [34] that has been adopted in the current analyses. The depth of the tree was fixed to four layers to keep a balance between capturing adequate complexity and avoiding too low (less than 50) sample size per node.

While trees offer flexible approaches to reveal interactions among explanatory variables from the sample data, it does not provide measurable sizes of effects of these interactions on the response (childhood stunting) or their statistical significance. To achieve these, interactions/rules were incorporated in a logistic regression setting as additional predictors. Rules yielding either a stunting prevalence of greater than 40% or less than 20% were considered suggestive of significantly high and low prevalence, respectively, and considered for entering into the regression model. These thresholds for rule selection were constructed based around the national prevalence of under-five stunting of 36% in Bangladesh [29].

Each selected rule is then converted to a binary variable taking a value of 1 when the defining criteria for that particular rule are met, and a value of 0, otherwise. Each of these binary

variables is, thus, defined by unique interactions of several sociodemographic features of children. Next, two logistic regression models were estimated and compared. In the first model, only the initially selected independent variables were fitted to the stunting outcome (stunted, not stunted) and no interaction rule was considered. But in the second model, binary variables from selected rules were included along with the individual variables. Survey weights are duly applied in both the classification and logistic regression for making the sample results representative of the population of interest. Findings from regression models are further adjusted for the complexity of the survey design to capture cluster- and strata-wise variations and appropriately compute the standard errors and p-values for regression estimates.

Finally, classification accuracies of the two logistic regression models are systematically evaluated for both in-sample goodness of fit and out-of-sample predictive performance. Comparisons are made by computing standard criteria such as accuracy, sensitivity, specificity and the area under the Receiver Operating Characteristic Curve (ROC). The first three criteria are calculated based on a probability threshold of 0.5. To obtain the area under the curve (AUC) for the out-of-sample ROCs, a 10-fold cross-validation approach was undertaken and the mean AUC values were reported in the findings.

To ensure the reproducibility in scientific research in cases of new discoveries, the present study followed the recommendation by [35] in identifying the significant interaction rules. That is, the proposed interaction terms will be considered significant if the corresponding p-values were less than 0.005 in the second model. All analyses were performed in R *(version 3.6.0)*. The binary logistic regression models were fitted using the *"survey"* package *(version 4.0)* and the classification tree was constructed using the *"partykit"* package *(version 1.2-7)*.

3 Results

3.1 Descriptive statistics

Table 1 presents the distribution of stunting in under-five children by their sociodemographic characteristics considered in the study. Although the age of the children was considered as a continuous numeric variable in later analysis, it was converted into a categorical variable for exploratory reasons in this table. The overall stunting prevalence was 36.5% in the study

data. The results reveal that children aged two years or older were more stunted (41.8%) than younger children (28.3%). Children of higher birth order were also stunted in higher proportions (42.7%) than the first or second-born children.

Table 1: Distribution of stunting by sociodemographic variables (categorical and continuous) among children aged 0-59 months

**Sociodemographic variables**	**Group**	**N (%) of study participants**	**N (%) of Stunted**
**Age of child (in months)**	0 – 23 months	2432 (39.4)	689 (28.3)
	24 – 59 months	3738 (60.6)	1563 (41.8)
**Sex of child**	Male	3167 (51.3)	1175 (37.1)
	Female	3003 (48.7)	1077 (35.9)
**Child's birth order**	First born	2260 (36.6)	730 (32.3)
	Second born	1885 (30.6)	657 (34.9)
	Third born or above	2025 (32.8)	865 (42.7)
**Decision maker regarding child's healthcare**	Mother only	948 (15.4)	319 (33.6)
	Both parents	3493 (56.6)	1252 (35.8)
	Husband or Somebody else	1729 (28.0)	681 (39.4)
**Age of mother at first birth (mean [SD])**		18.17 [3.23]	
**No of children alive to mother**	One child	2060 (33.4)	636 (30.9)
	Two children	2122 (34.4)	756 (35.6)
	Three or more	1988 (32.2)	860 (43.3)
**Mother's BMI**	Less than 18.5	1387 (22.5)	633 (45.6)
	18.5 – 25.0	3619 (58.7)	1330 (36.8)

Table 1 – *Continued*

**Sociodemographic variables**	**Group**	**N (%) of study participants**	**N (%) of Stunted**
	Greater than 25.0	1164 (18.9)	289 (24.8)
**Mother's education level**	No Education	981 (15.9)	491 (50.1)
	Primary	1727 (28.0)	770 (44.6)
	Secondary	2835 (45.9)	866 (30.5)
	Higher	627 (10.2)	125 (19.9)
**Father's education level**	No education	1564 (25.3)	773 (49.4)
	Primary	1881 (35.5)	767 (40.8)
	Secondary	1854 (30.0)	541 (29.2)
	Higher	871 (14.2)	171 (19.6)
**Mother's employment status**	Currently working	1593 (25.8)	635 (39.9)
	Not working	4577 (74.2)	1617 (35.3)
**Mother's age difference to father (median)**		8.0	
**Household wealth index**	Poorest	1359 (22.0)	697 (51.3)
	Poorer	1159 (18.8)	476 (41.1)
	Middle	1202 (19.5)	450 (37.4)
	Richer	1256 (20.4)	394 (31.4)
	Richest	1194 (19.3)	235 (19.7)
**Area of residence**	Rural	4229 (68.5)	1623 (38.4)
	Urban	1941 (31.5)	629 (32.4)
**Administrative division**	Dhaka	1081 (17.5)	366 (33.9)

Table 1 – *Continued*

**Sociodemographic variables**	**Group**	**N (%) of study participants**	**N (%) of Stunted**
	Barishal	725 (11.8)	278 (38.3)
	Chattogram	1156 (18.7)	431 (37.3)
	Khulna	687 (11.1)	190 (27.7)
	Rajshahi	756 (12.2)	227 (30.0)
	Rangpur	770 (12.5)	272 (35.3)
	Sylhet	995 (16.1)	488 (49.0)
**Total sample size**	N	6170	2252 (36.5)

The mean age of mothers at their first birth was 18.2 years with a standard deviation of 3.23 years. Fathers were mostly older than mothers with a median age difference of 8 years. Children belonging to mothers with three or more living children were more stunted (43.3%) than those households where they were the only child (30.9%) or had only one sibling (35.6%). The prevalence of stunting is also lower (33.6%) in cases where the mother was the sole decision-maker regarding the child's healthcare needs in comparison to cases where decisions are made by the husband or another member of the family (39.4%). The prevalence of stunting for children belonging to underweight mothers was more than 20% higher than that in children born to mothers who were overweight (24.8%). Reduced percentages of stunting were observed for higher levels of educational attainment of both mother and father. Working mothers had a higher percentage of stunted children (39.9%). The proportion of stunted children was also high at the lowest wealth quintile (51.3%) but decreased with higher levels of wealth. Percentage stunted in rural households (38.4%) was higher than the national average of 36% while that in urban households was below (32.4%). The prevalence of stunting was the highest in the Sylhet division (49%) and the lowest in the Khulna division (27.7%).

3.2 Classification tree

The classification tree in **Figure 1** shows the interplay of important sociodemographic factors behind stunting in children under five years of age. The wealth reveals itself to be the most important predictor being placed at the top of the tree. Children belonging to middle or

Figure 1: Classification tree for stunting in under-five children in Bangladesh

The value of n at each final node gives the node sample size and the black fill in the box below indicates proportion of stunted children under the node. The p-value at each node corresponds to a unified permutation test of hypothesis indicating independence between the response and predictors [34].

lower categories of wealth statuses are placed under the left branch. The age of a child is the next most important variable in this branch showing children younger than 14 months to be less vulnerable to stunting. Other noteworthy predictors on this side of the tree were father's education, administrative division, and the birth order of the child. In this case, children older than 14 months with lowly educated fathers living in Dhaka, Barishal, Chattogram, and Sylhet divisions (node 10) seemed to be at the highest risk of being stunted.

On the right-hand branch originated from the root, for wealthier households, mother's education, and then their body mass index (BMI) as well as the age of a child were notable predictors of stunting. Here, mothers having only lower levels of education with children older than 9 months and a lower BMI (node 21) demonstrate increased risk of child stunting. From the classification tree, we chose six interaction rules defined by six nodes (6, 10, 11, 13, 21, and 26, respectively) to be important for our analysis. The previously stated national prevalence of stunting of 36% has been utilized to determine the thresholds for these rule selections. A node was considered to be of significant importance if it showed the prevalence of stunting to be higher than 40% (high risk) or less than 20% (low risk). However, we excluded nodes 18 and 19 from further consideration on account of having considerably low sample sizes. Selected six nodes were then transformed into binary variables based on whether the observations satisfied all four classification criteria under each node, displayed in **Table 2**, and are named as "Rule-6, 10, 11, 13, 21, and 26" respectively.

3.3 Logistic regression models

Results from the logistic regressions are presented in **Table 3**. The first model which included only the sociodemographic variables in the study, exhibits that stunting was positively associated with the age of a child (OR 1.02 with 95% CI: 1.015, 1.024), corroborating the findings from the descriptive statistics. The likelihood of stunting reduced by nearly 20% when the mother was the sole decision-maker of the child's healthcare needs (OR 0.81 with 95% CI: 0.66, 0.99) or when both parents jointly made such decisions (OR 0.83 with 95% CI: 0.71,

Table 2: Interaction rules extracted from the Classification tree and associated stunting

Rule ID	Rule combinations	Node frequency	Stunting prevalence
**Rule 6**	Wealth index = Poorest, Poorer, Middle & Age of child <= 14 & Age of child <= 9 & Birth order = Second born, Third born or above	357	11.50%
**Rule 10**	Wealth index = Poorest, Poorer, Middle & Age of child > 14 & Father's education level = No education, Primary & Division= Dhaka, Barisal, Chittagong, Sylhet	1277	60.50%
**Rule 11**	Wealth index = Poorest, Poorer, Middle & Age of child > 14 & Father's education level = No education, Primary & Division= Khulna, Rajshahi, Rangpur	800	43.90%
**Rule 13**	Wealth index = Poorest, Poorer, Middle & Age of child > 14 & Father's education level = Secondary, Higher & Wealth index = Poorest, Poorer	372	44.40%
**Rule 21**	Wealth index = Richer, Richest & Mother's education level = No education, Primary & Age of child > 9 & Mother's BMI = Less than 18.5, Between 18.5 to 25	371	48.20%
**Rule 26**	Wealth index = Richer, Richest & Mother's education level = Secondary, Higher & Mother's BMI = Between 18.5 to 25 , Greater than 25 & Wealth index = Richest	942	16.70%

0.97) as opposed to the father or somebody else being in charge of such decision-making. The odds of stunting reduced by 39% (p-value < .001) for mother's with a BMI greater than 25, in comparison to mothers with a BMI score between 18.5 and 25. Father's education also played a part with both secondary (OR 0.73 with 95% CI: 0.58, 0.92) and higher (OR 0.54 with 95% CI: 0.39, 0.75) categories showing a significantly lower likelihood of stunting compared to fathers with no education.

Table 3: Odds ratios and associated inference from logistic regression models

Sociodemographic factors	Model 1: No interaction OR (95% CI)	p-value	Model 2: With interaction rules OR (95% CI)	p-value
**Age of child (in months)**	1.02 (1.015, 1.024)	<.001	1.00 (1.00, 1.01)	0.094
**Sex of child (ref: Male)**				
Female	0.97 (0.84, 1.12)	0.666	0.95 (0.83, 1.09)	0.489
**Child's birth order (ref: First born)**				
Second born	1.08 (0.81, 1.45)	0.597	1.06 (0.78, 1.44)	0.698
Third born or above	1.02 (0.64, 1.61)	0.941	1.11 (0.70, 1.75)	0.663
**Decision maker regarding child's healthcare (ref: Husband or somebody else)**				
Mother only	0.81 (0.66, 0.99)	0.04	0.82 (0.66, 1.01)	0.063
Both parents	0.83 (0.71, 0.97)	0.019	0.83 (0.71, 0.97)	0.021
**Age of mother at first birth**	0.98 (0.95, 1.01)	0.177	0.98 (0.95, 1.01)	0.236
**No of children alive to mother (ref: One child)**				
Two children	1.18 (0.87, 1.58)	0.288	1.34 (0.98, 1.82)	0.067
Three or more children	1.09 (0.68, 1.75)	0.722	1.14 (0.71, 1.81)	0.591
**Mother's BMI (ref: Between 18.5 to 25.0)**				
Less than 18.5	1.15 (0.91, 1.45)	0.237	1.11 (0.88, 1.44)	0.367
Greater than 25.0	0.61 (0.50, 0.75)	<.001	0.68 (0.56, 0.84)	<.001
**Mother's education level (ref: No education)**				
Primary	1.09 (0.81, 1.46)	0.565	1.05 (0.78, 1.41)	0.76
Secondary	0.89 (0.66, 1.19)	0.43	1.03 (0.77, 1.38)	0.847
Higher	0.99 (0.64. 1.54)	0.973	1.18 (0.77, 1.83)	0.449
**Father's education level (ref: No education)**				
Primary	0.88 (0.74, 1.04)	0.124	0.90 (0.75, 1.08)	0.256

Table 3 – *Continued*

Sociodemographic factors	Model 1: No interaction OR (95% CI)	p-value	Model 2: With interaction rules OR (95% CI)	p-value
Secondary	0.73 (0.58, 0.92)	0.007	0.92 (0.71, 1.20)	0.545
Higher	0.54 (0.39, 0.75)	<.001	0.72 (0.51, 1.03)	0.07
**Mother's employment status (ref: Not working)**				
Currently working	1.21 (1.01, 1.44)	0.034	1.21 (1.02, 1.44)	0.032
**Mother's age difference to father**	0.99 (0.98, 1.00)	0.084	0.99 (0.97, 1.00)	0.069
**Wealth Index (ref: Poorest)**				
Poorer	0.86 (0.71, 1.04)	0.117	0.83 (0.68, 1.01)	0.061
Middle	0.72 (0.59, 0.89)	0.002	0.77 (0.62, 0.95)	0.017
Richer	0.58 (0.45, 0.75)	<.001	0.65 (0.49, 0.87)	0.004
Richest	0.36 (0.26, 0.49)	<.001	0.56 (0.35, 0.90)	0.016
**Area of Residence (ref: Rural)**				
Urban	1.29 (1.04, 1.60)	0.02	1.28 (1.03, 1.59)	0.028
**Administrative division (ref: Dhaka)**				
Barishal	1.11 (0.85, 1.44)	0.438	1.11 (0.85, 1.46)	0.433
Chattogram	1.20 (0.97, 1.47)	0.092	1.20 (0.97, 1.48)	0.099
Khulna	0.67 (0.53, 0.86)	0.002	0.80 (0.61, 1.05)	0.114
Rajshahi	0.65 (0.52, 0.82)	<.001	0.82 (0.64, 1.05)	0.122
Rangpur	0.88 (0.68, 1.14)	0.338	1.07 (0.81, 1.42)	0.632
Sylhet	1.51 (1.23, 1.86)	<.001	1.57 (1.26, 1.96)	<.001
**Rules extracted from the classification tree**				
**Rule-6 (ref: No)**				
Yes			0.22 (0.15, 0.33)	<.001
**Rule-10 (ref: No)**				
Yes			2.52 (1.85, 3.44)	<.001
**Rule-11 (ref: No)**				
Yes			1.57 (1.09, 2.07)	0.001

Table 3 – *Continued*

**Sociodemographic factors**	**Model 1: No interaction**		**Model 2: With interaction rules**	
	**OR (95% CI)**	**p-value**	**OR (95% CI)**	**p-value**
**Rule-13 (ref: No)**				
Yes			1.64 (1.15, 2.33)	0.006
**Rule-21 (ref: No)**				
Yes			2.01 (1.41, 2.86)	<.001
**Rule-26 (ref: No)**				
Yes			0.65 (0.43, 0.99)	0.047

Interestingly, having a working mother increased the odds of stunting by 21% (p-value: 0.034) compared to mothers who were not employed at the time of the survey. Children from middle, rich and, the richest households were 28%, 42%, and 64% less at risk of stunting compared to children from poorest households (p-value <.005). Furthermore, children from urban areas were significantly more likely (OR 1.29 with 95% CI: 1.04, 1.60) to be stunted than children in rural areas. Children living in Sylhet division had 51% higher risk of being stunted (p-value <.001) compared to Dhaka, whereas the burden of risk was more than 30% lower for children living in Khulna (OR 0.67 with 95% CI: 0.53, 0.86) and Rajshahi (OR 0.65 with 95% CI: 0.52, 0.82).

The second model, where the interaction rules extracted from the classification tree were included along with the sociodemographic variables, showed somewhat different results. Age of children, the mother being the sole decision-maker of child's healthcare, father's education level and administrative divisions (barring Sylhet) were no longer associated with stunting individually at 5% level of significance. However, the rest of the significant associations remained as they were with minor changes in the odds of either increasing or decreasing the likelihood of stunting.

Following the suggestion by [35], the interaction rules 6, 10, 11, and 21 were significantly associated with stunting at 0.5% level of significance. The children corresponding to rule-6, indicating those who live in the poorest, poorer or middle-wealth households, who are younger

than 9 months and who rank second or higher in the birth order, were 78% less at risk of stunting than those who do not belong to this rule. According to rule-10, odds of stunting in children of lower wealth categories who are more than 14 months old, have primary or uneducated fathers and live in any one of Dhaka, Barisal, Chittagong, Sylhet divisions was 2.5 times the odds in the children who do not fall into these categories. In contrast, according to rule-11, children with same wealth, age and paternal education levels as in rule-10, exhibit an odds ratio of only 1.54 when they resided in any one of the other three divisions - Khulna, Rajshahi, or Rangpur. It is also worth noting that, children aged greater than 14 months who belonged to the poorest or poorer household but have secondary or higher educated fathers were also at 64% higher odds of stunting (rule-13 with p-value 0.006). Finally, based on rule-21, children aged greater than 9 months who belonged to the richer or richest households with mothers having little or no formal education and a BMI of 25 or less were twice as likely to be stunted as children who have different other combinations of risk factors.

3.4 Model validation

The predictive performances of both logistic regression models were evaluated to assess whether the addition of decision tree rules as interactions in the second model played any part in improving the results. For out of sample predictions a 10-fold cross-validation procedure was followed. **Table 4** shows that, when fitting in-sample data is concerned, including the decision tree rules in the model yielded a more than 18% increase in the sensitivity at the cost of a marginal drop in specificity (2.35%). Similarly, in the out-of-sample predictions, sensitivity improved by 22% with a slight drop in specificity. In both scenarios, larger areas under the ROC curve (AUC) further confirmed the benefit from adding the interaction-induced rules in the model.

4 Discussion

The present analysis assessed the role of interactions between important sociodemographic factors, commonly identified in the literature, in explaining childhood stunting. The findings from the two-step analysis provided several interesting perspectives. In the exploratory part of the analysis, the classification tree identified the specific groups of under-five children with various levels of risk for stunting. Essentially, each of these different risk profiles was charac-

Table 4: Comparison of model performance

Performance type	Sensitivity	Specificity	Accuracy	AUC
**In-sample (full sample fit)**				
Model 1: Without interaction	0.366	0.847	0.671	0.692
Model 2: With interaction	0.440	0.832	0.689	0.715
**Out-of-sample (10-fold cross validation)**				
Model 1: Without interaction	0.360	0.850	0.674	0.694
Model 2: With interaction	0.436	0.824	0.684	0.712

Sensitivity, specificity and accuracy are computed based on a probability threshold of 0.5. Results for out-of-sample performances are averages across 10-fold cross-validations. AUC is the area under the Receiver Operating Characteristic (ROC) curve.

terized by a unique combination of several predictively important sociodemographic features and provided noteworthy insights into the phenomenon of childhood stunting. These insights stemming from the complex interplay of multiple factors usually go unnoticed when looking at the pertinent factors individually.

For example, the distribution of stunted children by household wealth quintile reported in **Table 1** shows that the percentage of stunted children in households belonging to the richer and richest wealth statuses were 31.4% and 19.7%, respectively. However, node 21 of the classification tree in **Figure 1** (Rule 21 in Table 2) revealed that a sub-group of children from these wealth classes who were above 9 months of age and born to illiterate or lowly educated (primary education) mothers having low to moderate BMI (BMI < 25), have a much higher (48%) prevalence of stunting. Note that, the use of an interpretable machine learning method has divulged insights with more depth than what would normally be achieved.

Similarly, vulnerable groups with analogous risk profiles could be characterized by very different combinations of risk factors with the help of classification trees. Evidently, children under node 11 of the classification tree (Rule 11 in Table 2) had only 4% lower risk of stunting than those in the aforementioned node 21, but possess noticeably dissimilar features concerning household wealth and parental education.

Findings from the classification tree corroborate what we already know about childhood stunting in developing countries including Bangladesh while providing us with fresh perspec-

tives. Sociodemographic variables that appeared in the tree have been largely identified as significant predictors of child growth in the literature as well as in the factor-only logistic regression model of this study (Model 1 of Table 3). The prevalence of adverse conditions and food insecurity in underprivileged households is well-documented and so is the association between poverty and stunting [36, 20]. Mother's educational attainment is an important factor for a child's nourishment [12, 37] as this corresponds to women's knowledge regarding mothering a child and their subsequent care-taking. Paternal education has been also found to be relevant [38, 17] as it is linked to increased earning and health awareness, particularly in low- and middle-income households, and complements maternal education in many cases [39]. Furthermore, mother's own physical attributes and nourishment, indicated by mother's BMI, is a significant aspect affecting the child's health as well [40, 41]. Higher age and higher birth order are also frequently linked to childhood stunting [42, 43]. Individually considered, all of these are undoubtedly important factors. However, the interplay between these factors offered a deeper understanding of the sociodemographic effect on child malnutrition which is likely to be crucial in designing targeted intervention strategies.

In the confirmatory part of the analysis, selected groups of children with low and high risks of stunting, as identified by the tree, were incorporated into a logistic regression model (Model 2 in Table 3) together with the individual risk factors and were investigated for their possible associations with stunting. Here, all but one association were statistically significant at least at 1% level of significance. More importantly, the strength of associations of these interaction-induced risk groups with stunting, measured by odds ratio (OR), were predominantly higher (for positive associations) or lower (for negative associations) than those for individual risk factors.

As an example, the highest odds ratio for an individual variable was 1.57, observed for the Sylhet division, implying that living in this division increased the likelihood of stunting by 57% compared to the residents in the Dhaka division. In contrast, the odds ratios for positively associated rules are in the range of 1.57 to 2.52, the highest corresponding to rule 10 reported in Table 2 and implying that the odds of stunting for children living in certain divisions including Sylhet and possessing specific risk features was 2.5 times the odds for children having other characteristics. Similarly, among the individual predictors, children belonging to the richest

household wealth quintile were associated with the lowest odds ratio (OR) of 0.56, while the OR for a negatively associated rule was as low as 0.22 (rule 6 in Table 2).

The combination of results from the classification tree and the logistic regression may bear valuable implications on research and policies related to stunting in under-five children. Identification of vulnerable groups and specific interactions of multiple sociodemographic factors that define these groups can aid in expanding the understanding of different aspects of stunting. Policymakers working towards the reduction of stunting may utilize this intricate knowledge in designing risk-specific tailored interventions by targeting the characterizing features.

One policy recommendation based on the findings of this paper will pertain to targeting relatively poor and lowly educated families living in Sylhet, Chattogram, Barishal, or Dhaka divisions who have the highest risk of stunting and attempt to increase post-primary education and employment among them. These can be achieved by making higher education free or cheaper in these communities and by creating low-capital income-generating opportunities. The second set of intervention programs could be formulated by focusing on encouraging higher female education, with a particular emphasis on mother and child's health literacy, as well as ensuring proper nutrition among mothers in relatively richer households. Such small-scale targeted interventions can be quicker and more effective, but less expensive in achieving the desired reduction in stunting as opposed to a nation-wide implementation of a more general policy such as making higher female education free of cost in the entire country. Importance of such tailored interventions are emphasized in disciplines like innovation studies [44] and nursing care [45].

While the focus of the paper is the evaluation of interaction among sociodemographic characteristics of children as determinants of stunting, a by-product of the findings of the analysis is that children whose healthcare decisions are made either by the mother alone or jointly by both parents have significantly lower odds of stunting than children whose decisions are made solely by the father or someone else. This could also be a key learning in the understanding of the sociodemographic mechanism behind childhood stunting in Bangladesh and is in line with findings in the literature [46]. Intervention programs aimed at increasing the practice of granting mothers' autonomy in child's healthcare decision-making or encouraging mothers' in-

clusion in such decisions could go a long way in achieving the goal of alleviating this problem. Furthermore, interventions programs that already include educating mothers regarding child's health and nourishment could also be positively impacted by the inclusion of mothers in such decision-making practices.

The present study has a few limitations. Firstly, the secondary data was obtained from a cross-sectional survey as opposed to a longitudinal study and so no causal relationship between the factors and the outcome could be established. Secondly, the selection of explanatory variables is limited to those that generally appeared significant in major nutritional studies rather than all possible inclusions from an exhaustive search of the literature. Thirdly, the tree depth was limited to four layers for avoiding too low sample size per node and two nodes were excluded from analysis as a result even after exceeding the pre-specified thresholds.

5 Conclusion

High prevalence of childhood stunting and low rate of reduction per year imply that Bangladesh is falling behind in meeting national and international targets for reducing early-age undernutrition. This paper emphasizes the importance of studying the possible interplay of different sociodemographic determinants of child stunting, largely missing from existing literature, for a deeper insight into the problem. With that aim, analyses adopted in the paper exploit a classification tree to uncover complex interactions from data and incorporate them in a logistic regression model. Significantly high and low magnitudes of odds ratios justified the importance of interactions above the individual factors in explaining childhood stunting in Bangladesh. Superior fit to data and better predictive performance of the interaction-added model over the factors-only model further validated the significance of considering these interactions in the analysis and endorsed the need for an in-depth understanding of the inherent mechanisms between the key factors.

Despite some limitations, the strength of the study lies in the novel approach of combining an interpretable machine learning method with a classical statistical model to identify complex but significant interactions between the sociodemographic factors behind child stunting in Bangladesh and associated risk-groups. When public health interventions are concerned,

findings of this paper emphasize importance of targeting multiple risk factors that interact with each other in defining groups of children most vulnerable to stunting. This may speed-up slow reduction of childhood stunting in Bangladesh and prove worthwhile in making meaningful strides towards achieving the Government's 4th Health Nutrition and Population Sector Program (HPNSP) objective of reducing the prevalence of stunting to 25% by 2022 [3]. Importantly, the complementary methods demonstrated in this study can be applied to gain deeper socio-economic understanding of many other public health questions related to, for example, early childhood development, women's healthcare decision making, hygiene practices etc.

Acknowledgments

The authors would like to acknowledge MEASURE DHS (Demographic and Health Surveys), National Institute for Population Research and Training (NIPORT) and USAID/Bangladesh, who allowed researchers to access the survey data for free.

References

[1] World Health Organization, et al. Success factors for women's and children's health: Bangladesh. 2015;.

[2] World Health Organization. Nutrition Landscape Information System (NLIS) country profile indicators: interpretation guide. 2010;.

[3] NIPORT. Bangladesh Demographic and Health Survey 2017-18: Key Indicators. Dhaka, Bangladesh, and Rockville, Maryland, USA: NIPORT, and ICF. 2019;.

[4] De Onis M, Dewey KG, Borghi E, Onyango AW, Blössner M, Daelmans B, et al. The W orld H ealth O rganization's global target for reducing childhood stunting by 2025: rationale and proposed actions. Maternal & child nutrition. 2013;9:6–26.

[5] Dewey KG, Begum K. Long-term consequences of stunting in early life. Maternal & child nutrition. 2011;7:5–18.

[6] Prendergast AJ, Humphrey JH. The stunting syndrome in developing countries. Paediatrics and international child health. 2014;34(4):250–265.

[7] Black RE, Victora CG, Walker SP, Bhutta ZA, Christian P, De Onis M, et al. Maternal and child undernutrition and overweight in low-income and middle-income countries. The lancet. 2013;382(9890):427–451.

[8] Stewart CP, Iannotti L, Dewey KG, Michaelsen KF, Onyango AW. Contextualising complementary feeding in a broader framework for stunting prevention. Maternal & child nutrition. 2013;9:27–45.

[9] Alom J, Quddus MA, Islam MA. Nutritional status of under-five children in Bangladesh: a multilevel analysis. Journal of biosocial science. 2012;44(5):525–535.

[10] Islam MM, Alam M, Tariquzaman M, Kabir MA, Pervin R, Begum M, et al. Predictors of the number of under-five malnourished children in Bangladesh: application of the generalized poisson regression model. BMC public health. 2013;13(1):11.

[11] Fakir AM, Khan MWR. Determinants of malnutrition among urban slum children in Bangladesh. Health economics review. 2015;5(1):22.

[12] Hasan MT, Soares Magalhaes RJ, Williams GM, Mamun AA. The role of maternal education in the 15-year trajectory of malnutrition in children under 5 years of age in B angladesh. Maternal & child nutrition. 2016;12(4):929–939.

[13] Rabbani A, Khan A, Yusuf S, Adams A. Trends and determinants of inequities in childhood stunting in Bangladesh from 1996/7 to 2014. International journal for equity in health. 2016;15(1):186.

[14] Chowdhury MRK, Rahman MS, Khan MMH, Mondal MNI, Rahman MM, Billah B. Risk factors for child malnutrition in Bangladesh: a multilevel analysis of a nationwide population-based survey. The Journal of pediatrics. 2016;172:194–201.

[15] Das S, Gulshan J. Different forms of malnutrition among under five children in Bangladesh: a cross sectional study on prevalence and determinants. BMC Nutrition. 2017;3(1):1.

[16] Talukder A. Factors associated with malnutrition among under-five children: illustration using Bangladesh demographic and health survey, 2014 data. Children. 2017;4(10):88.

[17] Hossain MB, Khan MHR. Role of parental education in reduction of prevalence of childhood undernutrition in Bangladesh. Public health nutrition. 2018;21(10):1845–1854.

[18] Huda TM, Hayes A, El Arifeen S, Dibley MJ. Social determinants of inequalities in child undernutrition in Bangladesh: A decomposition analysis. Maternal & child nutrition. 2018;14(1):e12440.

[19] Saha UR, Chattapadhayay A, Richardus JH. Trends, prevalence and determinants of childhood chronic undernutrition in regional divisions of Bangladesh: Evidence from demographic health surveys, 2011 and 2014. PloS one. 2019;14(8).

[20] Sultana P, Rahman MM, Akter J. Correlates of stunting among under-five children in Bangladesh: a multilevel approach. BMC Nutrition. 2019;5(1):41.

[21] Khanam M, Shimul SN, Sarker AR. Individual-, household-, and community-level determinants of childhood undernutrition in Bangladesh. Health services research and managerial epidemiology. 2019;6:2333392819876555.

[22] Andrews DW, Whang YJ. Additive interactive regression models: circumvention of the curse of dimensionality. Econometric Theory. 1990;6(4):466–479.

[23] James G, Witten D, Hastie T, Tibshirani R. An introduction to statistical learning. vol. 112. Springer; 2013.

[24] Pawloski LR, Kitsantas P. Classification tree analysis of stunting in Malian adolescent girls. American Journal of Human Biology: The Official Journal of the Human Biology Association. 2008;20(3):285–291.

[25] Debnath A, Bhattacharjee N. Factors associated with malnutrition among tribal children in India: a non-parametric approach. Journal of tropical pediatrics. 2014;60(3):211–215.

[26] Thangamani D, Sudha P. Identification of malnutrition with use of supervised datamining techniques–decision trees and artificial neural networks. Int J Eng Comput Sci. 2014;3(September 9):8236–8241.

[27] Svefors P, Sysoev O, Ekstrom EC, Persson LA, Arifeen SE, Naved RT, et al. Relative importance of prenatal and postnatal determinants of stunting: data mining approaches to the MINIMat cohort, Bangladesh. BMJ open. 2019;9(8):e025154.

[28] United Nations. Transforming our world: The 2030 agenda for sustainable development. General Assembley 70 session. 2015;.

[29] NIPORT. Bangladesh Demographic and Health Survey 2014. Dhaka, Bangladesh, and Rockville, Maryland, USA: National Institute of Population Research and Training (NIPORT), Mitra & Associates and ICF International. 2016;.

[30] World Health Organization (WHO). Multicentre Growth Reference Study Group 2006. WHO Child Growth Standards: Length/height-for age, weight-for-age, weight for length, weight for height: Methods and development Geneva: World Health Organization. 2006;p. 312.

[31] Rutstein SO, Johnson K. The DHS wealth index. DHS comparative reports no. 6. Calverton, MD: ORC Macro. 2004;.

[32] Friedman JH, Popescu BE, et al. Predictive learning via rule ensembles. The Annals of Applied Statistics. 2008;2(3):916–954.

[33] Breiman L, Friedman J, Stone CJ, Olshen RA. Classification and regression trees. CRC press; 1984.

[34] Hothorn T, Hornik K, Zeileis A. Unbiased recursive partitioning: A conditional inference framework. Journal of Computational and Graphical statistics. 2006;15(3):651–674.

[35] Benjamin DJ, Berger JO, Johannesson M, Nosek BA, Wagenmakers EJ, Berk R, et al. Redefine statistical significance. Nature Human Behaviour. 2018;2(1):6.

[36] Hong R, Banta JE, Betancourt JA. Relationship between household wealth inequality and chronic childhood under-nutrition in Bangladesh. International journal for equity in health. 2006;5(1):15.

[37] Vollmer S, Bommer C, Krishna A, Harttgen K, Subramanian S. The association of parental education with childhood undernutrition in low-and middle-income countries: comparing the role of paternal and maternal education. International journal of epidemiology. 2017;46(1):312–323.

[38] Musa MK, Muhammad F, Lawal KM, Chowdhury A, Hossain A. Risk factors of severe acute malnutrition among under-five children: a hospital-based study in Bangladesh. J Med Sci Health. 2017;3:13–21.

[39] Naved RT. B angladesh, Families in. Encyclopedia of Family Studies. 2016;p. 1–5.

[40] Kim R, Mejia-Guevara I, Corsi DJ, Aguayo VM, Subramanian S. Relative importance of 13 correlates of child stunting in South Asia: Insights from nationally representative data from Afghanistan, Bangladesh, India, Nepal, and Pakistan. Social Science & Medicine. 2017;187:144–154.

[41] Pramod Singh G, Nair M, Grubesic RB, Connell FA. Factors associated with underweight and stunting among children in rural Terai of eastern Nepal. Asia Pacific Journal of Public Health. 2009;21(2):144–152.

[42] Rahman A, Chowdhury S. Determinants of chronic malnutrition among preschool children in Bangladesh. Journal of biosocial science. 2007;39(2):161–173.

[43] Rahman M. Association between order of birth and chronic malnutrition of children: a study of nationally representative Bangladeshi sample. Cadernos de saude publica. 2016;32:e00011215.

[44] Tödtling F, Trippl M. One size fits all?: Towards a differentiated regional innovation policy approach. Research policy. 2005;34(8):1203–1219.

[45] Ryan P, Lauver DR. The efficacy of tailored interventions. Journal of Nursing Scholarship. 2002;34(4):331–337.

[46] Schmidt EM. The effect of women's intrahousehold bargaining power on child health outcomes in Bangladesh. Undergraduate Economic Review. 2012;9(1):4.

Figure

Click here to access/download;Figure;Figure 1 - Classification Tree.eps

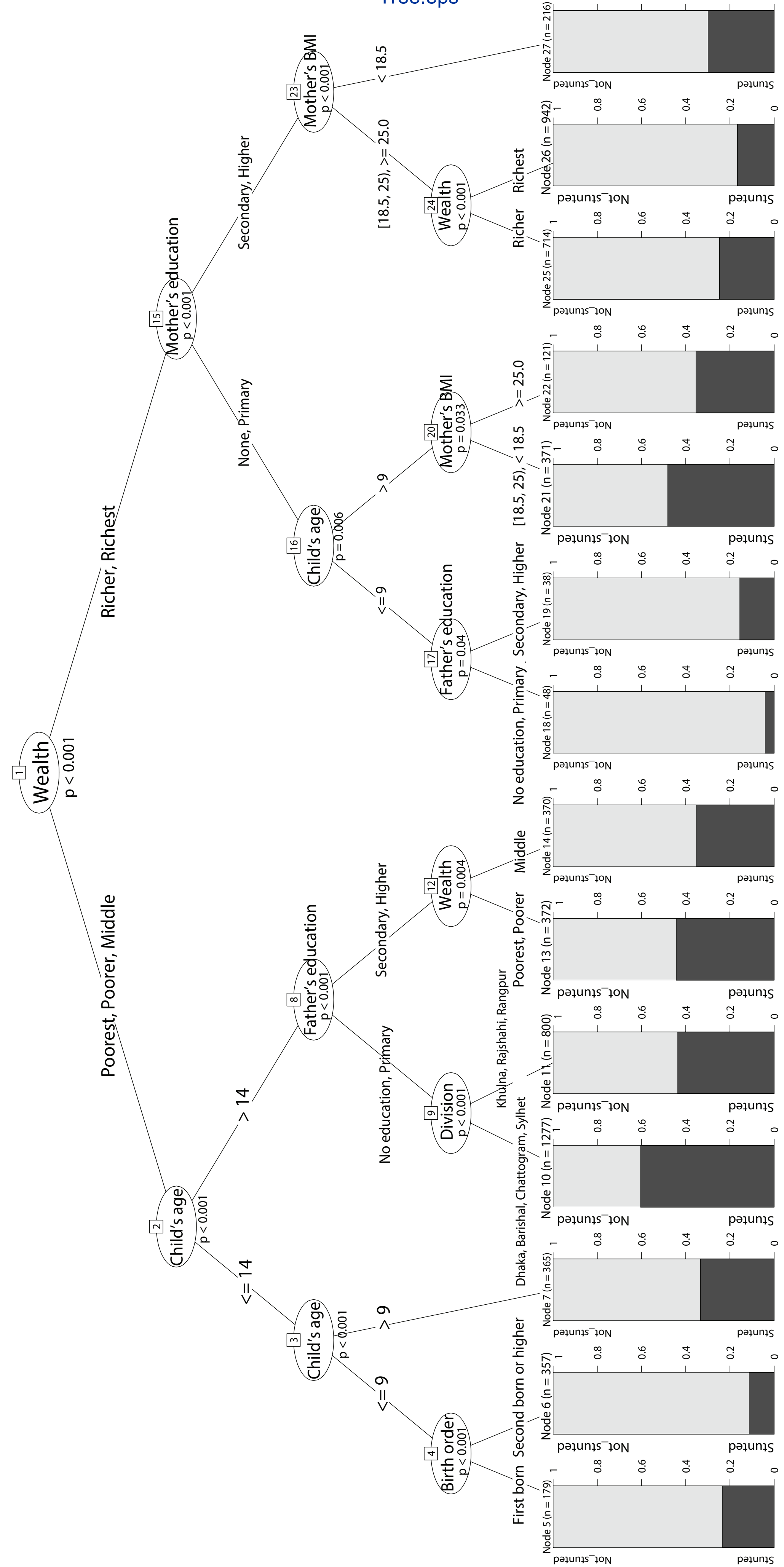

Sociodemographic Risk Factors of Under-Five Stunting in Bangladesh: Assessing the Role of Interactions Using a Machine Learning Method

Mohaimen Mansur[1*], Awan Afiaz[1], Md. Saddam Hossain[2]

1 Institute of Statistical Research and Training, University of Dhaka, Dhaka, Bangladesh
2 Population Council, Dhaka, Bangladesh

* mmansur@isrt.ac.bd

Abstract

~~Childhood stunting has been a persistent problem in Bangladesh with decades of interventions failing to reach the intended targets. Existing empirical studies investigating the problem in a socioeconomic context largely ignore the complex interplay of potential determinants that may be crucial for an intricate understanding of child malnutrition.~~This paper aims to demonstrate the importance of studying interactions among various sociodemographic risk factors of childhood stunting in Bangladesh with the help of an interpretable machine learning method. Data used for the analyses are extracted from the Bangladesh Demographic and Health Survey (BDHS) 2014 and pertain to a sample of 6,170 under-5 children. Social and economic determinants such as wealth, mother's decision making on healthcare, parental education are considered in addition to geographic divisions and common demographic characteristics of children including age, sex and birth order. A classification tree was first constructed to identify important interaction-based rules that characterize children with different profiles of risk for stunting. Then binary logistic regression models were fitted to measure the importance of these interactions along with the individual risk factors. Results revealed that, as individual factors, living in Sylhet division (OR: 1.57; CI: 1.26 - 1.96), being an urban resident (OR: 1.28; CI: 1.03 - 1.96) and having working mothers (OR: 1.21; CI: 1.02 - 1.44) were associated with higher likelihoods of childhood stunting, whereas belonging to the richest households (OR: 0.56; CI: 0.35 - 0.90), higher BMI of mothers (OR: 0.68 CI: 0.56 - 0.84) and mothers' involvement in decision making about children's healthcare with father (OR: 0.83, CI: 0.71 - 0.97) were linked to lower likelihoods of stunting. Importantly however, risk classifications defined by the interplay of multiple sociodemographic factors showed more extreme odds ratios (OR) of stunting than single factor ORs. For example, children aged 14 months or above who belong to poor wealth class, have lowly educated fathers and reside in either Dhaka, Barisal, Chittagong or Sylhet division are the most vulnerable to stunting (OR: 2.52, CI: 1.85 - 3.44). The findings endorse the need for tailored-intervention programs for children based on their distinct risk profiles and sociodemographic characteristics. ~~Such targeted efforts could prove to be cost-effective and more efficient in achieving the Health Nutrition and Population Sector Program (HNPSP) objective of limiting the prevalence of stunting in children within 25% by the year 2022 and aid in attaining the Sustainable Development Goal 2.2 of ending all forms of child malnutrition in Bangladesh~~.

Introduction

Despite many socioeconomic challenges, Bangladesh achieved remarkable successes in many areas of public health during the last two decades which include reducing child and maternal mortality to significantly low levels. [1]. However, high childhood malnutrition has remained as one of the lasting national health problems [2]. A key outcome of this nutrition deficiency is low height-for-age, commonly known as stunting, which reflects the aggregate repercussions of undernutrition in children since birth or even prenatal stages, indicating long-term restriction on a child's growth potential [3]. The current situation of childhood stunting in Bangladesh is alarming. According to the latest round of Bangladesh Demographic and Health Survey conducted in 2017, almost one-third of the under-five children fail to reach a standard height for their ages [4]. This figure is one of the highest in the world outside Sub-Saharan Africa, and largely overshadows the 12 percentage-point decline in the prevalence of stunting that has been achieved by Bangladesh over the last decade [4].

Bangladesh has done relatively well in reducing the prevalence of childhood stunting from 51% in 2004 to 31% in 2017. However, the resulting average annual rate of reduction (AARR) of 3.4% is still below the World Health Organization (WHO) recommended AARR of f 3.9% to meet the global target of a 40% reduction in the number of children in the world who are stunted by 2025 [5]. Only if the rapid reduction in the last few years can be maintained, Bangladesh will achieve its government's 4th Health Nutrition and Population Sector Program (HNPSP) objective of limiting the prevalence of stunting in children within 25% by the year 2022 [4]. The high prevalence together with the demand for continued high rate of reduction in childhood stunting presents a persistent threat to the national health status of children in Bangladesh. It is now well-known that in addition to fatal consequences like child mortality low height-for-age also has long-term health effects including chronic illness, disabilities, and cognitive impairment [6, 7]. Moreover, stunted children are likely to be physically, intellectually, and economically less productive in adulthood than well-nourished children [8, 9].

The long-standing problem of childhood stunting in Bangladesh has duly attracted substantial attention in academic research. A major interest of this vast literature lies in identifying the sociodemographic determinants of stunting, a key step for designing effective socioeconomic policy interventions targeted to reduce this public health problem. Risk factors that commonly appeared in many empirical studies include children's age, gender, birth order, underweight mothers, low levels of parent's education, and poor socioeconomic status, among others [2, 10–21]. Rarely addressed in these existing papers, however, is the possible complex interaction among different sociodemographic features of children. It is often the intricate interplay of multiple risk factors rather than individual factors that characterize potential risks of young-age undernourishment. For example, while nourishment is generally low in children whose families are poor or children whose mothers are not educated, it is probably even lower in children who are exposed to both these risk factors. From a policy perspective, this would mean that improving both education and economic conditions of the most vulnerable group may yield a faster and more efficient reduction in stunting.

The main argument of this paper stems from the fact that studies that ignore these possible interactions are likely to provide an inadequate understanding of the sociodemographic disparities driving childhood stunting and therefore, are likely to offer limited policy values. This study, therefore, aims to justify the merits of incorporating such interactions among potential driving factors of childhood stunting in Bangladesh

by using data from a nationally representative survey. A major contribution of the paper is methodological which involves utilizing a classification tree, a method borrowed from the machine learning arena, to automatically detect the complex interactions among the common risk factors of childhood stunting. Importance of these interactions are then evaluated through the use of classical logistic regression models. Interplay of multiple factors show higher association with stunting and provide deeper insight to the persistent public health problem of the country.

One of the possible reasons why interactions between variables did not feature much in the empirical public health research including nutrition studies is their predominant use of regression models, either in the form of linear or logistic. These statistical models are not best suited to capture and analyze interactions, particularly when the important ones are not known in advance. Typically, the number of possible interactions increases drastically with the number of variables incorporated in a regression model, leading to the curse of dimensionality. This eventually results in the over-parametrization of models and subsequent difficulties in estimation.

In this paper, we go against the convention and exploit the classification tree, a method borrowed from the machine learning arena, to identify important interactions capable of apprehending the complex interplay between the sociodemographic variables discussed in the literature. Classification trees scale up well to large datasets and use combinations of explanatory variables to stratify data into various risk groups. Unlike parametric regression models, this nonparametric method offers a more flexible data-driven approach to fit complex data and also offers an easy and intuitive interpretation of results.

While classification trees are being increasingly used in many areas of public health research, its applications in the field of childhood nutrition are still few. To our knowledge, only one paper used classfication trees to discover risk factors of childhood stunting in the context of Bangladesh. While the paper uses a rich set of biological, socioeconomic, and environmental information on children under two years of age, the sample data is limited only to low-income participants from a small district (smaller regions under divisions) of rural Bangladesh. Consequently, findings from the paper are not directly generalizable to the relevant child population in Bangladesh which very likely exhibits wider sociodemographic and spatial variations. In contrast, our study utilizes data from a population-based nationally-representative survey. A fundamental difference of this paper with these existing studies is that in addition to identifying interactions among sociodemographic determinants of stunting it further measures the importance and statistical significance of these interactions through applications of classical regression models.

The current analyses aim to make important methodological contributions by utilizing the classification tree to identify groups of children with various risk profiles for stunting. The importance of such risk stratification is further verified through the use of widely accepted statistical models. Importantly, this risk profiling is characterized by the interaction of multiple sociodemographic features of children allowing the possibility for targeted interventions based on their respective vulnerabilities. Considering the fact that Bangladesh is observing an undesirably slow reduction in childhood stunting, such need-specific tailored policies can speed up the progress towards achieving the Goal 2.2 of the Sustainable Development Goals (SDGs) that aims to end all forms of malnutrition including stunting in children under 5 years of age by the year 2030.

Materials and methods

Data overview

This study used data from the Bangladesh Demographic and Health Survey (BDHS) 2014. This is a nationally representative cross-sectional survey conducted in Bangladesh in collaboration with the Demographic and Health Survey (DHS) and is operated by Measure DHS+ under the authority of the National Institute of Population Research and Training (NIPORT). The survey sampling frame was based on the 2011 Bangladesh population census, and a two-stage stratified cluster sampling technique was employed in the survey. In the first stage, 600 enumeration areas (EAs), a geographic area consisting of 113 households on average, were independently selected with probability proportional size (PPS) method from all 20 sampling strata. Then using an equal probability systematic selection in the second stage, 30 households were selected from each cluster (EA). The survey resulted in 17,886 completed interviews with ever-married women between the ages of 15 to 49. Further details of the sampling procedure can be found in [22].

In addition to a number of health and nutrition relation information, the BDHS survey also collected measurements on height and weight from eligible women of reproductive ages and children under the age of 5 years. Complete and credible anthropometric and age data on 7,318 children were collected in the process [22]. After excluding the temporary (de facto) residents, children of twin births and any cases with missing values in the variables, the final sample size for this study was 6,170.

Ethics statement

This article does not contain any studies with human participants performed by any of the authors. The Bangladesh demographic and health Surveys were approved by ICF Macro Institutional Review Board and the National Research Ethics Committee of the Bangladesh Medical Research Council. A written consent about the survey was given by participants before the interview. All identification of the respondents was dis-identified before publishing data. The secondary data sets analysed during the current study are freely available upon request from the DHS website at `https://dhsprogram.com/data/available-datasets.cfm`. Searching 'Bangladesh DHS, 2014' in the DHS website will provide the survey data set.

Outcome variable

~~Anthropometric measurements of height and weight were used to assess the nutrition status of Bangladeshi children under 5 years of age. The BDHS 2014 data constructed several indices from these measurements according to the WHO Child Growth Standards, such as stunting, wasting, and underweight, that takes into account the variations in ethnicity, social and economic influences, and feeding practices worldwide. These indices provide important information about a child's growth and body composition and act as key indicators in order to assess their nutritional status.~~

The outcome variable of this study concerns the height-for-age or stunting index which reflects the cumulative effect of constant malnourishment as well as recurrent and chronic illnesses resulting in lack of growth among children compared to their respective ages. Two standard deviations below the median (-2 SD) of the WHO reference population in terms of height-for-age indicates that the child is short for his or her age, i.e. stunted [23]. This variable was pre-calculated according to the definition provided by WHO in the BDHS 2014 child dataset.

Independent variables

Based on the literature review and pre-analysis, the current study explored the variables that commonly appeared in many nutrition related studies on Bangladesh. At the child level, these included the age of children in months (0-59 months), sex (male, female), as well as the birth order (first born, second born, and third born or above) of the child and the decision-maker regarding child's healthcare needs (mother only, both parents, and husband and/or someone else). Independent variables related to the mother such as mother's age at the birth of her first child (in years), number of living children (one, two, and three or more children), body mass index (BMI) level (less than 18.5, between 18.5 and 25, and greater than 25), education level (no education, primary, secondary, higher secondary or above), current employment status (currently working, not working) and mother's age difference to the father (in years) along with father's education level (no education, primary, secondary, higher secondary or above) were analyzed. It is important to mention that the 'no education' category for both parents' education implies 'no formal education.

The sociodemographic variables, related to the child's household characteristics, were also explored for association with stunting in children under 5 years of age, which included household's wealth index (poorest, poorer, middle, richer, richest), area of residence (urban, rural) and administrative division (Dhaka, Barishal, Chattogram, Khulna, Rajshahi, Rangpur, Sylhet). The wealth index quintiles were pre-calculated using principal component analysis on household assets [24] and were readily available in the dataset.

Methodology

The analyses of the paper were carried out in two steps using a machine learning approach and a classical regression model in succession. In the first step of the analysis, a classification tree was fit to the data to identify possible interactions among predictors of childhood stunting. In the second step, the interactions identified by the trees are incorporated in a logistic regression framework in addition to individual explanatory variables, and their impact on the likelihood of stunting measured. This two-step method can be thought to be a simpler version of the *Rulefit* approach proposed in [25] where an ensemble of trees rather than a single tree is used to identify important interaction rules which are then included in a penalized regression model.

Machine learning is largely viewed as data-driven methods that can automatically detect patterns from data and use those uncovered patterns to predict future or unseen data or to make decisions under uncertainty [26]. An important member of this group, a classification tree is a popular machine learning technique. It divides the sample data into small subgroups based on simple rules involving the predictors. The rules originate from step-wise binary splits of the predictors where the most important predictor at each step (referred to as a node) is selected for splitting. The tree is produced with an aim to achieving minimum error in classification (e.g., whether a child is stunted or not) for subgroups produced at each node. A classification tree is nonparametric in nature and holds a number of advantages over parametric regression type models including minimal distributional assumptions and easier interpretation. The most important advantage, however, is the tree's inherent capability of capturing complex, such as non-linear, interactions of variables in high dimensional settings [27]. Classical regression models are not best suited to capture and analyze interactions, particularly when the important ones are not known in advance. Typically, the number of possible interactions increases drastically with the number of variables incorporated in a

regression model, leading to the curse of dimensionality [28]. This eventually results in the over-parametrization of models and subsequent difficulties in estimation.

The standard classification tree techniques such as CART [29] are often criticized for biased selection of variables which have many possible splits and missing values. These problems have been rectified in a conditional inference tree framework proposed in [30] that has been adopted in the current analyses. An inherent limitation of a classification tree in general, however, is that when it is grown indefinitely, node sample sizes become smaller and the tree overfits data, meaning that it fits the noise more than the signal in the data [27]. In this analysis, the depth of the tree was fixed to four layers to keep a balance between capturing adequate complexity and avoid too low (less than 50) sample size per node.

While trees offer flexible approaches to reveal interactions among explanatory variables from the sample data, it does not provide measurable sizes of effects of these interactions on the response (childhood stunting) or their statistical significance. To achieve these, interactions/rules were incorporated in a logistic regression setting as additional predictors. Rules yielding either a stunting prevalence of greater than 40% or less than 20% were considered suggestive of significantly high and low prevalence, respectively, and considered for entering into the regression model. These thresholds for rule selection were constructed based around the national prevalence of under-five stunting of 36% in Bangladesh [22].

Each selected rule is then converted to a binary variable taking a value of 1 when the defining criteria for that particular rule are met, and a value of 0, otherwise. Each of these binary variables is, thus, defined by unique interactions of several sociodemographic features of children. Next, two logistic regression models were estimated and compared. In the first model, only the initially selected independent variables were fitted to the stunting outcome (stunted, not stunted) and no interaction rule was considered. But in the second model, binary variables from selected rules were included along with the individual variables. Survey weights are duly applied in both the classification tree and logistic regression models for making the sample results representative of the population of interest. Findings from regression models are further adjusted for the complexity of the survey design to capture cluster- and strata-wise variations and appropriately compute the standard errors and p-values for regression estimates.

Finally, classification accuracies of the two logistic regression models are systematically evaluated for both in-sample goodness of fit and out-of-sample predictive performance. Comparisons are made by computing standard criteria such as accuracy, sensitivity, specificity and the area under the Receiver Operating Characteristic Curve (ROC). The first three criteria are calculated based on a probability threshold of 0.5. To obtain the area under the curve (AUC) for the out-of-sample ROCs, a 10-fold cross-validation approach was undertaken and the mean AUC values were reported in the findings.

To ensure the reproducibility in scientific research in cases of new discoveries, the present study followed the recommendation by [31] in identifying the significant interaction rules. That is, the proposed interaction terms will be considered significant if the corresponding p-values were less than 0.005 in the second model. All analyses were performed in R *(version 3.6.0)*. The binary logistic regression models were fitted using the *"survey"* package *(version 4.0)* and the classification tree was constructed

using the *"partykit"* package *(version 1.2-7)*.

Results

Descriptive statistics

Table 1 presents the distribution of stunting in under-five children by their sociodemographic characteristics considered in the study. Although the age of the children was considered as a continuous numeric variable in later analysis, it was converted into a categorical variable for exploratory reasons in this table. The overall stunting prevalence was 36.5% in the study data. The results reveal that children aged two years or older were more stunted (41.8%) than younger children (28.3%). Children of higher birth order were also stunted in higher proportions (42.7%) than the first or second-born children.

Table 1. Distribution of stunting by sociodemographic variables (categorical and continuous) among children aged 0-59 months

Variables	Groups	N (%) of study participants	N (%) of stunted
**Age of child (in months)**	0-23 months	2432 (39.4)	689 (28.3)
	23-59 months	3738 (60.6)	1563 (41.8)
**Sex of child**	Male	3167 (51.3)	1175 (37.1)
	Female	3003 (48.7)	1077 (35.9)
**Child's birth order**	First born	2260 (36.6)	730 (32.3)
	Second born	1885 (30.6)	657 (34.9)
	Third born or above	2025 (32.8)	865 (42.7)
**Decision-maker regarding child's healthcare**	Mother only	948 (15.4)	319 (33.6)
	Both parents	3493 (56.6)	1252 (35.8)
	Husband or somebody else	1729 (28.0)	681 (39.4)
**Number of children alive to mother**	One child	2060 (33.4)	636 (30.9)
	Two children	2122 (34.4)	756 (35.6)
	Three or more	1988 (32.2)	860 (43.3)
**Mother's BMI**	Less than 18.5	1387 (22.5)	633 (45.6)
	Between 18.5 - 25.0	3619 (58.7)	1330 (36.8)
	25 or above	1164 (18.9)	289 (24.8)
**Mother's education level**	No education	981 (15.9)	491 (50.1)
	Primary	1727 (28.0)	770 (44.6)
	Secondary	2835 (45.9)	866 (30.5)
	Higher	627 (10.2)	125 (19.9)
**Father's education level**	No education	1564 (25.3)	773 (49.4)
	Primary	1881 (30.5)	767 (40.8)

Table 1. Distribution of stunting by sociodemographic variables (categorical and continuous) among children aged 0-59 months *(continued)*

Variables	Groups	N (%) of study participants	N (%) of stunted
	Secondary	1854 (30.0)	541 (29.2)
	Higher	871 (14.1)	171 (19.6)
**Mother's employment status**	Not working	4577 (74.2)	1617 (35.3)
	Currently working	1593 (25.8)	635 (39.9)
**Household wealth index**	Poorest	1359 (22.0)	697 (51.3)
	Poorer	1159 (18.8)	476 (41.1)
	Middle	1202 (19.5)	450 (37.4)
	Richer	1256 (20.4)	394 (31.4)
	Richest	1194 (19.4)	235 (19.7)
**Area of residence**	Rural	4229 (68.5)	1623 (38.4)
	Urban	1941 (31.5)	629 (32.4)
**Administrative division**	Dhaka	1081 (17.5)	366 (33.9)
	Barisal	725 (11.8)	278 (38.3)
	Chattogram	1156 (18.7)	431 (37.3)
	Khulna	687 (11.1)	190 (27.7)
	Rajshahi	756 (12.3)	227 (30.0)
	Rangpur	770 (12.5)	272 (35.3)
	Sylhet	995 (16.1)	488 (49.0)
**Age of mother at first birth (mean[SD])**		18.2 [3.22]	17.7 [3.02]
**Mother's age difference to father (median)**		8.0	7.0
**Total sample size**	N	6170	2252 (36.5)

The mean age of mothers at their first birth was 18.2 years with a standard deviation of 3.23 years. Fathers were mostly older than mothers with a median age difference of 8 years. Children belonging to mothers with three or more living children were more stunted (43.3%) than those households where they were the only child (30.9%) or had only one sibling (35.6%). The prevalence of stunting is also lower (33.6%) in cases where the mother was the sole decision-maker regarding the child's healthcare needs in comparison to cases where decisions are made by the husband or another member of the family (39.4%). The prevalence of stunting for children belonging to underweight mothers was more than 20% higher than that in children born to mothers who were overweight (24.8%). Reduced percentages of stunting were observed for higher levels of educational attainment of both mother and father. Working mothers had a higher percentage of stunted children (39.9%). A deeper look into the data showed that the working-mother sample has almost a 9 percentage-point higher proportion of poor (poorest or poorer) individuals and a 5 percentage-point higher proportion of individuals with no formal education when compared to the non-working group. Furthermore, while the proportion of mothers with higher than secondary level education is only 1-percentage point higher in the working group, proportion of secondary-completed education is about 7-percentage point lower. The proportion of stunted children was also high at the lowest wealth quintile (51.3%) but decreased with higher levels of wealth. Percentage stunted in rural households (38.4%) was higher than the national average of 36% while that in urban households was below

(32.4%). The prevalence of stunting was the highest in the Sylhet division (49%) and the lowest in the Khulna division (27.7%). Further breakdown of data revealed that the Khulna division had the lowest percentage of illiterate (not formally educated) mothers (8.3%) and one of the lowest proportions of poorest mothers (18.9%). In contrast, the proportions of uneducated and poorest mothers are among the highest (27.9% and 30.3%, respectively) in Sylhet. These facts may explain part of the observed glaring variation between stunting percentages in the two divisions.

Classification tree

The classification tree in **Figure 1** shows the interplay of important sociodemographic factors behind stunting in children under five years of age. The wealth reveals itself to be the most important predictor being placed at the top of the tree. Children belonging to middle or lower categories of wealth statuses are placed under the left branch. The age of a child is the next most important variable in this branch showing children younger than 14 months to be less vulnerable to stunting. Other noteworthy predictors on this side of the tree were father's education, administrative division, and the birth order of the child. In this case, children older than 14 months with lowly educated fathers living in Dhaka, Barishal, Chattogram, and Sylhet divisions (node 10) seemed to be at the highest risk (around 60%) of being stunted.

Fig 1. Classification tree for stunting in under-five children in Bangladesh
The value of n at each final node gives the node sample size and the black fill in the box below indicates proportion of stunted children under the node. The p-value at each node corresponds to a unified permutation test of hypothesis indicating independence between the response and predictors [30].

The right-hand branch originated from the root of the classification tree include the wealthier households. On this side, mother's education, and then their body mass index (BMI) as well as the age of a child were notable predictors of stunting. Here, children who have mothers with no/low formal education, who are older than 9 months and have relatively lower BMI (node 21) demonstrate high risk of child stunting (arond 48%). From the classification tree, we chose six interaction rules defined by six nodes (6, 10, 11, 13, 21, and 26, respectively) to be important for our analysis. The previously stated national prevalence of stunting of 36% has been utilized to determine the thresholds for these rule selections. A node was considered to be of significant importance if it showed the prevalence of stunting to be higher than 40% (high risk) or less than 20% (low risk). However, we excluded nodes 18 and 19 from further consideration on account of having considerably low sample sizes (below 50) which make reasonable statistical inference for relevant population difficult and are probably indicative of overfitting data in respective categories. The selected six nodes were then transformed into binary variables based on whether the observations satisfied all four classification criteria under each node, displayed in **Table 2**, and are named as "Rule- 6, 10, 11, 13, 21, and 26" respectively.

Table 2. Interaction rules extracted from the classification tree and associated stunting

Rule ID	Rule combinations	Node frequency	Stunting prevalence
**Rule 6**	Wealth index = Poorest, Poorer, Middle & Age of child <= 14 & Age of child <= 9 & Birth order = Second born, Third born or above	357	11.50%
**Rule 10**	Wealth index = Poorest, Poorer, Middle & Age of child > 14 & Father's education level = No education, Primary & Division= Dhaka, Barisal, Chittagong, Sylhet	1277	60.50%
**Rule 11**	Wealth index = Poorest, Poorer, Middle & Age of child > 14 & Father's education level = No education, Primary & Division= Khulna, Rajshahi, Rangpur	800	43.90%
**Rule 13**	Wealth index = Poorest, Poorer, Middle & Age of child > 14 & Father's education level = Secondary, Higher & Wealth index = Poorest, Poorer	372	44.40%
**Rule 21**	Wealth index = Richer, Richest & Mother's education level = No education, Primary & Age of child > 9 & Mother's BMI = Less than 18.5, Between 18.5 to 25	371	48.20%
**Rule 26**	Wealth index = Richer, Richest & Mother's education level = Secondary, Higher & Mother's BMI = Between 18.5 to 25 , Greater than 25 & Wealth index = Richest	942	16.70%

Logistic regression models

Results from the logistic regressions are presented in **Table 3**. The first model which included only the sociodemographic variables in the study, exhibits that stunting was positively associated with the age of a child (OR 1.02 with 95% CI: 1.015, 1.024), corroborating the findings from the descriptive statistics. The likelihood of stunting reduced by nearly 20% when the mother was the sole decision-maker of the child's healthcare needs (OR 0.81 with 95% CI: 0.66, 0.99) or when both parents jointly made such decisions (OR 0.83 with 95% CI: 0.71, 0.97) as opposed to the father or somebody else being in charge of such decision-making. The odds of stunting reduced by 39% (p-value $< .001$) for mother's with a BMI greater than 25, in comparison to mothers with a BMI score between 18.5 and 25. Father's education also played a part with both secondary (OR 0.73 with 95% CI: 0.58, 0.92) and higher (OR 0.54 with 95% CI: 0.39, 0.75) categories showing a significantly lower likelihood of stunting compared to fathers with no education.

Table 3. Odds ratios (OR) and associated confidence intervals (CI) and p-values (p) from the two logistic regression models.

	Model 1: No interaction			Model 2: With interaction rules		
**Variables**	**OR**	**95% CI**	**p**	**OR**	**95% CI**	**p**
**Age of child (in months)**	1.02	1.015, 1.024	<0.001	1.00	1.00, 1.01	0.094
**Sex of child**						
Male	1.00			1.00		
Female	0.97	0.84, 1.12	0.666	0.95	0.83, 1.09	0.489
**Child's birth order**						
First born	1.00			1.00		
Second born	1.08	0.81, 1.45	0.597	1.06	0.78, 1.44	0.698
Third born or above	1.02	0.64, 1.61	0.941	1.11	0.70, 1.75	0.663
**Decision-maker regarding child's healthcare**						
Husband or somebody else	1.00			1.00		
Mother only	0.81	0.66, 0.99	0.040	0.82	0.66, 1.01	0.068
Both parents	0.83	0.71, 0.97	0.019	0.83	0.71, 0.97	0.021
**Age of mother at 1st birth**	0.98	0.95, 1.01	0.177	0.98	0.95, 1.01	0.236
**Number of children alive to mother**						
One child	1.00			1.00		
Two children	1.18	0.87, 1.58	0.288	1.34	0.98, 1.82	0.067
Three or more	1.09	0.68, 1.75	0.722	1.14	0.71, 1.81	0.591
**Mother's BMI**						
18.5 - 25.0	1.00			1.00		
Less than 18.5	1.15	0.91, 1.45	0.237	1.11	0.88, 1.41	0.367
25 or above	0.61	0.50, 0.75	<0.001	0.68	0.56, 0.84	<0.001
**Mother's education level**						
No education	1.00			1.00		
Primary	1.09	0.81, 1.46	0.565	1.05	0.78, 1.41	0.760
Secondary	0.89	0.66, 1.19	0.430	1.03	0.77, 1.38	0.847
Higher	0.99	0.64, 1.54	0.973	1.18	0.77, 1.83	0.449
**Father's education level**						
No education	1.00			1.00		
Primary	0.88	0.74, 1.04	0.124	0.90	0.75, 1.08	0.256
Secondary	0.73	0.58, 0.92	0.007	0.92	0.71, 1.20	0.545
Higher	0.54	0.39, 0.75	<0.001	0.72	0.51, 1.03	0.070
**Mother's employment status**						
Not working	1.00			1.00		
Currently working	1.21	1.01, 1.44	0.034	1.21	1.02, 1.44	0.032

Table 3. Odds ratios (OR) and associated confidence intervals (CI) and p-values (p) from the two logistic regression models. *(continued)*

	Model 1: No interaction			Model 2: With interaction rules		
**Variables**	**OR**	**95% CI**	**p**	**OR**	**95% CI**	**p**
**Mother's age difference to father**	0.99	0.98, 1.00	0.084	0.99	0.97, 1.00	0.069
**Household wealth index**						
Poorest	1.00			1.00		
Poorer	0.86	0.71, 1.04	0.117	0.83	0.68, 1.01	0.061
Middle	0.72	0.59, 0.89	0.002	0.77	0.62, 0.95	0.017
Richer	0.58	0.45, 0.75	<0.001	0.65	0.49, 0.87	0.004
Richest	0.36	0.26, 0.49	<0.001	0.56	0.35, 0.90	0.016
**Area of residence**						
Rural	1.00			1.00		
Urban	1.29	1.04, 1.60	0.020	1.28	1.03, 1.59	0.028
**Administrative division**						
Dhaka	1.00			1.00		
Barisal	1.11	0.85, 1.44	0.438	1.11	0.85, 1.46	0.433
Chittagong	1.20	0.97, 1.47	0.092	1.20	0.97, 1.48	0.099
Khulna	0.67	0.53, 0.86	0.002	0.80	0.61, 1.05	0.114
Rajshahi	0.65	0.52, 0.82	<0.001	0.82	0.64, 1.05	0.122
Rangpur	0.88	0.68, 1.14	0.338	1.07	0.81, 1.42	0.632
Sylhet	1.51	1.23, 1.86	<0.001	1.57	1.26, 1.96	<0.001
**Rules extracted from the classification tree**						
**rule6**				0.22	0.15, 0.33	<0.001
**rule10**				2.52	1.85, 3.44	<0.001
**rule11**				1.57	1.19, 2.07	0.001
**rule13**				1.64	1.15, 2.33	0.006
**rule21**				2.01	1.41, 2.86	<0.001
**rule26**				0.65	0.43, 0.99	0.047

Interestingly, having a working mother is associated with 21% higher odds of stunting (p-value: 0.034) when compared to mothers who were not employed at the time of the survey. Children from middle, rich and, the richest households were 28%, 42%, and 64% less at risk of stunting compared to children from poorest households (p-value <.005). Furthermore, children from urban areas were significantly more likely (OR 1.29 with 95% CI: 1.04, 1.60) to be stunted than children in rural areas. Children living in Sylhet division had 51% higher risk of being stunted (p-value <.001) compared to Dhaka, whereas the burden of risk was more than 30% lower for children living in Khulna (OR 0.67 with 95% CI: 0.53, 0.86) and Rajshahi (OR 0.65 with 95% CI: 0.52, 0.82).

The second model, where the interaction rules extracted from the classification tree were included along with the sociodemographic variables, showed somewhat different results. Age of children, the mother being the sole decision-maker of child's healthcare, father's education level and administrative divisions (barring Sylhet) were no longer associated with stunting individually at 5% level of significance. However, the rest of the significant associations remained as they were with minor changes in the odds of

either increasing or decreasing the likelihood of stunting.

Following the suggestion by [31], the interaction rules 6, 10, 11, and 21 were significantly associated with stunting at 0.5% level of significance. The children corresponding to rule-6, indicating those who live in the poorest, poorer or middle-wealth households, who are younger than 9 months and who rank second or higher in the birth order, were 78% less at risk of stunting than those who do not belong to this rule. According to rule-10, odds of stunting in children of lower wealth categories who are more than 14 months old, have primary or uneducated fathers and live in any one of Dhaka, Barisal, Chittagong, Sylhet divisions was 2.5 times the odds in the children who do not fall into these categories. In contrast, according to rule-11, children with same wealth, age and paternal education levels as in rule-10, exhibit an odds ratio of only 1.54 when they resided in any one of the other three divisions - Khulna, Rajshahi, or Rangpur. It is also worth noting that, children aged greater than 14 months who belonged to the poorest or poorer household but have secondary or higher educated fathers were also at 64% higher odds of stunting (rule-13 with p-value 0.006). Finally, based on rule-21, children aged greater than 9 months who belonged to the richer or richest households with mothers having little or no formal education and a BMI of 25 or less were twice as likely to be stunted as children who have different other combinations of risk factors.

Model validation

The predictive performances of both logistic regression models were evaluated to assess whether the addition of decision tree rules as interactions in the second model played any part in improving the results. For out of sample predictions a 10-fold cross-validation procedure was followed. **Table 4** shows that, when fitting in-sample data is concerned, including the decision tree rules in the model yielded a more than 18% increase in the sensitivity at the cost of a marginal drop in specificity (2.35%). Similarly, in the out-of-sample predictions, sensitivity improved by 22% with a slight drop in specificity. In both scenarios, larger areas under the ROC curve (AUC) further confirmed the benefit from adding the interaction-induced rules in the model.

Table 4. Comparison of model performance

Performance type	Sensitivity	Specificity	Accuracy	AUC
**In-sample (full sample fit)**				
Model 1: Without interaction	0.366	0.847	0.671	0.692
Model 2: With interaction	0.440	0.832	0.689	0.715
**Out-of-sample (10-fold cross validation)**				
Model 1: Without interaction	0.360	0.850	0.674	0.694
Model 2: With interaction	0.436	0.824	0.684	0.712

Sensitivity, specificity and accuracy are computed based on a probability threshold of 0.5. Results for out-of-sample performances are averages across 10-fold cross-validations. AUC is the area under the Receiver Operating Characteristic (ROC) curve.

Discussion

The present analysis assessed the role of interactions between important sociodemographic factors, commonly identified in the literature, in explaining childhood stunting. The findings from the two-step analysis provided several interesting perspectives. In the exploratory part of the analysis, the classification tree identified the

specific groups of under-five children with various levels of risk for stunting. Essentially, each of these different risk profiles was characterized by a unique combination of several predictively important sociodemographic features and provided noteworthy insights into the phenomenon of childhood stunting. These insights stemming from the complex interplay of multiple factors is not directly observable when looking at the pertinent factors individually. While the importance of these interactions is well perceived by the nutrition experts, supporting empirical evidences are largely undocumented.

For example, the distribution of stunted children by household wealth quintile reported in **Table 1** shows that the percentage of stunted children in households belonging to the richer and richest wealth statuses were 31.4% and 19.7%, respectively. However, node 21 of the classification tree in **Figure 1** (Rule 21 in Table 2) revealed that a sub-group of children from these wealth classes who were above 9 months of age and born to illiterate or lowly educated (primary education) mothers having low to moderate BMI (BMI < 25), have a much higher (48%) prevalence of stunting. Note that, the use of an interpretable machine learning method has divulged insights with more depth than what would normally be achieved.

Similarly, vulnerable groups with analogous risk profiles could be characterized by very different combinations of risk factors with the help of classification trees. Evidently, children under node 11 of the classification tree (Rule 11 in Table 2) had only 4% lower risk of stunting than those in the aforementioned node 21, but possess noticeably dissimilar features concerning household wealth and parental education.

While classification trees are being increasingly used in many areas of public health research recently [32, 33], its applications in the field of childhood nutrition are still few [34–36]. To our knowledge, only one paper [37] used classfication trees to discover risk factors of childhood stunting in the context of Bangladesh. While the paper uses a rich set of biological, socioeconomic, and environmental information on children under two years of age, the sample data is limited only to low-income participants from a small district (smaller regions under divisions) of rural Bangladesh. Consequently, findings from the paper are not directly generalizable to the relevant child population in Bangladesh which very likely exhibits wider sociodemographic and spatial variations. We take advantage of this limitation by exploiting national-level survey data.

Findings from the classification tree of our analysis corroborate what we already know about childhood stunting in developing countries including Bangladesh while providing us with fresh perspectives. Sociodemographic variables that appeared in the tree have been largely identified as significant predictors of child growth in the literature as well as in the factor-only logistic regression model of this study (Model 1 of Table 3). The prevalence of adverse conditions and food insecurity in underprivileged households is well-documented and so is the association between poverty and stunting [20, 38]. Mother's educational attainment is an important factor for a child's nourishment [13, 39] as this corresponds to women's knowledge regarding mothering a child and their subsequent care-taking. Paternal education has been also found to be relevant [17, 40] as it is linked to increased earning and health awareness, particularly in low- and middle-income households, and complements maternal education in many cases [41].

A mother's BMI is representative of her own physical attributes, stature and nourishment. It is, therefore, not surprising that we found children having low-BMI mothers to be at higher risk of stunting. This finding is well-documented in the literature [42, 43]. Age and birth order are two other established demographic risk

factors of childhood stunting. Naturally, differences in height become more prominent as children grow off their infancy. For birth order, research found that higher order children are often subject to shorter duration of breastfeeding, lower prenatal and postnatal investments and lower care-time from parents [44]. These probably explain the association of higher age and higher birth order with higher level of stunting - a result we found and also confirmed by existing research [45, 46]. Another aspect of mother that was significant in our regression analysis was her current employment status. It may seem unusual that children of working mothers appeared to be more likely to be stunted. However, as reported in the *Results* section, higher percentages of both poor and illiterate individuals in the working-mother sample may partly explain this association. However, the cross-sectional nature of the data do not allow us to venture further into the probable causes.

An interesting finding of this study is that children whose healthcare decisions are made either by the mother alone or jointly by both parents have significantly lower odds of stunting than children whose decisions are made solely by the father or someone else. This could also be a key learning in the understanding of the sociodemographic mechanism behind childhood stunting in Bangladesh and is in line with findings in the literature [47]. Individually considered, all of the discussed factors are undoubtedly important for explaining child malnutrition. However, the interplay between these factors offered a deeper understanding of the sociodemographic effect on childhood stunting which is likely to be crucial in designing targeted intervention strategies.

In the confirmatory part of the analysis, selected groups of children with low and high risks of stunting, as identified by the tree, were incorporated into a logistic regression model (Model 2 in Table 3) together with the individual risk factors and were investigated for their possible associations with stunting. Here, all but one association were statistically significant at least at 1% level of significance. More importantly, the strength of associations of these interaction-induced risk groups with stunting, measured by odds ratio (OR), were predominantly higher (for positive associations) or lower (for negative associations) than those for individual risk factors.

As an example, the highest odds ratio for an individual variable was 1.57, observed for the Sylhet division, implying that living in this division increased the likelihood of stunting by 57% compared to the residents in the Dhaka division. In contrast, the odds ratios for positively associated rules are in the range of 1.57 to 2.52, the highest corresponding to rule 10 reported in Table 2 and implying that the odds of stunting for children living in certain divisions including Sylhet and possessing specific risk features was 2.5 times the odds for children having other characteristics. Similarly, among the individual predictors, children belonging to the richest household wealth quintile were associated with the lowest odds ratio (OR) of 0.56, while the OR for a negatively associated rule was as low as 0.22 (rule 6 in Table 2).

The combination of results from the classification tree and the logistic regression may bear valuable implications on research and policies related to stunting in under-five children. Identification of vulnerable groups and specific interactions of multiple sociodemographic factors that define these groups can aid in expanding the understanding of different aspects of stunting. Policymakers working towards the reduction of stunting may utilize this intricate knowledge in designing risk-specific tailored interventions by targeting the characterizing features.

One policy implication based on the significance of wealth-education-division

interaction will pertain to identifying the poorest and lowly educated families living in high-risk divisions such as Sylhet, Barishal or Chattogram Dhaka via small-scale surveys. These are the groups most vulnerable to childhood stunting and measures should be taken to increase post-primary education and employment among them. These can be achieved by offering free or cheap higher education to these families and by creating low-capital income-generating opportunities. Based on the findings of high prevalence of stunting in relatively low-BMI and lowly educated but rich mothers, a second set of intervention programs may be formulated to actively promote maternal and child health-nutrition literacy via television and social media platforms, which are commonly accessible to the target group of potential wealthy mothers. Such targeted interventions can be quicker and more effective, but less expensive in achieving faster reduction in stunting as opposed to a nation-wide implementation of a more general policy such as making tertiary female education free of cost in the entire country. Importance of such tailored interventions are emphasized in disciplines like innovation studies [48] and nursing care [49].

While the focus of the paper is the evaluation of interaction among sociodemographic characteristics of children as determinants of stunting, a by-product of the findings of the analysis is that children whose healthcare decisions are made either by the mother alone or jointly by both parents have significantly lower odds of stunting than children whose decisions are made solely by the father or someone else. This could also be a key learning in the understanding of the sociodemographic mechanism behind childhood stunting in Bangladesh and is in line with findings in the literature. Intervention programs aimed at increasing the practice of granting mothers' autonomy in child's healthcare decision-making or encouraging mothers' inclusion in such decisions could go a long way in achieving the goal of alleviating this problem. Furthermore, interventions programs that already include educating mothers regarding child's health and nourishment could also be positively impacted by the inclusion of mothers in such decision-making practices.

The present study has a few limitations. Firstly, this study investigated only height-for-age (stunting) in children and ignored weight-for-age (overweight) and weight-for-height (wasting) - the other two widely recognized anthropometric indicators of childhood malnutrition. While a complete understanding of the drivers of childhood undernutrition will require involving all three aspects, the choice of looking at one is driven by the limited space for a single study. Secondly, the secondary data was obtained from a cross-sectional survey as opposed to a longitudinal study and so no causal relationship between the factors and the outcome could be established. Thirdly, the selection of explanatory variables is limited to those that generally appeared significant in major empirical public health studies rather than all possible inclusions from an exhaustive search of the literature. Finally, the tree depth was limited to four layers for avoiding too low sample size per node and two nodes were excluded from analysis as a result even after exceeding the pre-specified thresholds.

Conclusion

High prevalence of childhood stunting and low rate of reduction per year imply that Bangladesh is falling behind in meeting national and international targets for reducing early-age undernutrition. This paper emphasizes the importance of studying the possible interplay of different sociodemographic determinants of child stunting, largely missing from existing literature, for a deeper insight into the problem. With that aim, analyses adopted in the paper exploit a classification tree to uncover

complex interactions from data and incorporate them in a logistic regression model. Significantly high and low magnitudes of odds ratios justified the importance of interactions above the individual factors in explaining childhood stunting in Bangladesh. Superior fit to data and better predictive performance of the interaction-added model over the factors-only model further validated the significance of considering these interactions in the analysis and endorsed the need for an in-depth understanding of the inherent mechanisms between the key factors.

The aim of this paper is to assess the importance of interactions among sociodemographic risk factors of childhood stunting in Bangladesh which have been largely overlooked in the existing literature. Despite some limitations, the strength of the study lies in the novel approach of combining an interpretable machine learning method with a classical statistical model to identify these complex interactions and associated risk-groups. While wealth, area and division of residence, mother's BMI and father's education were among the important individual risk factors of stunting, certain interactions among many of these factors proved to be significantly more important. For a simple example, the combination of low wealth and low education of fathers seem to put children at higher risks of poor growth than when the two factors are considered in isolation.

When social interventions are concerned, findings of this paper emphasize importance of targeting multiple socio-economic risk factors that interact with each other in defining groups of children most vulnerable to stunting. For example, the wealth-education interaction may imply that rather than just providing safety nets (e.g., cash transfers) to poor and lowly educated pregnant mothers implementing a joint program providing them also with free education on maternal and child's health and nutrition may be more effective in reducing severe childhood stunting at later ages. At government and private policy making, these tailored policies targeting high-risk socio-economic groups may further speed-up the reduction of childhood stunting in Bangladesh. Importantly, the complementary methods demonstrated in this study can be applied to gain deeper socio-economic understanding of many other public health questions related to, for example, early childhood development, women's healthcare decision making and hygiene practices.

Supporting information

S1 Fig. Classification tree for stunting in under-five children in Bangladesh.

Acknowledgments

The authors would like to acknowledge MEASURE DHS (Demographic and Health Surveys), National Institute for Population Research and Training (NIPORT) and USAID/Bangladesh, who allowed researchers to access the survey data for free.

References

1. World Health Organization, et al. Success factors for women's and children's health: Bangladesh. 2015;.

2. Rabbani A, Khan A, Yusuf S, Adams A. Trends and determinants of inequities in childhood stunting in Bangladesh from 1996/7 to 2014. International journal for equity in health. 2016;15(1):186.

3. World Health Organization. Nutrition Landscape Information System (NLIS) country profile indicators: interpretation guide. 2010;.

4. NIPORT. Bangladesh Demographic and Health Survey 2017-18: Key Indicators. Dhaka, Bangladesh, and Rockville, Maryland, USA: NIPORT, and ICF. 2019;.

5. De Onis M, Dewey KG, Borghi E, Onyango AW, Blössner M, Daelmans B, et al. The W orld H ealth O rganization's global target for reducing childhood stunting by 2025: rationale and proposed actions. Maternal & child nutrition. 2013;9:6–26.

6. Dewey KG, Begum K. Long-term consequences of stunting in early life. Maternal & child nutrition. 2011;7:5–18.

7. Prendergast AJ, Humphrey JH. The stunting syndrome in developing countries. Paediatrics and international child health. 2014;34(4):250–265.

8. Black RE, Victora CG, Walker SP, Bhutta ZA, Christian P, De Onis M, et al. Maternal and child undernutrition and overweight in low-income and middle-income countries. The lancet. 2013;382(9890):427–451.

9. Stewart CP, Iannotti L, Dewey KG, Michaelsen KF, Onyango AW. Contextualising complementary feeding in a broader framework for stunting prevention. Maternal & child nutrition. 2013;9:27–45.

10. Alom J, Quddus MA, Islam MA. Nutritional status of under-five children in Bangladesh: a multilevel analysis. Journal of biosocial science. 2012;44(5):525–535.

11. Islam MM, Alam M, Tariquzaman M, Kabir MA, Pervin R, Begum M, et al. Predictors of the number of under-five malnourished children in Bangladesh: application of the generalized poisson regression model. BMC public health. 2013;13(1):11.

12. Fakir AM, Khan MWR. Determinants of malnutrition among urban slum children in Bangladesh. Health economics review. 2015;5(1):22.

13. Hasan MT, Soares Magalhaes RJ, Williams GM, Mamun AA. The role of maternal education in the 15-year trajectory of malnutrition in children under 5 years of age in B angladesh. Maternal & child nutrition. 2016;12(4):929–939.

14. Chowdhury MRK, Rahman MS, Khan MMH, Mondal MNI, Rahman MM, Billah B. Risk factors for child malnutrition in Bangladesh: a multilevel analysis of a nationwide population-based survey. The Journal of pediatrics. 2016;172:194–201.

15. Das S, Gulshan J. Different forms of malnutrition among under five children in Bangladesh: a cross sectional study on prevalence and determinants. BMC Nutrition. 2017;3(1):1.

16. Talukder A. Factors associated with malnutrition among under-five children: illustration using Bangladesh demographic and health survey, 2014 data. Children. 2017;4(10):88.

17. Hossain MB, Khan MHR. Role of parental education in reduction of prevalence of childhood undernutrition in Bangladesh. Public health nutrition. 2018;21(10):1845–1854.

18. Huda TM, Hayes A, El Arifeen S, Dibley MJ. Social determinants of inequalities in child undernutrition in Bangladesh: A decomposition analysis. Maternal & child nutrition. 2018;14(1):e12440.

19. Saha UR, Chattapadhayay A, Richardus JH. Trends, prevalence and determinants of childhood chronic undernutrition in regional divisions of Bangladesh: Evidence from demographic health surveys, 2011 and 2014. PloS one. 2019;14(8).

20. Sultana P, Rahman MM, Akter J. Correlates of stunting among under-five children in Bangladesh: a multilevel approach. BMC Nutrition. 2019;5(1):41.

21. Khanam M, Shimul SN, Sarker AR. Individual-, household-, and community-level determinants of childhood undernutrition in Bangladesh. Health services research and managerial epidemiology. 2019;6:2333392819876555.

22. NIPORT. Bangladesh Demographic and Health Survey 2014. Dhaka, Bangladesh, and Rockville, Maryland, USA: National Institute of Population Research and Training (NIPORT), Mitra & Associates and ICF International. 2016;.

23. World Health Organization (WHO). Multicentre Growth Reference Study Group 2006. WHO Child Growth Standards: Length/height-for age, weight-for-age, weight for length, weight for height: Methods and development Geneva: World Health Organization. 2006;p. 312.

24. Rutstein SO, Johnson K. The DHS wealth index. DHS comparative reports no. 6. Calverton, MD: ORC Macro. 2004;.

25. Friedman JH, Popescu BE, et al. Predictive learning via rule ensembles. The Annals of Applied Statistics. 2008;2(3):916–954.

26. Murphy KP. Machine Learning: A Probabilistic Perspective. The MIT Press; 2012.

27. James G, Witten D, Hastie T, Tibshirani R. An introduction to statistical learning. vol. 112. Springer; 2013.

28. Andrews DW, Whang YJ. Additive interactive regression models: circumvention of the curse of dimensionality. Econometric Theory. 1990;6(4):466–479.

29. Breiman L, Friedman J, Stone CJ, Olshen RA. Classification and regression trees. CRC press; 1984.

30. Hothorn T, Hornik K, Zeileis A. Unbiased recursive partitioning: A conditional inference framework. Journal of Computational and Graphical statistics. 2006;15(3):651–674.

31. Benjamin DJ, Berger JO, Johannesson M, Nosek BA, Wagenmakers EJ, Berk R, et al. Redefine statistical significance. Nature Human Behaviour. 2018;2(1):6.

32. Afiaz A, Masud MS, Mansur M. Impact of child?s cognitive and social-emotional difficulties on child abuse: Does mother?s justification of intimate partner violence also play a role? Child Abuse & Neglect. 2021;117:105028.

33. Fox H, Topp SM, Lindsay D, Callander E. A cascade of interventions: A classification tree analysis of the determinants of primary cesareans in Australian public hospitals. Birth. 2021;.

34. Pawloski LR, Kitsantas P. Classification tree analysis of stunting in Malian adolescent girls. American Journal of Human Biology: The Official Journal of the Human Biology Association. 2008;20(3):285–291.

35. Debnath A, Bhattacharjee N. Factors associated with malnutrition among tribal children in India: a non-parametric approach. Journal of tropical pediatrics. 2014;60(3):211–215.

36. Thangamani D, Sudha P. Identification of malnutrition with use of supervised datamining techniques–decision trees and artificial neural networks. Int J Eng Comput Sci. 2014;3(September 9):8236–8241.

37. Svefors P, Sysoev O, Ekstrom EC, Persson LA, Arifeen SE, Naved RT, et al. Relative importance of prenatal and postnatal determinants of stunting: data mining approaches to the MINIMat cohort, Bangladesh. BMJ open. 2019;9(8):e025154.

38. Hong R, Banta JE, Betancourt JA. Relationship between household wealth inequality and chronic childhood under-nutrition in Bangladesh. International journal for equity in health. 2006;5(1):15.

39. Vollmer S, Bommer C, Krishna A, Harttgen K, Subramanian S. The association of parental education with childhood undernutrition in low-and middle-income countries: comparing the role of paternal and maternal education. International journal of epidemiology. 2017;46(1):312–323.

40. Musa MK, Muhammad F, Lawal KM, Chowdhury A, Hossain A. Risk factors of severe acute malnutrition among under-five children: a hospital-based study in Bangladesh. J Med Sci Health. 2017;3:13–21.

41. Naved RT. B angladesh, Families in. Encyclopedia of Family Studies. 2016;p. 1–5.

42. Kim R, Mejia-Guevara I, Corsi DJ, Aguayo VM, Subramanian S. Relative importance of 13 correlates of child stunting in South Asia: Insights from nationally representative data from Afghanistan, Bangladesh, India, Nepal, and Pakistan. Social Science & Medicine. 2017;187:144–154.

43. Pramod Singh G, Nair M, Grubesic RB, Connell FA. Factors associated with underweight and stunting among children in rural Terai of eastern Nepal. Asia Pacific Journal of Public Health. 2009;21(2):144–152.

44. Buckles K, Kolka S. Prenatal investments, breastfeeding, and birth order. Social Science & Medicine. 2014;118:66–70.

45. Rahman A, Chowdhury S. Determinants of chronic malnutrition among preschool children in Bangladesh. Journal of biosocial science. 2007;39(2):161–173.

46. Rahman M. Association between order of birth and chronic malnutrition of children: a study of nationally representative Bangladeshi sample. Cadernos de saude publica. 2016;32:e00011215.

47. Schmidt EM. The effect of women's intrahousehold bargaining power on child health outcomes in Bangladesh. Undergraduate Economic Review. 2012;9(1):4.

48. Tödtling F, Trippl M. One size fits all?: Towards a differentiated regional innovation policy approach. Research policy. 2005;34(8):1203–1219.

49. Ryan P, Lauver DR. The efficacy of tailored interventions. Journal of Nursing Scholarship. 2002;34(4):331–337.

June 15, 2021

Srinivas Goli, Ph.D.

Academic Editor

PLOS ONE

RE: Revision of Manuscript (REF: PONE-D-20-31337.R1)

Dear Editor,

Thank you for providing us with the opportunity to further revise and improve our manuscript titled "*Sociodemographic Risk Factors of Under-Five Stunting in Bangladesh: Assessing the Role of Interactions Using a Machine Learning Method*".

We have addressed the reviewers' recommendations in a point by point basis in our response below. We have incorporated the required changes into the manuscript through careful revisions as prescribed by the reviewers and provided a copy of the manuscript indicating the track changes as well. For convenience, the reviewers' comments are **highlighted in blue** and our response is written in black.

All the required changes made are marked in red in the revised manuscript and deleted texts are marked in blue. In addition, some minor changes throughout the revised version of the manuscript were made, mostly to fix some grammatical issues and improve the clarity of expressions.

Please convey our most sincere gratitude to the reviewers for their valuable feedback and suggestions in order to improve the manuscript.

We cordially thank you for your patience and kind consideration, especially during these unprecedented times. Please stay safe.

Sincere regards,

The authors

We would like to thank the reviewers for their valuable comments, which have led to an improved version of the paper. The corrections that have been made in response to the comments are provided are as below. All of the suggested corrections are marked in red, while notable deletions are marked in blue strike-through.

Reviewer #1:

“Sociodemographic Risk Factors of Under-Five Stunting in Bangladesh: Assessing the Role of Interactions Using a Machine Learning Method” by Mansur M et al. is a well written manuscript with thorough analytical presentation on a very important aspect of childhood malnutrition, under 5-stunting. Childhood stunting is an important indicator of malnutrition which is prevalent in most developing countries, including Bangladesh. In spite of remarkable successes in several health indices, the rate of reduction of childhood stunting in Bangladesh is much behind expectation. The authors have tried to find out the potential driving factors of childhood stunting in Bangladesh by studying interactions among various sociodemographic risk factors with the help of an interpretable machine learning method using data from a nationally representative survey¸ Bangladesh Demographic and Health Survey 2014. Their anticipation is that by tailored-intervention programs for children based on their distinct risk profiles and sociodemographic characteristics will help to achieve the national Health Nutrition and Population Sector Program objective of limiting the prevalence of stunting in children within 25% by the year 2022 and Goal 2.2 of the SDGs to end all forms of malnutrition including stunting in children under 5 years of age by the year 2030.

The manuscript is well written, yet I have few observations as mentioned below:

The ‘Introduction’ looks unnecessarily lengthy. The first three paragraphs (Lines 2-47) are fine as the introduction for the article. In the last 4 paragraphs (Lines 49-88) the authors have explained how they identified the important interactions capable of apprehending the complex interplay between the common sociodemographic variables by utilizing the classification tree to identify groups of children with various risk profiles for stunting and then further verified it through the use of widely accepted statistical models. They have also explained its superiority over the predominant use of regression models, either in the form of linear or logistic, used in previous literatures.

The methods, authors used for the analyses, are elaborately described in ‘Methodology’ (Lines 146-206), So, such details can easily be avoided in ‘Introduction’. It would be better if the authors briefly describe their methods here before the concluding sentence of previous paragraph (Lines 45-47) and comparison of their method with the methods used in previous studies briefly in ‘Discussion’.

Authors’ response:

We thank the reviewer for this detailed observation and apt suggestion. We have made the required changes in the ‘Introduction’ accordingly. Comparisons of results derived from the machine learning method in this study and those in literature are duly moved in the ‘Discussion’ section in the revised manuscript. All changes are marked in red.

The prediction in lines 15-18, “Furthermore, the chronological drop in the prevalence of stunting from 51% in 2004 to 31% in 2017 translates to an average annual rate of reduction (AARR) of 3.4% which falls short of the global AARR of 3.9% required to attain World Health Organization's target to reduce by 40% the global prevalence of stunting by 2025”, might not be that straightforward. The reduction, like improvements in other health indices, probably was much faster in recent years.

Authors’ response:

We thank the reviewer for this prudent observation. Indeed, while the overall AARR since 2004 is lower than required, the rate of reduction was indeed much faster in recent years. In order to clarify the problem better, we have now revised the quoted statement, and in the following statement posed the problem as ‘*demand for continued rapid reduction*’ rather than previously stated ‘slow rate of reduction’ [lines 23-26 in the revised manuscript].

It would be better to delete the sentence “Some of the recent studies are [9, 10, 11, 12, 13, 14, 15, 16, 17, 18, 19, 20, 21]” in Lines 33-34 and give the citations “9-21” after the previous sentence.

Authors’ response:

Thank you. The required changes have been made following the reviewer’s suggestion.

Childhood stunting (height-for-age) is an important, but not the only predictor of malnutrition; many stunting cases are either due to familial or constitutional. The anthropometric methods for measuring the nutritional status includes three widely used indicators to assess the growth of children: height-for-age, weight-for-height, and weight-for-age. Adding other parameters could strengthen the understanding of the objective of the study. It is obvious that in a single study it might not be possible to include all aspects, but at least can be mentioned as a limitation.

Authors' response:

We certainly agree with the reviewer's comment. We have now included this point as a limitation of the study in the 'Discussion' section. In particular, we have added the following sentence [lines 518-523 in the revised manuscript]:
"*Firstly, this study investigated only height-for-age (stunting) in children and ignored weight-for-age (overweight) and weight-for-height (wasting) - the other two widely recognized anthropometric indicators of childhood malnutrition. While a complete understanding of the drivers of childhood undernutrition will require involving all three aspects, the choice of looking at one is driven by the limited space for a single study.*"

I am not sure why the authors have not used data from Bangladesh Demographic and Health Survey 2017.

We started this study almost a year ago with an aim to apply our methods to the most recent data. Unfortunately, Bangladesh Demographic and Survey (BDHS) 2017 data were not available then and were released only on December 23, 2020 – almost three months after we have submitted our manuscript.
https://datacatalog.worldbank.org/dataset/bangladesh-demographic-and-health-survey-2017-2018
Furthermore, we would like to stress that the main objective of our study is to show the usefulness of the proposed machine learning assisted logistic regression method in capturing interactions among socio-demographic risk factors of childhood stunting, rather than provide a thorough critical analysis of current stunting situation in Bangladesh. We believe that the results of our study using BDHS 2014 data will not change much qualitatively if the recently released 2017 data were used. Moreover, the plethora of extant literature based on the 2014

BDHS dataset also plays to the advantage in the sense that the present analyses discussed in this paper is supported by sufficient literature and thereby indirectly validates the premise of the results obtained by our new methodological approach. However, we plan to investigate the possible temporal changes in a future study.

Reviewer #2:

Overall the manuscript is fine. The literature review has successfully addressed the objective of the research. The manuscript presented a nice application of machine learning method in public health research (data analysis) comparing with typical logistical model which is innovative idea. However, for a reader without having any prior knowledge of machine learning could be difficult to recognize the method.

No major issues for revision, but some minor issues to be addressed are as follows:

a) Line 225-226: "Working mothers' had a higher percentage of stunted children (39.9%)"-Are these working mothers from lower class? Have no education? Are they working in their own area and staying with their children or they migrate to other cities leaving children at home. Need a bit explanation otherwise it looks more generalized data.

Authors' response:

We thank the reviewer for the comment and enquiry. We added the following information about those working mothers following the quoted statement [lines 275-281].

"*A deeper look into the data showed that the working-mother sample has almost 9 percentage point higher proportion of poor (poorest or poorer) individuals and 5 percentage point higher proportion of individuals with no formal education when compared to the non-working group. Furthermore, while the proportion of mothers with higher than secondary level education is only 1-percentage point higher in the working group, proportion of secondary-completed education is about 7-percentage point lower.*"

The detailed cross-table results are avoided in the revised article but provided below for the reviewer's consideration. However, we would like to clarify that other information on mothers' workplace such as possible migration status or area of work were not available in the dataset and thus not discussed in the paper.

Working Status	Wealth Index					Row Total
	Poorest	Poorer	Middle	Richer	Richest	
**Not working**	957	806	868	960	986	4577
(Row %), (Col %)	20.9, 70.4	17.6, 69.5	19.0, 72.2	21.0, 76.4	21.5, 82.6	74.2
**Working**	402	353	334	296	208	1593
(Row %), (Col %)	25.2, 29.6	22.2, 30.5	21.0, 27.8	18.6, 23.6	13.1, 17.4	25.8
P-value	<0.001					

Working Status	Mother's Education				Row Total
	No education	Primary	Secondary	Higher	
**Not working**	672	1267	2183	455	4577
(Row %), (Col %)	14.7, 68.5	27.7, 73.4	47.7, 77.0	9.9, 72.6	74.2
**Working**	309	460	652	172	1593
(Row %), (Col %)	19.4, 31.5	28.9, 26.6	40.9, 23.0	10.8, 27.4	25.8
**P-value**	<0.001				

b) Line 229: The paper mentioned two specific areas (Sylhet and Khulna) of Bangladesh where the prevalence of stunning children were highest and lowest. Would you please explain which indicators were specifically worked for this variation between two areas?

Authors' response:

While we believe that it is difficult to identify the true indicators working behind the glaring variation between stunting percentages in the two divisions (Sylhet and Khulna) without a systematic investigation involving many potential risk factors, a quick look from our data showed that the Khulna division had the lowest percentage of illiterate (not formally educated) mothers (8.3%) and one of the lowest proportions of poorest mothers (18.9%). In contrast, the proportions of uneducated and poorest mothers are among the highest (27.9% and 30.3%, respectively) in Sylhet. These facts may explain part of the observed variation.

We added the detailed information in the text following the sentence [lines 286-291] and also provide the result below (not added in the manuscript) for the consideration of reviewer.

**Division**	**Mother's Education**				**Row Total**
	**No education**	**Primary**	**Secondary**	**Higher**	
**Dhaka**	183	313	454	131	1081
(Row %), (Col %)	16.9, 18.7	29.0, 18.1	42.0, 16.0	12.1, 20.9	17.5
**Barisal**	74	244	320	87	725
(Row %), (Col %)	10.2, 7.5	33.7, 14.1	44.1, 11.3	12.0, 13.9	11.8
**Chattogram**	161	284	618	93	1156
(Row %), (Col %)	13.9, 16.4	24.6, 16.4	53.5, 21.8	8.0, 14.8	18.7
**Khulna**	57	161	381	88	687
(Row %), (Col %)	8.3, 5.8	23.4, 9.3	55.5, 13.4	12.8, 14.0	11.1
**Rajshahi**	99	190	382	85	756
(Row %), (Col %)	13.1, 10.1	25.1, 11.0	50.5, 13.5	11.2, 13.6	12.3
**Rangpur**	129	200	349	92	770
(Row %), (Col %)	16.8, 13.1	26.0, 11.6	45.3, 12.3	11.9, 14.7	12.5
**Sylhet**	278	335	331	51	995

(Row %), (Col %)	27.9, 28.3	33.7, 19.4	33.3, 11.7	5.1, 8.1	16.1
P-value	<0.001				

Division	Wealth Index					Row Total
	Poorest	Poorer	Middle	Richer	Richest	
**Dhaka**	161	153	148	261	358	1081
(Row %), (Col %)	14.9, 11.8	14.2. 13.2	13.7, 12.3	24.1, 20.8	33.1, 30.0	17.5
**Barisal**	170	206	157	115	77	725
(Row %), (Col %)	23.4, 12.5	28.4, 17.8	21.7, 13.1	15.9, 9.2	10.6, 6.4	11.8
**Chattogram**	171	172	254	292	267	1156
(Row %), (Col %)	14.8, 12.6	14.9, 14.8	22.0, 21.1	25.3, 23.2	23.1, 22.4	18.7
**Khulna**	130	120	157	155	125	687
(Row %), (Col %)	18.9, 9.6	17.5, 10.4	22.9, 13.1	22.6, 12.3	18.2, 10.5	11.1
**Rajshahi**	177	151	171	146	111	756
(Row %), (Col %)	23.4, 13.0	20.0, 13.0	22.6, 14.2	19.3, 11.6	14.7, 9.3	12.3
**Rangpur**	249	182	142	118	79	770
(Row %), (Col %)	32.3, 18.3	23.6, 15.7	18.4, 11.8	15.3, 9.4	10.3, 6.6	12.5
**Sylhet**	301	175	173	169	177	995
(Row %), (Col %)	30.3, 22.1	17.6, 15.1	17.4, 14.4	17.0, 13.5	17.4, 14.8	16.1
P-value	<0.001					

c) Line 235: Figure 1, there is no figure attached.

Authors' response:

Following PLOS ONE's submission guidelines the figure has been attached as a separate file which should be accessible to the reviewers.

d) In table 3, "Rules extracted from the classification tree" is a bit confusing. Is it a part logistical regression output?? If the objective of this table is to compare or find similarities then it can be placed in separate table.

Authors' response:

We apologize for the confusion that arose from the reported "*Rules extracted from the classification tree*" in table 3. To clarify, these are binary variables representing interactions detected in the classification tree and are part of the second logistic regression model. They did not enter the first 'No interaction' model, but entered the second logistic regression model as additional explanatory variables. We chose to present them in the same table in order to make a clear comparison between the two (with and without the rules/interactions) logistic regression models which we believe would be easier and beneficial to the readers.

e) Line 277: It would be better to understand when comparing two models, i.e. machine learning and logistic model in separate section

Authors' response:

We want to clarify that the model mentioned in line 277 is actually a logistic regression model which incorporated interactions from the machine learning method (classification tree) as additional explanatory variables. This interaction-augmented logistic regression model was then compared with a plain logistic regression model without interactions.

In this study the machine learning method is not presented as a contrasting method for logistic regression, but as a tool for effectively extracting complex interactions from data and then as a complementary method to develop more sophisticated interaction-induced logistic regression models which we posit would be an important addition to such analyses.

Other comments: Last but not the least, a simple definition with reference of Machine Learning method will be helpful for readers.

Authors' response:

This is an important suggestion and we have now included a simple definition of Machine Learning in the 'Methodology' section with reference [lines 185-188].

"Machine learning is largely viewed as data-driven methods that can automatically detect patterns from data and use those uncovered patterns to predict future or unseen data or to make decisions under uncertainty [26]. An important member of this group, a classification tree is a popular machine learning technique. It divides the sample data into small subgroups based on simple rules involving the predictors."

Reviewer #3:

Introduction:

Line :22: Height for age?

Authors' response:

We apologize for the unintentional typographical mistake which has now been corrected.

Discussion:

Please set table 4 probably mistakenly set in discussion section to the result segment

Authors' response:

We have now moved Table 4 to the Results section following the reviewer's advice.

Conclusion:

Please make it brief addressing objective

Line 419: Can be deleted as it is not a part of objective

Authors' response:

We thank the reviewer for this advice. We have now made the conclusion more concise to address specifically the objectives and deleted less relevant texts. All changes are marked in red in the revised manuscript.

Reviewer #4:

The article titled 'Sociodemographic Risk Factors of Under-Five Stunting in Bangladesh: Assessing the Role of Interactions Using a Machine Learning Method' highlighted an important public health issues in Bangladesh. I have following review comments for the authors:

a. Share few key findings in Abstract, may few numbers for the readers who does not want to read the whole article, currently it is missing.

Authors' response:

We thank the reviewer for this valuable advice and updated the abstract by adding key findings. The relevant changes are marked in red in the revised manuscript with track changes as follows:

"*Results revealed that, as individual factors, living in Sylhet division (OR: 1.57; CI: 1.26 - 1.96), being an urban resident (OR: 1.28; CI: 1.03 - 1.96) and having working mothers (OR: 1.21; CI: 1.02 - 1.44) were associated with higher likelihoods of childhood stunting, whereas belonging to the richest households (OR: 0.56; CI: 0.35 - 0.90), higher BMI of mothers (OR: 0.68 CI: 0.56 - 0.84) and mothers' involvement in decision making about children's healthcare with father (OR: 0.83, CI: 0.71 - 0.97) were linked to lower likelihoods of stunting. Importantly however, risk classifications defined by the interplay of multiple sociodemographic factors showed more extreme odds ratios (OR) of stunting than single factor ORs. For example, children aged 14 months or above who belong to poor wealth class, have lowly educated fathers and reside in either Dhaka, Barisal, Chittagong or Sylhet division are the most vulnerable to stunting (OR: 2.52, CI: 1.85 - 3.44).*"

b. 2nd sentence in Introduction, please rephrase, it sounds like stunting remained as the only public health problem in Bangladesh is high childhood malnutrition

Authors' response:

We apologize for the unintentional misleading sentence and replaced it with the following:

"However, high childhood malnutrition has remained as one of the lasting national health problems." [lines 4-5]
The change is marked in red in the revised manuscript (with track changes).

c. Line 33-34 in Introduction section… Some of the recent studies are..it looks like an incomplete sentence, consider including these references in previous sentence.

Authors' response:

We thank the reviewer for pointing out this mistake which we have duly corrected in the revised version of the manuscript [lines 36-38].

d. 2.2 Response variable, sound little new. Is there any reason not to use Outcome variable following the norm?

Authors' response:

We have now changed the term 'Response variable' to commonly used 'Outcome variable'. The two terms are sometimes used interchangeably in statistics which is the source of the confusion.

e. Conclusion in discussion section: whole conclusion section needed to be modified, currently, it sounds like an abstract of an article, sentence starting from background, methods, justification of classification tree, strengths etc..…. I would add key findings and then continue with the what is there in later part of the second paragraph.

Authors' response:

We thank the reviewer for this valuable advice. The conclusion is now modified accordingly in the revised manuscript and have been marked in the red in the track changes version.

The nodes shown in Figure 1: Although apparent criticism of the standard classification tree related to biased selection of variables supposed to be overcome by conditional inference tree

framework as mentioned in the methodology section; the presented may have some inherent limitation and pose internal validity of the method: for example, mother belonged to richer and richest arm, both mother and father had no education, but their children had lowest stunting!! Stringer justification needed.

Authors' response:

We appreciate the reviewer's observation in pointing out of the inherent limitation of classification tree. While we clearly stated in 'Classification tree' subsection of the 'Results' section that we discarded two tree nodes (the above-mentioned rule is one of them) on account of having low sample sizes (less than 50), we admit that we did not clarify the justification for these exclusions which we have included now. The main argument is that nodes with lower sample sizes (resulting from overgrown trees) often overfit the data under that node and are not generalizable to relevant population. This is indeed a common criticism of trees and we now also add this limitation in the methodology section. In particular we wrote [lines 207-210]:

"An inherent limitation of a classification tree in general, however, is that when it is grown indefinitely, node sample sizes become smaller and the tree overfits data meaning that it fits the noise more than the signal in the data [27]".

Thus, while children coming from richer and richest household and having lowly educated parents may have the lowest stunting, there are only 48 such children in the sample of over 6000 children. It is not utterly surprising to think that the risk from low education of both parents overwhelmingly outweighed the possible gain from higher wealth for this very few children, but we should not consider this as generalizable to population of such children due to small sample size. All the sample sizes for the nodes we selected for regression are over 300.

Reviewer #5:

The manuscript titled "Sociodemographic Risk Factors of Under-Five Stunting in Bangladesh: Assessing the Role of Interactions Using a Machine Learning Method" is an excellent work at the current situation of Bangladesh. This analysis of Bangladesh Demographic and Health Survey (DHS) data describes a number of different factors associated with stunting using exception method of identification. However, there are some observations from my side stated

below;

1. Abstract: It is surprising that none of the factors associated with stunting that were identified in the regression analysis and classification tree are mentioned in the abstract. It would be helpful to mention some of the most common risk factors in the abstract.

Authors' response:

We thank the reviewer for bringing this important observation to our attention and we apologize for missing the key predictors of stunting in the abstract. We have now included them with associated odds ratios from the logistic regression analysis. The changes are marked in red in the revised manuscript.

2. Page 3, line 4: Sentence not clear, Is there any evidence (prevalence over time) that support the statement?

Authors' response:

We have now added a reference as support for the mentioned statement in the revised manuscript [line 4].

3. Page 3 line 5: Stunting might be the "outcome" of nutrition deficiency rather than the "measure".

Authors' response:

We thank the reviewer for pointing out this mistake. We have now made the correction accordingly in the revised manuscript.

4. Line 93: Is there any specific reason behind using the 2014 DHS dataset? Since 2014 situation has been changed as GoB and NGOs took much strategies for child and maternal nutrition improvement targeting SDG goals.

Authors' response:

We started this study almost a year ago with an aim to apply our methods to the most recent data. Unfortunately, Bangladesh Demographic and Survey (BDHS) 2017 data were not available then and were released only on December 23, 2020 – almost three months after we have submitted our manuscript.
https://datacatalog.worldbank.org/dataset/bangladesh-demographic-and-health-survey-2017-2018
While prevalence of under-five stunting reduced from 36% in 2014 to 31% in 2017 (possible following drives from GoB and NGOs as mentioned by the reviewer) according the latest BDHS report, as long as socio-demographic drivers of childhood stunting are concerned, we do not expect our results/finding to change qualitatively if the newly released data were used. The identified risk factors were historically associated with under-five stunting in Bangladesh. However, we keep investigating any temporal changes between the last two survey periods as potential future research of interest.

Also, we would like to stress that the main objective of our study is to show the usefulness of the proposed machine learning method in capturing interactions among socio-demographic risk factors of childhood stunting, rather than to provide a thorough critical analysis of the current stunting situation in Bangladesh. Moreover, the plethora of extant literature based on the 2014 BDHS dataset also plays to the advantage in the sense that the present analyses discussed in this paper is supported by sufficient literature and thereby indirectly validates the premise of the results obtained by our new methodological approach.

5. Line 128: Initial and exclusive breast feeding, mode of delivery, child vaccination status, complementary feeding etc. are recognized as the indicators of children's growth in many studies. Can this be included as the independent variable upon data?

Authors' response:

We understand the reviewer's rightful concern for not including the above-mentioned nutrition-related variables. Our justifications for not considering the factors are as follows:

First, we found selecting the possible set of explanatory variables for childhood stunting in our study particularly challenging. Existing studies did not help much in guiding to a common or universally accepted set of indicators, and vary substantially in terms of the number and types

of variables that range from demographic and socio-economic factors to spatial and health-nutrition related factors. The main aim of our paper is to better understand the role of socio-demographic risk factors of childhood stunting through their interactions identified through a machine learning method as opposed to identify and establish a comprehensive set of best predictors of stunting. Therefore, rather than considering all possible risk factors of childhood stunting (which are numerous), in this study we focused mainly on socio-demographic determinants of under-5 stunting and other control variables which commonly appeared significantly important in many relevant empirical studies, particularly those related to Bangladesh. For this reason, we were selective and did not consider the mentioned indicators, as well as many other likely determinants of childhood stunting such as number of visits by antenatal care provider and their qualifications, hygiene practices etc. We have listed this confined (as opposed to exhaustive) selection of explanatory variables as a primary limitation of our study in the 'Discussion' section.

Second, while reviewing the literature we found that many of our considered socio-demographic factors are closely related to the mentioned health and nutrition related indicators. For example, household wealth and/or mother's education has been consistently found to be associated with complementary feeding (e.g., Dhami et al., 2019, Nkoka et al., 2018), breastfeeding practices (e.g., Rahman et al., 2020), child vaccination (e.g., Sarker et al, 2019) and choice of mode of delivery (e.g., Yaya et al., 2019) and possibly capture some of their association with stunting indirectly.

Third, since vaccination, breastfeeding and complementary feeding requirements depend on age of children, investigating impact of these factors require age-stratified analysis which is beyond the scope of this study mainly looking at interactions of common socio-demographic factors on under-5 children as a whole.

Lastly, the interactions that would be considered in a joint sociodemographic-nutritional variables setting would be too large and require a more detailed approach. We believe such a study would require more in-depth data and analyses which would be out of the scope of this study. As mentioned previously, the main idea behind the study is to introduce a methodological contribution to the extant literature with the help of a machine learning method that is easily interpretable and allows the possibility to consider complex interactions between the variables largely ignored in typical public health studies.

We, however, understand the importance of looking at nutritional factors and plan to investigate the role of these factors and their interactions in a similar setting in a future study. We hope the reviewer would agree with our line of argument presented above.

6. Line 221: By categorizing children age in to 4 to 5 groups with around 12 months interval may provide insights to observe the stunting situation among children of different age groups in regression analysis. In many studies from the similar strata found that higher prevalence of stunting prevailed among children aged 36 to 47 months (Akram R, Mostafa Kamal SM, Gaire S, Darteh EKM)

Authors' response:

We thank the reviewer for this comment. We ran the regression (model with no-interaction) with children's age as a suggested categorical variable (please see the results below) which verify the reviewer's point: children aged 35-47 months are at the highest risk of stunting. While this is an interesting result showing possible non-linearity in age-stunting relationship we defend our treatment of age as continuous variables.

First, the classification tree works by optimally choosing at each step the most important variable and a binary cut-off point for that variable which maximizes discrimination between the binary response values. It is also capable of capturing non-linear association between the explanatory variable and the outcome by splitting on the same variable multiple times in subsequent steps. The main reason why we did not categorize children's age and treated it as a continuous variable in our analysis is that we wanted to allow the classification tree to automatically choose the optimal cut-off ages which will maximize discrimination between stunted and non-stunted children. Categorizing age into predefined groups would have hindered this flexibility. If there were any significantly important non-linearity in age-stunting relationship we expected it to appear in our tree, which it unfortunately did not happen. And, to keep the entire analysis consistent we treated age also as a continuous variable in the logistic regression models. Nonetheless, we understand that such subtle non-linearity may be missed if the size of the tree is kept relatively small.

Second, the age-categorized regression did not really alter our results. The coefficients (apart from age) of the explanatory variables in the two regressions (age: categorical vs continuous)

change only slightly. But importantly, the broad finding remains the same. Children of higher ages are more or less at higher risks of stunting when compared to infants (please see the classification tree in Figure 1). While the age-specific variation in stunting prevalence is interesting, we believe that it will not prove to be of high significance in addressing the aim of the paper which is exploring interaction among explanatory variables.

Last, the use age as a continuous variable in months rather than a categorical variable with 12-month categories actually allows a more parsimonious model to be had with fewer number of parameters estimated and also permits the interpolation of results within the range of 0-59 months.

**Variables**	**Odds Ratio**	**95% CI**	**p-value**
Age of child (categorized in years)			
[0,11]	1.00		
(11,23]	3.36	2.50, 4.51	<0.001
(23,35]	3.87	2.81, 5.32	<0.001
(35,47]	4.67	3.59, 6.08	<0.001
(47,59]	3.22	2.36, 4.40	<0.001
Sex of child			
Male	1.00		
Female	0.96	0.84, 1.11	0.614
Child’s birth order			
1	1.00		
2	1.00	0.74, 1.36	0.981
More than 2	0.95	0.60, 1.51	0.831
Decision maker regarding child's healthcare			
Husband or Else	1.00		
Mother	0.81	0.65, 0.99	0.045
Parents	0.83	0.71, 0.97	0.018
Age of mother at first birth (in years)	0.98	0.95, 1.01	0.282
No of children alive to mother			

1	1.00		
2	1.27	0.94, 1.72	0.125
3 or more	1.17	0.74, 1.86	0.506
Mother's BMI			
[18.5, 25)	1.00		
< 18.5	1.13	0.89, 1.44	0.325
>= 25.0	0.61	0.50, 0.75	<0.001
Mother's education level			
None	1.00		
Primary	1.05	0.78, 1.42	0.738
Secondary	0.85	0.63, 1.14	0.276
Higher	0.95	0.61, 1.48	0.821
Father's education level			
No education	1.00		
Primary	0.89	0.75, 1.05	0.168
Secondary	0.71	0.56, 0.91	0.006
Higher	0.53	0.37, 0.76	<0.001
Mother's employment status			
Not-working	1.00		
Currently working	1.21	1.02, 1.44	0.032
Mother’s age difference to the father	0.99	0.98, 1.00	0.129
Wealth index			
Poorest	1.00		
Poorer	0.89	0.73, 1.07	0.214
Middle	0.73	0.59, 0.90	0.004
Richer	0.58	0.45, 0.75	<0.001
Richest	0.35	0.25, 0.49	<0.001
Area of Residence			
Rural	1.00		
Urban	1.33	1.06, 1.65	0.012
Administrative division			
Dhaka	1.00		
Barishal	1.12	0.86, 1.45	0.414
Chattogram	1.19	0.96, 1.48	0.109
Khulna	0.67	0.52, 0.86	0.002

Rajshahi	0.66	0.52, 0.82	<0.001
Rangpur	0.88	0.67, 1.16	0.365
Sylhet	1.57	1.27, 1.94	<0.001

7. Table 1: Mothers education level: Does it mean "No formal education"?

Authors' response:

For mother's education in Table 1 the category "No education" indeed meant "No formal education" and has been used as the reference category in the logistic regression. We have added the following line of clarification in the 'Independent variables' subsection of the 'Materials and Methods' section where mother's education was introduced [line 164-165].

"It is important to mention that the 'no education' category for both parents' education implies 'no formal education."

8. Discussion: Can this be explained that how maternal BMI and birth order and current work status of mothers are linked to childhood stunting?

Authors' response:

We have now added possible explanations for the association found between childhood stunting and the above-mentioned factors with references in the 'Discussion' section.

The main arguments are as follows:

1) A mother's BMI is representative of her own physical attributes, stature and nourishment. It is, therefore, not surprising that we found children having low-BMI mothers to be at higher risk of stunting.

2) Research found that higher order children are often subject to shorter duration of breastfeeding, lower prenatal and postnatal investments and lower care-time from parents. These probably explain the association between stunting and age and birth order - a result we found and also confirmed by existing research.

3) It may seem unusual that children of working mothers appeared to be more likely to be stunted. However, as reported in the 'Results' section, higher percentages of both poor and illiterate individuals in the working-mother sample may partly explain this association. However, the cross-sectional nature of the data do not allow us to venture further into the probable causes. The relevant references are not listed here, but are provided in the revised manuscript.

Reviewer #6:

Review report of the manuscript no. PONE-D-20-31337

Abstract section, the result is not well explained because it was not fulfill to the objectives. I think it was much differ to objectives and results.

Authors' response:

We thank the reviewer for this comment. We have now included the key findings in the abstract and excluded some policy suggestions in order to make it more informative and reflective of the objectives. The main changes that are marked in red in the revised manuscript are as follows:

"*Results revealed that, as individual factors, living in Sylhet division (OR: 1.57; CI: 1.26 - 1.96), being an urban resident (OR: 1.28; CI: 1.03 - 1.96) and having working mothers (OR: 1.21; CI: 1.02 - 1.44) were associated with higher likelihoods of childhood stunting, whereas belonging to the richest households (OR: 0.56; CI: 0.35 - 0.90), higher BMI of mothers (OR: 0.68 CI: 0.56 - 0.84) and mothers' involvement in decision making about children's healthcare with father (OR: 0.83, CI: 0.71 - 0.97) were linked to lower likelihoods of stunting. Importantly however, risk classifications defined by the interplay of multiple sociodemographic factors showed more extreme odds ratios (OR) of stunting than single factor ORs. For example, children aged 14 months or above who belong to poor wealth class, have lowly educated fathers and reside in either Dhaka, Barisal, Chittagong or Sylhet division are the most vulnerable to stunting (OR: 2.52, CI: 1.85 - 3.44).*"

Authors could not make clear in a good way what authors were said to understand the line 4-8

Authors' response:

We have now modified some sentences and added reference to improve the clarity of the points we made in the mentioned lines. Changes are marked in red in the revised manuscript.

28-31 lines were not obviously comprehend.

Authors' response:

We are sorry for any unclarity from our part. In the mentioned lines we tried to explain that since childhood stunting had been a prevalent health problem in Bangladesh it attracted much academic research. Major area of investigation of these studies were identification of socio-demographic risk factors of childhood stunting.

42-46 lines not satisfied for me and eventually objectives were not associated with the title.

Authors' response:

We have added the following subsequent texts to the mentioned lines in order to clarify our objectives in a better way.

"A major contribution of the paper is methodological which involves utilizing a classification tree, a method borrowed from the machine learning arena, to automatically detect the complex interactions among the common risk factors of childhood stunting. Importance of these interactions are then evaluated through the use of classical logistic regression models. Interplay of multiple factors show higher association with stunting and provide deeper insight to the persistent public health problem of the country."

Please also note that we have substantially curtailed the 'Introduction' by moving parts of it to the 'Discussion section following further suggestions. We hope it now communicates our objectives in a better and precise way.

49-55 lines explain more but it was not fruitful of the title. Even if the authors were not able to present it properly.

Authors' response:

We thank the reviewer for this comment and apologize for the lack of clarity from our part. Please note that the entire paragraph has now been removed from the 'Introduction' to make it more concise. Some of the arguments in the paragraph, particularly those about relative advantages of classification tree over classical regression models, are moved to the 'Methodology' section. The additions are marked in red in the revised manuscript.

66-77 lines authors wrote extend but it was repeated. Over all the introduction thrives could not well describe and inadequate.

Authors' response:

We understand the reviewer's concern. We want to iterate that following further suggestions for improving the lack of clarity of the 'Introduction' and making it concise, we have modified the section substantially. Thus, the mentioned texts are removed from the 'Introduction' and moved to the 'Discussion' section of the revised manuscript.

106-107 lines what were authors said not clear for me.

Authors' response:

We thank teh reviewer for this comment. We have modified the sentence as follows to improve its clarity:

"In addition to a number of health and nutrition relation information, the BDHS survey} also collected measurements on height and weight from eligible women of reproductive ages and children under the age of 5 years."

Responsive variable portion wrote too much and I thought easily and curtly discuss. Same case as independent variables elucidate too much and unnecessary.

Authors' response:

We understand the reviewer's observation. We have now deleted the entire first paragraph of the 'Response variable' (now changed to 'Outcome variable' in the revised manuscript) to make the subsection more concise. However, we believe some small details were necessary to clearly introduce the nature and measurement of the various explanatory variables with their different categories/values and therefore, we could not curtail the 'Independent variable' section. We hope the reviewer will understand our point.

147-206 methodology section wrote enlarge and few were repeated one or more times. Which had no resemblance to this manuscript?

Authors' response:

While we understand the reviewer's concern, we would like to emphasize that the methodology we used in this paper, particularly the machine learning method and its application in conjunction with classical statistical models, is a relatively new concept in public health research and we wanted to make sure that it is described with adequate clarity. We, therefore, felt that the classification tree with its relative advantages and disadvantages, generation of binary rules from its interaction results, model selection criteria including cross-validation, adjustment of results for survey weights, statistical packages used etc. be touched upon and introduced in proper sequence. We hope the reviewer will understand our inability to shorten the 'Methodology' section despite our best efforts due to the trade-off between length and clarity. We also want to point out that we avoided description of unnecessary details of familiar methods such as the logistic regression.

What were the eligibility criteria? On the whole data sources and materials portion is pitiable presentation.

Authors' response:

We have reported the eligibility criteria in the ‘Data Overview’ subsection. Precisely, children under 5-years of age whose anthropometric measures of stunting (height-for-age) were available were considered for the study, but de facto residents (temporary residents), twin births and cases with missing observations on other information were excluded.

235-239 lines I didn't understand what were authors want to recognize.

Authors’ response:

In these lines we were describing left part of the classification tree originated from the root. The subsequent appearance of the variables from the root indicated the order of importance. Thus, wealth is the most important predictor of stunting as it is placed at the top. Age is the next significant predictor with children aged below 14 months have lower prevalence of stunting. In order to relate the texts to the classification tree (Figure 1) better, we now included the prevalence of stunted children (60%) for the node explained.

241-250 lines were not clearly arrangement.

Authors’ response:

We have made the following modifications in the referred texts in order to improve clarity.

“The right-hand branch originated from the root of the classification tree include the wealthier households. On this side, mother’s education, and then their body mass index (BMI) as well as the age of a child were notable predictors of stunting. Here, children who have mothers with no/low formal education, who are older than 9 months and have relatively lower BMI (node 21) demonstrate high risk of child stunting (around 48%).”[lines 303-307].

Changes are marked in red.

Lines 380 to 400. I didn't think these findings are relevant. The really relevant results are that the only educational level that seems to be protective is the highest level of education, as well as the only socioeconomic level is the richest.

Authors' response:

We would like to point out that the referred texts at lines 380-392 of the original manuscript are merely policy implications of our findings rather than findings themselves, and apologize for any lack of clarity on this matter from our part. Please note that we have now modified the possible policy values in an attempt to make it more relevant to our results following suggestions from other reviewers [lines 487-502]..

We, however, agree with the reviewer that the emphasis we gave to the established association between mother's decision making on child's healthcare and childhood stunting in lines 394-405 of the original manuscript may not need separate attention as such as it can be treated as a secondary result not particularly relevant to the interactions we highlighted. We have now curtailed the details of this finding and moved it to the part of the 'Discussion' section where individually significant variables were discussed [lines 449-454].

Discussion portion could not present appropriately. In many cases authors could enlighten haphazardly. Authors could have accessible it in a simpler way. Authors end up writing it shortly.

Authors' response:

Please note that we have now modified the 'Discussion' section by including parts from the introduction and adding clarifying explanations in order to make it as coherent and relevant as possible. All the changes are marked in red in the revised manuscript.

All in all, authors could not nearby it well. But their title of the manuscript is very good and the methods that authors like are definitely appropriate for this time. But overall presentation is not appropriate of the manuscript.

Authors' response:

We appreciate the reviewer's encouraging words. We have now put substantial efforts to make the presentations of the paper as coherent and as organized as possible.

Reviewer #7:

It is good to see that the researchers considered a number of independent variables at children's individual, parental and household level. These factors are useful for understanding the childhood stunting from socioeconomic point of views. However, there are some important issues such as low birth weight (LBW), infant and young child feeding (early initiation of breastfeeding, exclusive breastfeeding, breastfeeding continuation, and complementary feeding) which the nutrition professionals and policy makers are highly concerned about. Could the authors please consider including these factors into analysis or provide justification why these factors were not considered?

Authors' response:

We understand the reviewer's rightful concern for not including the above-mentioned nutrition-related variables. Our justifications for not considering the factors are as follows:

First, we found selecting the possible set of explanatory variables for childhood stunting in our study particularly challenging. Existing studies did not help much in guiding to a common or universally accepted set of indicators, and vary substantially in terms of the number and types of variables that range from demographic and socio-economic factors to spatial and health-nutrition related factors. The main aim of our paper is to better understand the role of socio-demographic risk factors of childhood stunting through their interactions identified through a machine learning method as opposed to identify and establish a comprehensive set of best predictors of stunting. Therefore, rather than considering all possible risk factors of childhood stunting (which are numerous), in this study we focused mainly on socio-demographic determinants of under-5 stunting and other control variables which commonly appeared significantly important in many relevant empirical studies, particularly those related to Bangladesh. For this reason, we were selective and did not consider the mentioned indicators, as well as many other likely determinants of childhood stunting such as number of visits by antenatal care provider and their qualifications, hygiene practices etc. We have listed this confined (as opposed to exhaustive) selection of explanatory variables as a primary limitation of our study in the 'Discussion' section.

Second, while reviewing the literature we found that many of our considered socio-demographic factors are closely related to the mentioned health and nutrition related indicators. For example, household wealth and/or mother's education has been consistently found to be associated with birthweight (e.g., Khan et al., 2018, Manyeh et al., 2016), complementary feeding (e.g., Dhami et al., 2019, Nkoka et al., 2018) and breastfeeding practices (e.g., Rahman et al., 2020), and possibly capture some of their association with stunting indirectly.

Third, since breastfeeding and complementary feeding requirements depend on age of children, investigating impact of these factors require age-stratified analysis which is beyond the scope of this study mainly looking at interactions of common socio-demographic factors on under-5 children as a whole.

Lastly, the interactions that would be considered in a joint sociodemographic-nutritional variable setting would be too large and require a more detailed approach. We believe such a study would require more in-depth data and analyses which would be out of the scope of this study. As mentioned previously, the main idea behind the study is to introduce a methodological contribution to the extant literature with the help of a machine learning method that is easily interpretable and allows the possibility to consider complex interactions between the variables largely ignored in typical public health studies.

We, however, understand the importance of looking at nutritional factors and plan to investigate the role of these factors and their interactions in a similar setting in a future study. We hope the reviewer would agree with our line of argument presented above.

Abstract: Kindly mention what type of variables were considered in the statistical model. Health Nutrition and Population Sector Program (HNPSP) mainly focuses on health and nutrition related indicators rather than socio-demographic aspects. Kindly re-phrase how the findings could be useful in national planning and policy making to reach the SDG target.

Authors' response:

We understand the reviewer's point and admit that it is naïve to link the findings of the paper directly to meeting of HNPSP and SDG targets. We have now excluded this sentence from the abstract. Rather than making direct policy suggestions we emphasize targeting vulnerable

children identified through distinct risk profiles and interactions of socio-demographic characteristics, learning more about their nutritional and other requirements and accordingly address multiple risk factors in designing policies to mitigate their stunting problems.

Discussion: "The complex interplay of multiple factors usually go unnoticed"—the interaction is well perceived by the nutrition experts, but the evidences are undocumented.

Authors' response:

Here, we wanted to emphasize that the complex interplay of multiple factors is not directly observable when investigating pertinent factors individually. These interactions are not of course empirically well-investigated. However, but we apologize for the lack of clarity of the statement. We have now modified the sentence as following, appreciating acknowledgment of interactions by nutrition experts and researchers.

"These insights stemming from the complex interplay of multiple factors is not directly observable when looking at the pertinent factors individually. While the importance of these interactions is well perceived by the nutrition experts, supporting empirical evidences are largely undocumented."

Conclusion: It seems the summary of every section. Kindly emphasize what the findings interpret which is well understood and sensitize relevant professionals from field level to policy level.

Authors' response:

We thank the reviewer for the suggestion. We have now curtailed and modified the conclusion to highlight the results and their policy values. All changes are highlighted in red in the revised manuscript.

Reviewer #8:

Response- Title needs to be enreach and shorter, type of study needs to be mentioned in the title.

Authors' response:

We thank the reviewer for the suggestion. We, however, find it difficult to keep a balance between keeping the title short and adding more information to it, e.g., the type of study. Since the major contribution of the paper is methodological, we wanted to highlight the use of machine learning method in the title. We hope the reviewer will understand our point.

The findings is not elaborately mentioned in the abstract, result of OR need to be more specifically mentioned in abstract, The statistical analysis is not enough in the abstract.

Authors' response:

We fully agree with the reviewer and apologize for under-reporting key results in the abstract. We have now included more detailed findings with ORs as advised which are as follows:

"*Results revealed that, as individual factors, living in Sylhet division (OR: 1.57; CI: 1.26 - 1.96), being an urban resident (OR: 1.28; CI: 1.03 - 1.96) and having working mothers (OR: 1.21; CI: 1.02 - 1.44) were associated with higher likelihoods of childhood stunting, whereas belonging to the richest households (OR: 0.56; CI: 0.35 - 0.90), higher BMI of mothers (OR: 0.68 CI: 0.56 - 0.84) and mothers' involvement in decision making about children's healthcare with father (OR: 0.83, CI: 0.71 - 0.97) were linked to lower likelihoods of stunting. Importantly however, risk classifications defined by the interplay of multiple sociodemographic factors showed more extreme odds ratios (OR) of stunting than single factor ORs. For example, children aged 14 months or above who belong to poor wealth class, have lowly educated fathers and reside in either Dhaka, Barisal, Chittagong or Sylhet division are the most vulnerable to stunting (OR: 2.52, CI: 1.85 - 3.44).*"

"

References

Dhami, M.V., Ogbo, F.A., Osuagwu, U.L. and Agho, K.E., 2019. Prevalence and factors associated with complementary feeding practices among children aged 6–23 months in India: a regional analysis. *BMC public health*, *19*(1), pp.1-16.

Khan, J.R., Islam, M.M., Awan, N. and Muurlink, O., 2018. Analysis of low birth weight and its co-variants in Bangladesh based on a sub-sample from nationally representative survey. *BMC pediatrics*, *18*(1), pp.1-9.

Manyeh, A.K., Kukula, V., Odonkor, G., Ekey, R.A., Adjei, A., Narh-Bana, S., Akpakli, D.E. and Gyapong, M., 2016. Socioeconomic and demographic determinants of birth weight in southern rural Ghana: evidence from Dodowa Health and Demographic Surveillance System. *BMC pregnancy and childbirth*, *16*(1), pp.1-9.

Nkoka, O., Mhone, T.G. and Ntenda, P.A., 2018. Factors associated with complementary feeding practices among children aged 6–23 mo in Malawi: an analysis of the Demographic and Health Survey 2015–2016. *International health*, *10*(6), pp.466-479.

Rahman, M.A., Khan, M.N., Akter, S., Rahman, A., Alam, M.M., Khan, M.A. and Rahman, M.M., 2020. Determinants of exclusive breastfeeding practice in Bangladesh: Evidence from nationally representative survey data. *Plos one*, *15*(7), p.e0236080.

Sarker, A.R., Akram, R., Ali, N. and Sultana, M., 2019. Coverage and factors associated with full immunisation among children aged 12–59 months in Bangladesh: insights from the nationwide cross-sectional demographic and health survey. *BMJ open*, *9*(7), p.e028020.

Yaya, S., Bishwajit, G. and Gunawardena, N., 2019. Socioeconomic factors associated with choice of delivery place among mothers: a population-based cross-sectional study in Guinea-Bissau. *BMJ global health*, *4*(2), p.e001341.
