# Supplementary material for: Sociodemographic risk factors of under-five stunting in Bangladesh: Assessing the role of interactions using a machine learning method
Source: PLoS One. 2021 Aug 31;16(8):e0256729. doi: 10.1371/journal.pone.0256729 (PMC8407547; doi:10.1371/journal.pone.0256729)
Supplement: Attachment — Submitted filename: Review Report_PONE-D-20-31337.doc [file pone.0256729.s003.doc]

**Review report of the manuscript no. PONE-D-20-31337**

Abstract section, the result is not well explained because it was not fulfill to the objectives. I think it was much differ to objectives and results.

Authors could not make clear in a good way what authors were said to understand the line 4-8

28-31 lines were not obviously comprehend.

42-46 lines not satisfied for me and eventually objectives were not associated with the title.

49-55 lines explain more but it was not fruitful of the title. Even if the authors were not able to present it properly.

66-77 lines authors wrote extend but it was repeated.

Over all the introduction thrives could not well describe and inadequate.

106-107 lines what were authors said not clear for me.

Responsive variable portion wrote too much and I thought easily and curtly discuss.

Same case as independent variables elucidate too much and unnecessary.

147-206 methodology section wrote enlarge and few were repeated one or more times. Which had no resemblance to this manuscript?

What were the eligibility criteria? On the whole data sources and materials portion is pitiable presentation.

235-239 lines I didn't understand what were authors want to recognize.

241-250 lines were not clearly arrangement.

Lines 380 to 400. I didn't think these findings are relevant. The really relevant results are that the only educational level that seems to be protective is the highest level of education, as well as the only socioeconomic level is the richest.

Discussion portion could not present appropriately. In many cases authors could enlighten haphazardly. Authors could have accessible it in a simpler way. Authors end up writing it shortly.

All in all, authors could not nearby it well. But their title of the manuscript is very good and the methods that authors like are definitely appropriate for this time. But overall presentation is not appropriate of the manuscript.
