# Supplementary material for: Sociodemographic risk factors of under-five stunting in Bangladesh: Assessing the role of interactions using a machine learning method
Source: PLoS One. 2021 Aug 31;16(8):e0256729. doi: 10.1371/journal.pone.0256729 (PMC8407547; doi:10.1371/journal.pone.0256729)
Supplement: Attachment — Submitted filename: Manuscript status.docx [file pone.0256729.s005.docx]

**Revision of Manuscript (REF: PONE-D-20-31337)**

First of all I would like to thanks all authors. They extremely work hard because they check and revised all of my comments according to lines and numbers. Truly all authors deserve praise. Now I am happy to say that the revised manuscript should be ACCEPTED.
